# Global epidemiology of neonatal herpes: systematic review, meta-analyses, and meta-regressions

**DOI:** 10.7189/jogh.16.04104

**Published:** 2026-03-20

**Authors:** Arwa Saed Aldien, Manale Harfouche, Asalah Alareeki, Hiam Chemaitelly, Laith J Abu-Raddad

**Affiliations:** 1 Cornell University, Weill Cornell Medicine-Qatar, Infectious Disease Epidemiology Group, Doha, Qatar; 2 Cornell University, Weill Cornell Medicine, Department of Population Health Sciences, New York, New York, USA; 3 QU Health, Qatar University, College of Health Sciences, Department of Public Health, Doha, Qatar; 4 College of Health and Life Sciences, Hamad bin Khalifa University, Doha, Qatar

## Abstract

**Background:**

Neonatal herpes simplex virus (nHSV) infection, caused by HSV-1 or HSV-2, is a global health concern due to its high mortality and long-term morbidity. In this study, we assessed nHSV global epidemiology, regional variations, and temporal trends.

**Methods:**

We conducted a systematic review of PubMed, Embase, and national surveillance reports through 12 December 2024, and reported findings in accordance with PRISMA guidelines. We used random-effects meta-analysis to estimate pooled mean outcomes and meta-regression analyses to assess associations, temporal trends, and potential sources of heterogeneity.

**Results:**

We identified 143 relevant publications from three of the six World Health Organization regions, providing 140 nHSV incidence rate measures and 103 proportions of incident nHSV-1 *vs*. nHSV-2 cases. The global pooled and regional population-weighted mean incidence rate was 8.2 (95% confidence interval (CI) = 5.9–10.7) per 100 000 live births. Incidence rate was highest in the Americas (13.3 cases per 100 000 live births; 95% CI = 9.9–17.2), followed by the European Region (5.2 cases per 100 000 live births; 95% CI = 3.4–7.3) and the Western Pacific Region (2.9 cases per 100 000 live births; 95% CI = 2.2–3.6). Globally, nHSV-1 and nHSV-2 accounted for pooled and weighted means of 47.3% (95% CI = 39.5–55.0) and 52.8% (95% CI = 45.2–60.5) of cases, respectively. The highest nHSV-1 proportion was in the Western Pacific Region (57.7%; 95% CI = 49.2–66.1), while the highest nHSV-2 proportion was in the Region of the Americas (60.5%; 95% CI = 55.8–65.1). Meta-regression analyses showed an annual increase of 3.5% (95% CI = 1.5–5.6) in nHSV incidence rate, alongside a yearly 1.4% (95% CI = 0.9–1.9) increase in the proportion of nHSV-1 cases and a 1.1% (95% CI = 0.6–1.6) decrease in the proportion of nHSV-2 cases.

**Conclusions:**

nHSV affects approximately one in 10 000 newborns, with regional variations and a rising incidence rate. The increasing dominance of nHSV-1 over nHSV-2 reflects shifting HSV epidemiology.

Neonatal herpes simplex virus infection (nHSV) is a global health concern due to its severe clinical manifestations, potential for mortality, and risk of long-term morbidity [1–4]. The World Health Organization (WHO) estimates a global incidence rate of approximately 10 cases per 100 000 live births based on mathematical modelling [5]. Most cases (85%) are acquired perinatally through exposure to infected maternal genital secretions during childbirth, while in utero and postnatal transmission account for the remaining 15% [2,3]. Asymptomatic cervical viral shedding during delivery, particularly in individuals with primary or first-episode genital herpes, is the primary source of transmission [1–3,6].

nHSV can lead to severe neurological complications, disseminated multi-organ infection, and long-term developmental disabilities [2,3,7,8]. Clinical presentation typically occurs within the first four weeks of life and is categorised into three forms – skin, eye, and/or mouth (SEM), central nervous system (CNS), and disseminated disease [2–4]. CNS and disseminated forms account for approximately 30% and 25% of cases, respectively, with case-fatality rates of 50% and 85% if left untreated [1–3].

Managing nHSV imposes substantial health care costs, including prolonged hospitalisation, intensive monitoring, intravenous antiviral therapy, and extensive diagnostic testing [1,9–11]. Despite advancements in prevention, diagnosis, and antiviral treatment in high-income countries, neonatal mortality and neurological impairment remain high [1–4,7,11]. The burden is further exacerbated in low- and middle-income countries, where underdiagnosis remains a major challenge [1–4,7,11].

Moreover, nHSV is caused by infection with HSV type 1 (HSV-1) or HSV type 2 (HSV-2), both of which are globally prevalent [12,13]. HSV-1 is primarily transmitted through oral contact, but can also be acquired genitally, while HSV-2 is predominantly spread through genital transmission [12,13]. According to WHO estimates, approximately 3.8 billion individuals aged 0–49 years were living with HSV-1 (oral and genital) in 2020, reflecting a global prevalence of 64.2% [12]. For HSV-2, an estimated 520 million individuals aged 15–49 years were infected, corresponding to a global prevalence of 13.3% [12].

Recent shifts in the epidemiology of these infections have been documented. While the prevalence of genital HSV-2 appears to be slowly declining [14–19], the prevalence of genital HSV-1 is rising in several regions [20–24]. This suggests an ongoing epidemiological transition, with HSV-1 increasingly acquired genitally rather than orally [23,25,26]. These evolving trends in HSV epidemiology may affect the incidence of nHSV and the relative contribution of HSV-1 *vs*. HSV-2 as causative agents, potentially influencing the clinical manifestations and severity of nHSV. Nevertheless, the impact of these epidemiological shifts on nHSV remains uncertain.

To address this knowledge gap, our study presents, to our knowledge, the first systematic review and meta-analyses characterising the global epidemiology of nHSV. We estimated pooled mean incidence rates and the pooled mean proportions of incident nHSV cases attributable to HSV-1 and HSV-2. Additionally, we examined factors associated with these outcomes, identified temporal trends, and investigated sources of heterogeneity across studies. The overarching goal was to provide a comprehensive global overview of nHSV to support evidence-based decision-making by public health practitioners, policymakers, and researchers.

## METHODS

### Data sources and search strategy

We reviewed epidemiological evidence on nHSV informed by Cochrane Collaboration methods [27]. We reported findings in accordance with the PRISMA guidelines [28,29] (Table S1 in the **Online Supplementary Document****)**.

We conducted the literature search in PubMed and Embase from inception, with the last update on 12 December 2024. Additionally, we reviewed national-level surveillance reports from the International Network of Paediatric Surveillance Units, from inception through 12 December 2024, providing data from Australia, Britain, Canada, Germany, Ireland, and Switzerland [30].

The search strategy employed broad criteria, incorporating both index terms (expanded to include all relevant subheadings) and free-text terms, without restrictions on language or publication date (Box S1 in the **Online Supplementary Document**). The review covered all 194 WHO member countries, categorised into six regional classifications: African Region, Region of the Americas, Eastern Mediterranean Region, European Region, South-East Asia Region, and Western Pacific Region (Box S2 in the **Online Supplementary Document**).

The search strategy was designed to balance completeness and feasibility, enabling a comprehensive and methodologically sound synthesis of evidence on nHSV. Owing to the absence of standardised case definitions, diagnostic codes, and national notifiability in most countries [5,31,32], we used broad search terms to account for variability in terminology across studies. This entailed screening a large volume of citations to capture all relevant, representative, and quality data.

To ensure feasibility, we restricted the search to PubMed and Embase, the two primary international biomedical databases. Recognising that key data may lie outside the peer-reviewed literature, we also reviewed national surveillance reports. This dual approach – drawing from academic and programmatic sources – maximised data yield while preserving analytical rigour.

### Study selection process and inclusion and exclusion criteria

We imported search results into the reference manager EndNote (Thomson Reuters, Carlsbad, California, USA) and then into the systematic review management platform Covidence (Veritas Health Innovation, Melbourne, Victoria, Australia) for deduplication and screening.

Three authors (ASA, MH, and AA) screened titles and abstracts, followed by full-text screening to identify relevant publications. Screening was conducted independently in pairs. As the review was subsequently updated, each record was ultimately screened by at least two reviewers. Any discrepancies were resolved through consensus, with input from LJA. Case reports, case series, reviews, and editorials were excluded. The bibliographies of relevant publications and reviews were also screened to identify any additional potentially relevant reports.

The inclusion criteria required that relevant publications report primary data on nHSV incidence rate or the proportions of nHSV cases attributed to HSV-1 *vs*. HSV-2. nHSV cases had to be identified using valid, objective, and standardised criteria within a well-defined population, over a specified timeframe and catchment area. All cases were required to be confirmed through laboratory testing or clinical diagnosis, typically corroborated by laboratory results.

However, not all studies provided specific details on case identification methods or the type of laboratory testing used, particularly in national surveillance reports, which focused on reporting surveillance results rather than describing surveillance methods. In such cases, we classified the method of ascertainment as unclear. Nonetheless, we considered these data generally valid, as the studies were conducted within surveillance platform contexts in which case definitions and diagnostic practices align with accepted national standards and applicable clinical guidelines.

In some cases, in the included studies, diagnoses could have been made solely on clinical criteria without laboratory confirmation. However, this is not a methodological flaw, but rather a reflection of real-world clinical and diagnostic constraints associated with nHSV. Diagnosis of this condition is inherently challenging due to its broad clinical spectrum and variable presentation in newborns [1,2,33]. Laboratory confirmation requires that the clinical form be amenable to testing – such as the presence of vesicular lesions or accessible cerebrospinal fluid (CSF) – which may not always be present or obtainable at the time of evaluation. In such cases, clinicians may rely on a combination of clinical findings and other supportive diagnostic tools such as imaging.

We excluded studies if they defined nHSV outside the standard diagnostic period (zero to 90 days), did not test multiple sites to confirm nHSV diagnosis (*e.g.* CSF, blood, vesicles), or used unreliable methods such as serological immunoglobulin M/immunoglobulin G antibody testing alone or magnetic resonance imaging/computed tomography findings suggestive of conditions resembling nHSV in newborns, such as encephalitis or meningitis. Accurate laboratory detection of nHSV was considered to require polymerase chain reaction (PCR) or viral culture from swabs of vesicular lesions, blood, and/or CSF. Proportions of nHSV cases attributed to HSV-1 *vs*. HSV-2 were considered valid only if determined through laboratory-confirmed typing.

This systematic review approach reflects the broader epidemiological principle that disease burden estimates must often be grounded in the best available evidence, including data generated under real-world clinical and public health conditions. By including cases diagnosed using standardised and field-appropriate clinical criteria in a given setting – while strictly excluding low-quality or speculative data – this review provides a representative synthesis of the global nHSV burden.

In this systematic review, the term ‘report/publication’ refers to any document, such as an article or public health report, that includes an nHSV outcome measure. The term ‘study’ specifically refers to an nHSV outcome measure within a defined population. Duplicate findings from the same study were included only once, prioritising the publication with the most detailed or most recent data. For example, surveillance reports often represent results from previous years in each update; in such cases, data were extracted from the most recent report containing relevant information.

### Data extraction

Overall outcome measures (*i.e.* those covering the entire sample), along with their stratifications and associated variables, were extracted and double-extracted by ASA, AA, MH, and HC from relevant reports. Data extraction was conducted independently in pairs. Due to complexities in the included studies, at least three authors independently extracted data for most studies (Box S3 in the **Online Supplementary Document**). Any discrepancies were resolved through consensus, with input from LJA.

We defined the incidence rate as the proportion of nHSV cases among all live births in a given population, reported per 100 000 live births. Notably, although this epidemiologic measure is conventionally referred to as an ‘incidence rate,’ it is statistically defined and calculated as a proportion.

If the number of live births was not explicitly reported in a study, we calculated it as the number of nHSV cases divided by the reported incidence rate. In rare instances in which both the number of live births and the number of cases were not reported but the incidence rate was provided, we estimated the number of live births using the median value from other studies conducted in the same geographic area. We extracted nHSV case numbers when reported. Otherwise, we calculated them by multiplying the incidence rate by the number of live births. If a study did not provide a 95% confidence interval (CI) for the incidence rate, we calculated it using a Bayesian approach with a Beta-binomial model.

If multiple methods were used to estimate the incidence rate, only the estimate that the original authors considered most representative was extracted. This situation typically arose when statistical methods, such as capture-recapture, were applied to reported cases from multiple data sources to derive adjusted estimates [34]. When incidence rates were reported separately for symptomatic cases and for symptomatic and asymptomatic cases combined, we extracted only the measure for symptomatic cases, as the study focused on clinical cases of nHSV.

Stratified incidence rate measures were extracted and analysed when available on a year-by-year basis. If such data were unavailable, we used the overall measure. In some cases, stratified incidence rates for specific geographic regions (*e.g.* a single state or a combination of states) were included instead of year-by-year estimates when the latter contained zero cases or an insufficient number of cases to yield statistically reliable estimates.

We categorised estimates based on the level of surveillance – national, regional, or institutional. We classified sites where nHSV cases were examined as national surveillance registries, clinical settings, health care databases, or virological laboratories.

We defined the proportion of nHSV-1 (or nHSV-2) as the number of nHSV cases attributable to HSV-1 (or HSV-2) divided by the total number of typed nHSV cases in the study. If a case tested positive for both HSV-1 and HSV-2, it was counted in both categories. Consequently, the proportions attributed to HSV-1 and HSV-2 might not always sum to 100%.

### Critical appraisal of studies

We conducted a critical appraisal of the quality of all included studies for both the incidence rate and nHSV type proportion outcomes. Informed by established methods for assessing the quality of evidence in systematic reviews, and considering that this review did not evaluate management strategies or clinical interventions [35], we assessed the study quality using the Joanna Briggs Institute’s critical appraisal tool [36,37], with additional guidance from the Cochrane approach [27].

Four authors (ASA, MH, AA, and HC) independently conducted the assessments in pairs. Due to the complexities of the included studies, at least three authors independently assessed most of them. Any discrepancies were resolved through consensus, with input from LJA.

The quality of each study outcome was assessed across 10 risk of bias (ROB) domains: sample representativeness, participant recruitment, sample size, description of study subjects and settings, data analysis coverage, case definition criteria, reliability of diagnostic methods, statistical analysis methods, confounding factors, and criteria for subpopulations [36–39]. For each domain, the ROB was classified as either low or high (Table S2 in the **Online Supplementary Document**).

Given the stringent inclusion criteria for study outcome measures, most ROB domains automatically met the low ROB classification for all study outcomes. However, outcomes were assigned a high ROB in the case definition criteria domain if neonatal status was unclear or inadequately defined (*e.g.* lacking specification of the neonatal period during which nHSV was identified). Similarly, outcomes were classified as high ROB in the diagnostic reliability domain if the method of diagnosing nHSV was not clearly described (*e.g.* unspecified or unclear clinical/laboratory methods).

For the nHSV type outcome, we classified the sample size domain as low ROB if typed nHSV cases constituted at least 50% of all diagnosed nHSV cases. We synthesised the ROB assessments to evaluate the overall quality of the included studies and integrated them into the meta-regression analyses to examine their potential influence on study outcomes, in line with established approaches [15,40–45].

### Assessment of publication bias

We evaluated publication bias using Doi plots and the Luis Furuya-Kanamori (LFK) index [46]. An asymmetrical Doi plot suggested potential publication bias, indicating that variability in pooled outcome measures might not be due to chance alone [46]. An LFK index value exceeding plus/minus one was interpreted as evidence of some degree of publication bias [46].

In instances where potential publication bias was detected, we performed a sensitivity analysis by generating Doi plots and calculating LFK index values separately for each region. This analysis aimed to determine whether the observed asymmetry reflected true publication bias or was driven by natural variation in the number of live births and nHSV incidence rate across regions and countries.

### Meta-analyses

We summarised the extracted proportion measures using medians (MDs) and interquartile ranges (IQRs) and pooled using the DerSimonian-Laird random-effects model [47], incorporating the Freeman-Tukey double arcsine transformation to stabilise variances [48,49], after assessing its applicability to the pooled data [50].

We conducted sensitivity analyses to assess the robustness of the pooled nHSV incidence rate. First, we conducted a sensitivity analysis excluding studies that reported incidence rates without providing both the number of live births and the number of cases. Second, we performed a leave-one-out sensitivity analysis [51] to evaluate the influence of each individual measure on the overall pooled estimate. Third, we conducted meta-analyses using the Hartung-Knapp-Sidik-Jonkman method [52–54], which yields more conservative 95% CIs than the DerSimonian-Laird approach by more appropriately accounting for uncertainty in the estimated between-study variance. Fourth, meta-analyses were conducted using the restricted maximum likelihood method [55], which provides a more robust and less biased estimate of between-study variance.

In addition to calculating overall global and regional pooled measures, we calculated pooled mean estimates and 95% CIs for specific strata, provided that each stratum included at least three proportion measures. We generated forest plots to visually represent the pooled outcomes.

We also derived global estimates by weighting regional pooled estimates according to 2025 regional population sizes [56]. However, China’s population was excluded from the Western Pacific Region in these calculations, as no data were available from China despite it representing by far the largest population in the region [56].

We assessed heterogeneity using Cochran’s Q statistic (*P* <0.1) to confirm heterogeneity across studies [47,57]. In addition, the *I^2^* statistic was used to quantify the proportion of total variation across studies attributable to true between-study heterogeneity rather than sampling variation [47,57]. Prediction intervals were estimated to characterise the distribution of true values around the pooled mean [47,57]. We conducted meta-analyses in *R*, version 4.3.2 (R Core Team, Vienna, Austria) using the ‘meta’ package [58].

Given the heterogeneity observed in pooled outcome measures, pooled mean estimates should be interpreted as summary indicators reflecting averages across studies rather than as precise measures of underlying outcomes [15,40]. To systematically explore the sources of this heterogeneity, we conducted meta-regression analyses to identify epidemiologic and methodological factors associated with variation in outcome measures across studies. A key strength of this work is the granular assessment of heterogeneity and its drivers, encompassing both epidemiologic factors, such as geographic region, and methodological factors, such as the criteria used to define the neonatal period. Collectively, this analytic framework provides a nuanced understanding of the determinants of heterogeneity in the assessed outcomes and strengthens the interpretability of the pooled estimates.

### Meta-regressions

We conducted univariable and multivariable random-effects meta-regression analyses of log-transformed proportions to investigate and explain sources of between-study heterogeneity and to identify factors associated with study outcomes. Variables for the meta-regression analyses were determined a priori based on relevance and prior knowledge of nHSV epidemiology [1–5,10,11] (Box S4 in the **Online Supplementary Document**).

We included predictors with *P* ≤ 0.20 in the final multivariable model. However, because of interest in assessing temporal trends, the year of data collection was included in all multivariable models, regardless of *P*-value. Variables in the multivariable analysis with *P* ≤ 0.05 were considered statistically significant predictors. Model fit was assessed using the adjusted *R^2^* statistic, which represents the proportion of variance explained by the model after accounting for the number of predictors.

We applied two distinct multivariable models – one including the year of data collection as a categorical variable and the other as a continuous linear term. For studies in which data collection spanned multiple years, the midpoint of the study period was used as the reference year. Due to collinearity between the criteria for defining the neonatal period and the ROB domain for case definition, as well as between the method of ascertainment and the ROB domain for diagnostic reliability, the main analyses for incidence rate included the criteria for defining the neonatal period and the method of ascertainment, while sensitivity analyses replaced these variables with the corresponding ROB domains.

Because the observed increasing temporal trend in the meta-regression analysis for incidence rate is a central finding of this study, we conducted several additional sensitivity analyses to assess its robustness and to exclude potential methodological artefacts. We first conducted meta-regression analyses separately by site type, with each category evaluated independently while excluding the others. Analyses were then repeated by estimate type, again restricting each model to a single category. We performed additional analyses, including only studies with a well-defined neonatal period and, separately, only studies with a well-defined method of ascertainment. Finally, because some studies overlapped in time, location, or population – potentially violating the assumption of study independence – we applied a multilevel meta-analytic model [59] in place of the main analysis, explicitly accounting for within-study clustering.

For studies in which the data collection year was not reported, it was imputed using the publication year, adjusted by the median difference between the publication and data collection years from studies with complete data. As this procedure affected only a small number of studies reporting the proportions of nHSV cases attributable to HSV-1 and HSV-2, we conducted sensitivity analyses by excluding studies with imputed data collection years to evaluate the robustness of the observed temporal trends in these proportions. We performed meta-regressions in Stata, version 16.1 (StataCorp LLC, College Station, Texas, USA) using the ‘metareg’ package [60].

### Patient and public involvement

Patients or the public were not involved in the design, conduct, reporting, or dissemination plans of our research.

## RESULTS

### Search results and scope of evidence

The initial search yielded 13 126 records. Out of these, 4375 were from PubMed and 8751 from Embase. After removing duplicates, screening titles and abstracts, and conducting full-text reviews, 113 reports met the inclusion criteria. An additional three relevant reports were identified through bibliographic screening of included reports. The search of the International Network of Paediatric Surveillance Units database identified 27 additional relevant reports. In total, 143 reports were included in this systematic review.

**Figure 1:**
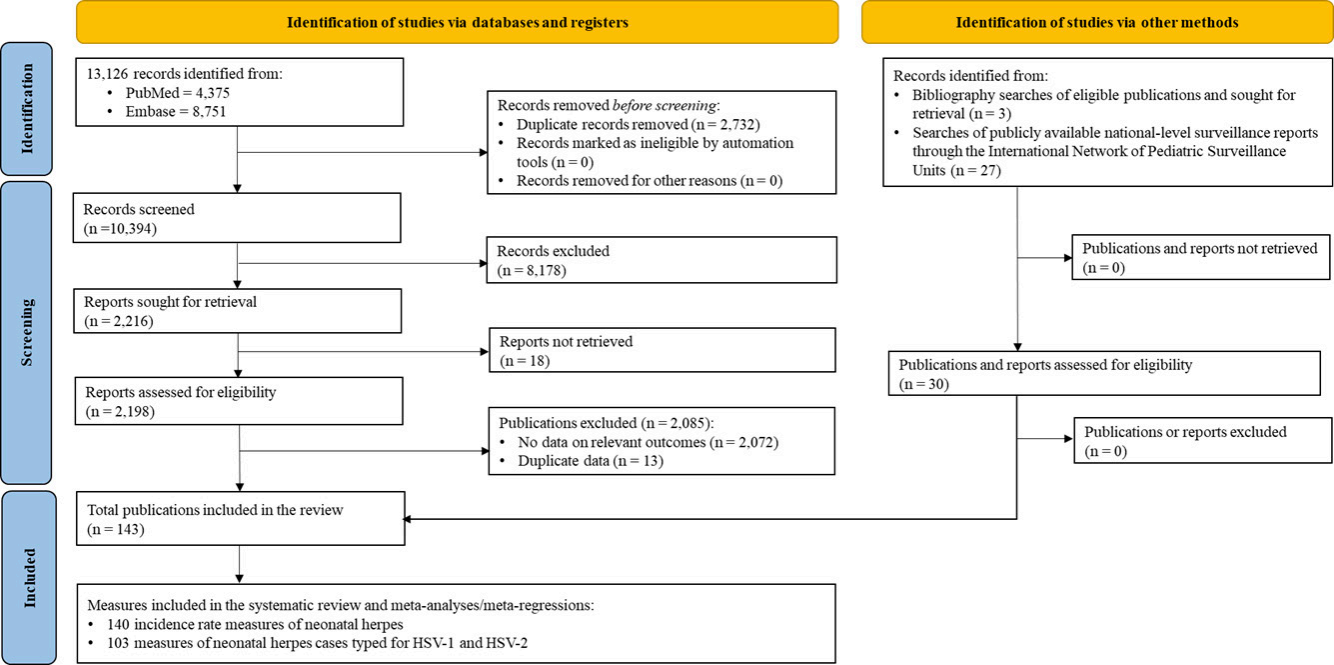
PRISMA flowchart illustrating the study selection process for investigating the epidemiology of neonatal herpes, in accordance with PRISMA guidelines [28,29]. nHSV – neonatal herpes simplex virus.

These reports provided 140 estimates of nHSV incidence rates and 103 estimates of the proportions of nHSV cases attributed to HSV-1 *vs*. HSV-2 (**Figure 1**; Table S3–4 in the **Online Supplementary Document**).

Data on study outcomes were available from three of the six WHO regions: the Americas, the European Region, and the Western Pacific Region. USA contributed the most data, with 72 nHSV incidence rate measures and 42 measures of nHSV-1 *vs*. nHSV-2 proportions, followed by Australia with 19 and 15 measures, respectively. The study timeframe spanned from 1969–2024, with approximately half of the data collected after 2005. Most studies defined the neonatal period as zero to 29 days rather than the broader zero to 90-day definition.

All studies reporting nHSV incidence rate (n = 140) explicitly reported the year(s) of data collection, and therefore required no imputation. By contrast, imputation was necessary for a small subset of studies reporting the proportions of nHSV cases attributable to HSV-1 and HSV-2 (eight of 103; 7.8%). The median interval between data collection and publication for nHSV proportion studies declined over time, from 9.5 years for studies conducted before 1995 to 7.3 years for those conducted between 1995 and 2004, and to 4.5 years for studies conducted from 2005 onward.

### Risk of bias assessments

ROB assessments indicated generally low to moderate risk of bias across studies reporting nHSV incidence rates and proportions of nHSV-1 *vs*. nHSV-2 (Figure S1–S4 in the **Online Supplementary Document**).

All studies across both outcomes were classified as low ROB in seven of the 10 domains, including sample representativeness, participant recruitment, description of study subjects and settings, data analysis coverage, statistical analysis methods, confounding factors, and criteria for subpopulations (Table S2 in the **Online Supplementary Document**).

These domains were judged to be at low risk of bias across all included studies largely because of the nature of the evidence base and the eligibility criteria applied (Table S2 in the **Online Supplementary Document**). Most data were derived from population-based surveillance systems, national registries, or large administrative or health care databases designed to capture all identified nHSV cases within clearly defined geographic catchment areas and time periods. As such, samples were inherently representative of the underlying source populations, participant recruitment was population-based rather than selective, and coverage of the target population was comprehensive by design. Moreover, study subjects and settings were consistently well described, as surveillance reports and registry-based studies necessarily specify the population denominator (*e.g.* live births) and the geographic scope and surveillance period required to enable incidence estimation.

Similarly, data analysis coverage and statistical methods were considered to be at low risk of bias because outcome measures were calculated using standard epidemiologic definitions and transparent numerator–denominator relationships, typically involving complete ascertainment of reported cases within the surveillance frame (Table S2 in the **Online Supplementary Document**). Confounding was not a concern for these descriptive outcomes, as incidence rates and etiologic proportions were not derived from comparative exposure – outcome analyses but from population-level counts. Criteria for subpopulations were also met, as stratifications (when reported) were based on predefined characteristics such as region or calendar period, rather than on post hoc or selectively defined subgroups.

In the case definition criteria domain, which pertains to how the neonatal period was defined, 43 (30.7%) of incidence rate studies and 24 (23.3%) of nHSV-1 *vs*. nHSV-2 proportion studies were classified as high ROB due to unclear definitions of the neonatal period.

In the diagnostic reliability domain, which assesses the methods used to confirm nHSV cases, 14 (10.0%) of incidence rate studies were classified as high ROB due to unclear diagnostic methods. In contrast, all nHSV-1 *vs*. nHSV-2 proportion studies were classified as low ROB, as HSV typing was performed using acceptable laboratory methods.

In the sample size domain, which assesses whether typed nHSV cases represented at least 50% of all diagnosed nHSV cases, seven (6.8%) of the proportion studies for nHSV-1 *vs*. nHSV-2 were classified as high ROB. In contrast, all incidence rate studies were classified as low ROB, as they included all identified nHSV cases within large populations of live births.

### Publication bias assessment

We found no evidence of publication bias for the proportions of nHSV-1 and nHSV-2, and both measures exhibited symmetrical Doi plots (Figure S5 in the **Online Supplementary Document**). However, nHSV incidence rate measures showed an asymmetrical Doi plot and an LFK index value exceeding plus/minus one, indicating potential publication bias (Figure S5 in the **Online Supplementary Document**).

To explore this further, we conducted a sensitivity analysis by generating Doi plots and calculating LFK index values for each region separately. This analysis revealed a substantial reduction in asymmetry, supporting the interpretation that the observed global asymmetry was primarily driven by natural variation in the number of live births and nHSV incidence rate across regions and countries, rather than true publication bias.

### Overview and pooled mean estimates of nHSV incidence rate

The median incidence rate of nHSV across studies was 6.9 per 100 000 live births (IQR = 2.9–12.4 per 100 000 live births) (**Table 1**). Given that most countries globally did not contribute nHSV incidence rate data, these estimates are derived from countries that together account for only 6.1% of all live births worldwide.

**Table 1:** Pooled mean estimates for the incidence rate of neonatal herpes*

Population classification	Outcome measures, n	Live births, n	Incidence rate per 100 000 live births, MD (IQR)	Pooled neonatal herpes incidence rate per 100 000 live births, x̄ (95% CI)	Heterogeneity measures			
					**Q†**	***P*-value**	***I*^2^ (95% CI)‡**	**Prediction interval, 95% CI§**
**WHO region**								
Region of the Americas	80	138 585 218	10.6 (5.3–27.8)	13.3 (9.9–17.2)	16 609.7	<0.001	99.5 (99.5–99.6)	0.0–63.8
European	33	43 985 903	4.7 (2.4–8.4)	5.2 (3.4–7.3)	954.1	<0.001	96.6 (96.0–97.2)	0.0–22.3
Western Pacific	27	18 902 664	3.0 (2.4–4.11)	2.9 (2.2–3.6)	162.6	<0.001	84.0 (77.8–88.5)	0.4–7.3
**Country**								
Australia	19	7 969 715	3.3 (2.7–5.0)	3.4 (2.5–4.5)	76.2	<0.001	76.4 (63.3–84.8)	0.5–8.3
Canada	8	1 102 051	5.9 (4.4–13.2)	6.5 (1.9–13.1)	13.5	0.061	48.1 (0.0–76.9)	0.0–28.5
Denmark	3	1 459 252	4.6 (3.5–6.8)	5.0 (1.9–9.4)	21.6	<0.001	90.7 (75.7–96.5)	0.0–158.7
Finland	1	240 000	NA	33.0 (26.1–40.7)	NA	NA	NA	NA
France	1	666 667	NA	1.2 (0.5–2.4)	NA	NA	NA	NA
Germany	1	1 574 468	NA	2.2 (1.7–3.2)	NA	NA	NA	NA
Israel	3	1 540 950	8.4 (7.2–8.3)	31.6 (0.0–147.4)	31.8	<0.001	93.7 (85.0–97.4)	0.0–7 510.4
Japan	8	10 932 949	2.4 (1.5–2.9)	2.1 (1.5–2.9)	43.6	<0.001	83.9 (70.0–91.4)	0.4–5.3
Netherlands	6	6 037 149	3.0 (2.5–4.3)	3.2 (2.4–4.2)	19.4	0.002	74.2 (41.2–88.7)	0.9–6.9
Spain	1	78 400	NA	8.9 (3.6–18.4)	NA	NA	NA	NA
Sweden	2	467 690	6.5 (NA)	6.6 (4.5–9.4)	NA	NA	NA	NA
Switzerland	2	444 007	0.8 (NA)	1.6 (0.6–3.2)	NA	NA	NA	NA
UK	13	31 477 320	6.9 (3.3–9.5)	5.2 (3.2–7.7)	653.1	<0.001	98.2 (97.6–98.6)	0.0–18.1
USA	72	137 483 167	11.2 (5.3–28.3)	14.0 (10.3–18.2)	16 584.3	<0.001	99.6 (99.5–99.6)	0.0–67.3
**Year of data collection category¶**								
<1995	43	29 290 482	5.9 (2.5–11.7)	5.9 (3.8–8.4)	664.3	<0.001	93.7 (92.3–94.8)	0.0–27.4
1995–2004	23	31 919 950	5.9 (3.9–10.1)	8.7 (4.4–14.5)	882.9	<0.001	97.5 (96.9–98.0)	0.0–51.5
≥2005	74	140 263 353	7.6 (3.0–15.8)	10.3 (7.3–13.8)	15 565.3	<0.001	99.5 (99.5–99.6)	0.0–56.7
**Year of publication category**								
<2000	32	13 122 212	7.9 (3.6–12.3)	7.6 (4.8–10.9)	459.0	<0.001	93.2 (91.5–94.7)	0.0–30.0
2000–2009	30	22 805 998	5.2 (2.8–8.7)	7.4 (4.0–11.7)	1146.8	<0.001	97.5 (97.0–97.9)	0.0–44.0
≥2010	78	165 545 575	7.2 (2.8–13.7)	9.7 (7.0–13.0)	16 107.9	<0.001	99.5 (99.5–99.6)	0.0–54.4
**All studies**	140	201 473 785	6.9 (2.9–12.4)	8.8 (6.9–10.9)	17 805.7	<0.001	99.2 (99.2–99.3)	0.0–46.8

CI – confidence interval, IQR – interquartile range, MD – median, NA – not applicable, WHO – World Health Organization, x̄ – mean

*A minimum of three measures was required to perform a random-effects meta-analysis. When only two measures were available, the arithmetic mean and its corresponding 95% CI were calculated. If only one study was available, the point estimate was reported with its 95% CI.

†Q – the Cochran’s Q statistic is a measure assessing the existence of heterogeneity in pooled outcome measures, here incidence rate.

‡*I*^2^ – A measure that assesses the magnitude of between-study variation that is due to true differences in incidence rate across studies rather than chance.

§Prediction interval – a measure that estimates the distribution (95% interval) of the true incidence rate around the estimated mean.

¶The categories were determined based on the median time between the year of publication and the year of data collection, which was approximately six years. This interval was rounded to five years to have a five-year bracket.

The Region of the Americas had the highest pooled mean estimate at 13.3 (95% CI = 9.9–17.2), followed by the European Region at 5.2 (95% CI = 3.4–7.3) and the Western Pacific Region at 2.9 (95% CI = 2.2–3.6) per 100 000 live births. While pooled estimates varied across countries, some had too few studies, limiting the representativeness of these estimates.

Globally, the pooled mean nHSV incidence rate, representing the best available estimate from the three reporting regions and based on all available measures, was estimated at 8.8 (95% CI = 6.9–10.9) per 100 000 live births. When regional pooled estimates were weighted by regional population sizes, the global incidence rate was estimated at 8.2 (95% CI = 5.9–10.7) per 100 000 live births.

Most of these meta-analyses showed significant heterogeneity (*P* < 0.1), attributable to true variation in incidence rates rather than random chance, as indicated by *I*^2^ values exceeding 50% (**Table 1**). This conclusion was further supported by the wide prediction intervals. The forest plot presenting the results of the meta-analyses is shown in Figure S6 in the **Online Supplementary Document**.

The pooled mean nHSV incidence rate was 6.4 (95% CI = 4.7–8.4) per 100 000 live births for studies with laboratory-based ascertainment and 8.4 (95% CI = 5.8–11.4) per 100 000 live births for studies using laboratory and/or clinical ascertainment, indicating that the two estimates are similar, with overlapping 95% CIs (Table S6 in the **Online Supplementary Document**).

In the sensitivity analysis, excluding studies that reported incidence rates without providing both the number of live births and the number of cases (n = 7 studies), the pooled mean global nHSV incidence rate was largely unchanged at 8.4 (95% CI = 6.6–10.4) per 100 000 live births. The leave-one-out sensitivity analysis [51] demonstrated that exclusion of any individual study had a negligible influence on the overall global pooled estimate (Table S7 in the **Online Supplementary Document**). Similarly, the sensitivity analyses using the Hartung-Knapp-Sidik-Jonkman method [52–54] and the restricted maximum likelihood method [55] yielded pooled estimates consistent with the main analysis, with overlapping 95% CIs (Table S6 in the **Online Supplementary Document**).

### Predictors of nHSV incidence rate and sources of between-study heterogeneity

All multivariable models explained more than 30% of the between-study variation in incidence rates. The univariable and multivariable meta-regression results supporting these findings are summarised in **Table 2**, with corresponding sensitivity analyses where the neonatal period definition and ascertainment method were replaced by their respective ROB domains (Table S8 in the **Online Supplementary Document**).

**Table 2:** Univariable and multivariable meta-regression analyses for the incidence rate of neonatal herpes

Characteristics	Outcome measures, n	Live births, n	Univariable analyses				Multivariable analyses			
			**Crude IRR (95% CI)**	***P*-value**	**LR test *P*-value**	Adjusted R^2^*	**Model 1, adjusted IRR (95% CI)**	***P*-value**	**Model 2, adjusted IRR (95% CI)**	***P*-value**
**WHO region**										
Region of the Americas	80	138 585 218	ref.		<0.001	14.2	ref.		ref.	
European	33	43 985 903	0.50 (0.30–0.82)	0.006			0.54 (0.30–0.99)	0.047	0.49 (0.27–0.90)	0.020
Western Pacific	27	18 902 664	0.30 (0.18–0.52)	<0.001			0.48 (0.24–0.96)	0.039	0.42 (0.21–0.84)	0.014
**Country†**										
USA	72	137 483 167	ref.		0.005	14.4	NA		NA	
Australia	19	7 969 715	0.36 (0.20–0.68)	0.002			NA		NA	
Canada	8	1 102 051	1.04 (0.34–3.17)	0.943			NA		NA	
Denmark	3	1 459 252	0.46 (0.11–1.83)	0.268			NA		NA	
Israel	3	1 540 950	1.89 (0.47–7.56)	0.363			NA		NA	
Japan	8	10 932 949	0.20 (0.08–0.48)	<0.001			NA		NA	
Netherlands	6	6 037 149	0.31 (0.12–0.84)	0.022			NA		NA	
UK	13	31 477 320	0.48 (0.23–0.96)	0.039			NA		NA	
Other countries‡	8	3 471 232	0.48 (0.19–1.22)	0.120			NA		NA	
**Site type**										
National surveillance registry/report	65	116 238 593	ref.		<0.001	26.4	ref.		ref.	
Clinical setting	35	9 049 159	3.17 (1.96–5.13)	<0.001			3.24 (1.69–6.20)	<0.001	3.56 (1.89–6.69)	<0.001
Healthcare database	24	70 439 796	5.23 (3.12–8.78)	<0.001			2.73 (1.33–5.63)	0.007	2.51 (1.24–5.08)	0.011
Virological laboratory	4	2 069 357	1.41 (0.45–4.43)	0.556			2.00 (0.58–6.86)	0.267	1.96 (0.60–6.37)	0.262
Mixed/unclear	12	3 676 880	2.14 (0.97–4.74)	0.061			2.56 (0.98–6.69)	0.055	2.72 (1.10–6.74)	0.031
**Estimate type**										
National	68	118 353 291	ref.		0.132	1.7	ref.		ref.	
Regional	57	81 882 934	1.27 (0.80–2.01)	0.311			1.21 (0.62–2.35)	0.578	1.35 (0.70–2.59)	0.370
Institutional	15	1 237 560	2.21 (0.99–4.95)	0.053			1.10 (0.42–2.87)	0.847	1.09 (0.43–2.75)	0.848
**Criteria for defining the neonatal period§**										
0–29 d	48	79 353 024	ref.		0.065	2.5	ref.		ref.	
0–90 d	49	91 433 509	0.54 (0.32–0.91)	0.020			0.76 (0.42–1.36)	0.346	0.64 (0.35–1.14)	0.129
Unclear	43	30 687 252	0.76 (0.44–1.32)	0.332			1.32 (0.79–2.20)	0.293	1.36 (0.83–2.22)	0.219
**Method of ascertainment§**										
Laboratory confirmation	71	91 709 649	ref.		0.001	9.4	ref.		ref.	
Laboratory and/or clinical confirmation	55	95 602 641	1.17 (0.75–1.83)	0.480			1.20 (0.72–1.98)	0.478	1.28 (0.80–2.05)	0.300
Unclear	14	14 161 495	4.23 (2.02–8.82)	<0.001			2.08 (0.98–4.41)	0.055	2.07 (1.00–4.28)	0.051
**Year of data collection category**										
<1995	43	29 290 482	ref.		0.850	0.0	ref.		NA	
1995–2004	23	31 919 950	1.19 (0.60–2.36)	0.607			1.54 (0.82–2.88)	0.175	NA	
≥2005	74	140 263 353	1.13 (0.68–1.88)	0.642			1.97 (1.11–3.49)	0.020	NA	
**Year of data collection**	140	201 473 785	1.00 (0.99–1.02)	0.710	0.710	0.0	NA		1.03 (1.01–1.06)	0.001

CI – confidence interval, IRR – incidence rate ratio, LR – likelihood ratio, NA – not applicable, R^2^ – coefficient of determination, ref – reference, WHO – World Health Organization

*Adjusted *R^2^* is a goodness-of-fit statistic indicating the proportion of variance explained by the model after accounting for the number of predictors. Adjusted *R^2^* in the final multivariable model 1 is 33.4%. Adjusted *R^2^* in the final multivariable model 2 is 37.3%.

†The country variable was excluded from the multivariable model due to collinearity with the region variable.

‡This category includes countries with two or fewer studies including Finland, France, Germany, Spain, Sweden, and Switzerland.

§Of the 10 risk of bias domains assessed in this systematic review, 8 were rated as low risk of bias across all studies and were therefore excluded from the meta-regression analyses. The only domains with variability were the ‘case definition criteria’ domain and the ‘diagnostic reliability’ domain. These domains were included in the sensitivity analyses presented in Table S8 in the **Online Supplementary Document** but were excluded from this main analysis table due to collinearity with the ‘criteria for defining the neonatal period’ and ‘method of ascertainment’ variables, respectively. To assess temporal trends, the year of data collection was included in the multivariable models irrespective of the *P*-value criteria for model inclusion.

The results showed that the nHSV incidence rate increased by 1.03-fold (95% CI = 1.01–1.06) per year, corresponding to an annual rise of 3.5% (95% CI = 1.5–5.6). Compared to the Region of the Americas, the European Region had a 0.49-fold (95% CI = 0.27–0.90) lower incidence rate, while the Western Pacific Region had a 0.42-fold (95% CI = 0.21–0.84) lower incidence rate.

Regarding the influence of study methods on observed incidence rates, studies classified as high ROB in the case definition criteria domain reported higher incidence rates than those classified as low ROB (Table S8 in the **Online Supplementary Document**). However, no statistically significant evidence was found that variations in the criteria for defining the neonatal period (**Table 2**), the method of nHSV case ascertainment (**Table 2**), or the ROB classification for the diagnostic reliability domain (Table S8 in the **Online Supplementary Document**) affected the observed incidence rates.

Nevertheless, estimates with unclear case ascertainment methods (**Table 2**) and those classified as high ROB for the diagnostic reliability domain (Table S8 in the **Online Supplementary Document**) showed a trend toward higher reported incidence rates compared to studies using standardised methodologies or classified as low ROB, respectively, with borderline statistical significance. Estimates derived from clinical settings or health care databases reported substantially higher incidence rates than those based on national surveillance registries or reports.

All sensitivity analyses conducted to evaluate the robustness of the observed temporal trend and to exclude potential methodological artefacts consistently indicated an increasing incidence rate over calendar time, with point-estimate incidence rate ratios exceeding one per year in all nine analyses (Figure S7 in the **Online Supplementary Document**). In some analyses, the trend did not reach statistical significance, reflecting reduced statistical power due to the smaller number of studies included, as these were subgroup analyses restricted to subsets of studies rather than the full data set.

### Overview and pooled mean estimates of nHSV-1 and nHSV-2 proportions

Across studies, the median proportion of nHSV-1 among nHSV cases was 46.2% (IQR = 30.9–62.5) (**Table 3**). Conversely, the median proportion of nHSV-2 was 53.8% (IQR = 37.5–69.1) (**Table 4**).

**Table 3:** Pooled mean proportion of neonatal herpes cases attributed to HSV-1*

Population classification	Outcome measures, n	Sample size, n	Proportion of nHSV-1 in %, MD (IQR)	Pooled mean proportion of nHSV-1 in %, x̄ (95% CI)	Heterogeneity measures				
					**Q**†	***P-*value**	***I*^2^ (95% CI)**‡	**Prediction interval (95% CI)§**
**WHO region**								
Region of the Americas	44	1831	40.06 (29.8–48.4)	39.6 (35.0–44.3)	124.0	<0.001	65.3 (52.3–74.8)	16.8–64.7
European	35	903	50.0 (29.3–71.6)	50.4 (39.6–61.2)	349.1	<0.001	90.3 (87.5–92.4)	1.8–98.5
Western Pacific	24	610	62.6 (50.0–70.6)	57.7 (49.2–66.1)	58.8	<0.001	60.9 (39.0–74.9)	24.3–88.1
**Country**								
Australia	15	388	62.7 (50.0–73.6)	55.5 (41.8–68.8)	49.1	<0.001	71.5 (51.9–83.1)	10.7–95.8
Canada	2	59	54.0 (NA)	59.5 (45.7–71.9)	NA		NA	NA
Denmark	3	62	50.0 (46.4–74.1)	76.8 (21.9–100.0)	15.1	0.001	86.7 (61.9–95.4)	0.0–100.0
Finland	1	13	NA	7.7 (0.2–36.0)	NA		NA	NA
France	1	8	NA	50.0 (15.7–84.3)	NA		NA	NA
Germany	1	24	NA	83.3 (62.6–95.2)	NA		NA	NA
Israel	4	110	54.9 (25.0–80.0)	54.8 (8.5–96.9)	27.1	<0.001	88.9 (74.3–95.2)	0.0–100.0
Japan	8	206	61.2 (52.2–65.5)	63.1 (56.1–69.9)	9.8	0.203	28.3 (0.0–67.8)	54.3–71.6
Netherlands	7	157	75.0 (60.9–83.6)	75.0 (59.7–87.9)	23.1	0.001	74.0 (44.5–87.8)	25.0–100.0
Republic of Korea	1	16	NA	62.5 (35.4–84.8)	NA		NA	NA
Sweden	6	201	22.2 (20.1–24.3)	21.3 (15.7–27.5)	3.5	0.632	0.0 (0.0–74.6)	13.5–30.3
Switzerland	1	6	NA	66.7 (22.3–95.7)	NA		NA	NA
UK	11	322	48.4 (37.2–53.3)	44.0 (37.4–50.7)	14.1	0.167	29.3 (0.0–65.2)	30.9–57.5
USA	42	1772	40.0 (29.3–47.3)	38.8 (34.2–43.5)	114.2	<0.001	64.1 (50.1–74.1)	16.9–63.2
**Year of data collection category¶**								
<1995	38	1211	32.1 (22.9–45.3)	34.1 (28.0–40.4)	128.9	<0.001	70.4 (58.9–78.7)	7.2–67.3
1995–2004	21	596	44.7 (32.1–62.5)	44.5 (33.5–55.7)	104.0	<0.001	80.8 (71.5–87.0)	4.2–89.4
≥2005	44	1537	54.2 (47.3–72.9)	59.2 (52.4–65.9)	255.6	<0.001	83.2 (78.2–87.0)	21.0–92.4
**Year of publication category**								
<2000	28	880	30.9 (22.2–43.5)	31.3 (25.1–37.9)	63.8	<0.001	57.7 (35.6–72.2)	8.3–59.7
2000–2009	26	667	42.8 (27.8–61.5)	43.2 (33.7–52.9)	131.0	<0.001	80.9 (72.8–86.6)	5.2–86.3
≥2010	49	1797	53.8 (44.8–72.2)	57.4 (50.8–63.9)	298.2	<0.001	83.9 (79.5–87.4)	18.5–92.0
**All studies**	103	3344	46.2 (30.9–62.5)	46.9 (42.0–51.8)	643.4	<0.001	84.1 (81.3–86.6)	8.9–86.9

CI – confidence interval, HSV-1 – herpes simplex virus type 1, IQR – interquartile range, MD – median, nHSV-1 – neonatal herpes attributed to herpes simplex virus type 1, NA – not applicable, WHO – World Health Organization, x̄ – mean

*A minimum of three measures was required to perform a random-effects meta-analysis. When only two measures were available, the arithmetic mean and its corresponding 95% CI were calculated. If only one study was available, the point estimate was reported with its 95% CI.

†Q – the Cochran’s Q statistic is a measure assessing the existence of heterogeneity in pooled outcome measures, here incidence rate.

‡*I*^2^ – a measure that assesses the magnitude of between-study variation that is due to true differences in incidence rate across studies rather than chance.

§Prediction interval – a measure that estimates the distribution (95% interval) of the true incidence rate around the estimated mean.

¶The categories were determined based on the median time between the year of publication and the year of data collection, which was approximately six years. This interval was rounded to five years to have a five-year bracket.

**Table 4:** Pooled mean proportion of neonatal herpes cases attributed to HSV-2*

Population classification	Outcome measures, n	Sample size, n	Proportion of nHSV-2 in %, MD (IQR)	Pooled mean proportion of nHSV-2 in %, x̄ (95% CI)	Heterogeneity measures			
					**Q**†	***P*-value**	***I^2^* (95% CI)**‡	**Prediction interval (95% CI)**§
**WHO Region**								
Region of the Americas	44	1831	59.4 (51.6–70.2)	60.5 (55.8–65.1)	121.8	<0.001	64.7 (51.4–74.3)	35.6–83.0
European	35	903	50.0 (28.4–51.2)	49.9 (39.2–60.6)	344.3	<0.001	90.1 (87.3–92.3)	1.9–98.1
Western Pacific	24	610	37.4 (29.4–50.0)	42.3 (33.9–50.8)	58.8	<0.001	60.9 (39.0–74.9)	11.9–75.7
**Country**								
Australia	15	388	37.3 (26.4–50.0)	44.5 (31.2–58.2)	49.1	<0.001	71.5 (51.9–83.1)	4.2–89.3
Canada	2	59	46.0 (NA)	40.7 (28.1–54.2)	NA		NA	NA
Denmark	3	62	50.0 (25.9–53.6)	23.2 (0.0–78.1)	15.1	0.001	86.7 (61.9–95.4)	0.0–100.0
Finland	1	13	NA	92.3 (64.0–99.8)	NA		NA	NA
France	1	8	NA	50.0 (15.7–84.3)	NA		NA	NA
Germany	1	24	NA	25.0 (9.8–46.7)	NA		NA	NA
Israel	4	110	45.1 (20.0–75.0)	45.2 (3.1–91.5)	27.1	<0.001	88.9 (74.3–95.2)	0.0–100.0
Japan	8	206	38.8 (34.5–47.8)	36.9 (30.1–43.9)	9.8	0.203	28.3 (0.0–67.8)	28.4–45.7
Netherlands	7	157	25.0 (16.4–39.1)	25.0 (12.1–40.3)	23.1	0.001	74.0 (44.5–87.8)	0.0–75.0
Republic of Korea	1	16	NA	37.5 (15.2–64.6)	NA		NA	NA
Sweden	6	201	77.8 (75.7–79.9)	78.7 (72.5–84.3)	3.5	0.632	0.0 (0.0–74.6)	69.7–86.5
Switzerland	1	6	NA	33.3 (4.3–77.7)	NA		NA	NA
UK	11	322	51.6 (46.7-62.8)	56.0 (49.3–62.6)	14.1	0.167	29.3 (0.0–65.2)	42.5–69.1
USA	42	1772	60.0 (52.7–70.7)	61.3 (56.6–65.8)	111.9	<0.001	63.6 (49.0–73.7)	37.2–82.9
**Year of data collection category¶**								
<1995	38	1211	67.9 (54.7–77.1)	65.9 (59.6–72.0)	124.9	<0.001	70.4 (58.9–78.7)	32.7–92.8
1995–2004	21	596	55.3 (37.5–67.9)	55.5 (44.3–66.5)	104.0	<0.001	80.8 (71.5–87.0)	10.6–95.8
≥2005	44	1537	46.2 (27.1–52.7)	41.1 (34.5–47.9)	252.2	<0.001	82.9 (77.8–86.9)	8.1–78.8
**Year of publication category**								
<2000	28	880	69.1 (56.5–77.08)	68.7 (62.1–74.9)	63.8	<0.001	57.7 (35.6–72.2)	40.3–91.7
2000–2009	26	667	57.2 (38.5–72.2)	56.8 (47.1–66.3)	131.0	<0.001	80.9 (72.8–86.6)	13.7–94.8
≥2010	49	1797	46.3 (27.8–55.2)	42.9 (36.5–49.4)	294.3	<0.001	83.7 (79.2–87.2)	8.5–81.4
**All studies**	103	3344	53.8 (37.5–69.1)	53.2 (48.4–58.1)	636.4	<0.001	84.0 (81.1–86.4)	13.4–90.9

CI – confidence interval, HSV-2 – herpes simplex virus type 2, IQR – interquartile range, MD – median, NA – not applicable, nHSV-2 – neonatal herpes attributed to herpes simplex virus type 2, WHO – World Health Organization, x̄ – mean

*A minimum of three measures was required to perform a random-effects meta-analysis. When only two measures were available, the arithmetic mean and its corresponding 95% CI were calculated. If only one study was available, the point estimate was reported with its 95% CI.

†Q – the Cochran’s Q statistic is a measure assessing the existence of heterogeneity in pooled outcome measures, here the proportion of neonatal herpes cases attributed to HSV-2.

‡*I*^2^ – a measure that assesses the magnitude of between-study variation that is due to true differences in the proportion of neonatal herpes cases attributed to HSV-2 across studies rather than chance.

§Prediction interval is a measure that estimates the distribution (95% interval) of true proportion of neonatal herpes cases attributed to HSV-2 around the estimated mean.

¶The categories were determined based on the median time between the year of publication and the year of data collection, which was approximately six years. This interval was rounded to five years to have a five-year bracket.

The Western Pacific Region had the highest pooled mean proportion of nHSV-1 at 57.7% (95% CI = 49.2–66.1), followed by the European Region at 50.4% (95% CI = 39.6–61.2) and the Region of the Americas at 39.6% (95% CI = 35.0–44.3) (**Table 3**).

For nHSV-2, the Region of the Americas had the highest pooled mean proportion at 60.5% (95% CI = 55.8–65.1), followed by the European Region at 49.9% (95% CI = 39.2–60.6) and the Western Pacific Region at 42.3% (95% CI = 33.9–50.8) (**Table 4**). While pooled estimates varied across countries, some had too few studies, limiting the representativeness of these estimates.

Globally, the pooled mean proportions of nHSV-1 and nHSV-2, based on all reported measures, were estimated at 46.9% (95% CI = 42.0–51.8) and 53.2% (95% CI = 48.4–58.1), respectively. When regional pooled estimates were weighted by regional population sizes, the global pooled mean proportions were estimated at 47.3% (95% CI = 39.5–55.0) for nHSV-1 and 52.8% (95% CI = 45.2–60.5) for nHSV-2.

Significant heterogeneity was observed in most meta-analyses (*P* < 0.1), driven by true variations in proportions rather than random chance, as indicated by *I^2^* values exceeding 50% (**Table 3**, **Table 4**). This conclusion was further supported by the wide prediction intervals. Forest plots of the meta-analyses are presented in Figures S8–9 in the **Online Supplementary Document**.

### Predictors of nHSV-1 and nHSV-2 proportions and sources of between-study heterogeneity

The multivariable models explained over 45% of the variation in the proportions of nHSV-1 and nHSV-2. The results indicated a gradual replacement of nHSV-2 by nHSV-1 over time. The proportion of nHSV-1 increased by 1.01-fold per year (95% CI = 1.01–1.02) (**Table 5**), corresponding to an annual rise of 1.4% (95% CI = 0.9–1.9), while the proportion of nHSV-2 decreased by 0.99-fold per year (95% CI = 0.98–0.99) (**Table 6**), reflecting an annual decline of 1.1% (95% CI = 0.6–1.6).

**Table 5:** Univariable and multivariable meta-regression analyses for the proportion of neonatal herpes attributed to HSV-1

Characteristics	Outcome measures, n	Sample size, n	Univariable analyses				Multivariable analyses			
			**PR (95% CI)**	***P*-value**	**LR test *P*-value**	Adjusted R^2^*	**Model 1, APR (95% CI)**	***P*-value**	**Model 2, APR (95% CI)**	***P*-value**
**WHO Region**										
Region of the Americas	44	1831	ref.		<0.001	26.5	ref.		ref.	
European	35	903	1.29 (1.08–1.55)	0.006			1.25 (1.07–1.47)	0.006	1.22 (1.04–1.43)	0.014
Western Pacific	24	610	1.48 (1.21–1.81)	<0.001			1.40 (1.17–1.67)	<0.001	1.35 (1.14–1.62)	0.001
**Country†**										
USA	42	1772	ref.		<0.001	71.4	NA		NA	
Australia	15	388	1.54 (1.28–1.84)	<0.001			NA		NA	
Denmark	3	62	2.01 (1.41–2.87)	<0.001			NA		NA	
Israel	4	110	1.93 (1.42–2.62)	<0.001			NA		NA	
Japan	8	206	1.48 (1.20–1.82)	<0.001			NA		NA	
Netherlands	7	157	1.80 (1.47–2.20)	<0.001			NA		NA	
Sweden	6	201	0.53 (0.38–0.74)	<0.001			NA		NA	
UK	11	322	1.10 (0.90–1.35)	0.341			NA		NA	
Mixed countries‡	7	126	1.50 (1.17–1.91)	0.002			NA		NA	
**Criteria for defining the neonatal period**										
0–29 d	49	1732	ref.		0.988	0.0	NA		NA	
0–90 d	30	1133	0.99 (0.81–1.21)	0.932			NA		NA	
Unclear	24	479	0.98 (0.78–1.24)	0.881			NA		NA	
**Risk of bias: case definition criteria domain**										
Low risk of bias	79	2865	ref.		0.900	0.0	NA		NA	
High risk of bias	24	479	0.99 (0.80–1.22)	0.900			NA		NA	
**Risk of bias: sample size domain**										
Low risk of bias	96	3272	ref.		0.255	0.0	ref.		ref.	
High risk of bias	7	72	0.75 (0.46–1.23)§	0.255			0.83 (0.54–1.28)	0.393	0.81 (0.53–1.24)	0.334
**Year of data collection category**										
<1995	38	1211	ref.		<0.001	24.3	ref.		NA	
1995–2004	21	596	1.36 (1.10–1.69)	0.006			1.29 (1.06–1.59)	0.013	NA	
≥2005	44	1537	1.58 (1.33–1.88)	<0.001			1.50 (1.28–1.76)	<0.001	NA	
**Year of data collection**	103	3344	1.02 (1.01–1.02)	<0.001	<0.001	28.6	NA		1.01 (1.01–1.02)	<0.001

APR – adjusted proportion ratio, CI – confidence interval, LR – likelihood ratio, NA – not applicable, PR – proportion ratio, ref – reference,WHO – World Health Organization.

*Adjusted *R^2^* is a goodness-of-fit statistic indicating the proportion of variance explained by the model after accounting for the number of predictors. Adjusted *R^2^* in the final multivariable model 1 is 45.49%. The adjusted *R^2^* for the final multivariable model 2 is 45.83%.

†The country variable was excluded from the multivariable model due to collinearity with the region variable.

‡This category includes countries with two or fewer studies, including Canada, Finland, France, Germany, Switzerland, and the Republic of Korea.

§Variables with a *P*-value >0.2 were excluded from the multivariable model. However, because this variable was eligible for inclusion in the multivariable meta-regression models for the proportion of neonatal herpes attributed to HSV-2, it was also retained in the HSV-1 models to ensure consistency across analyses.

**Table 6:** Univariable and multivariable meta-regression analyses for the proportion of neonatal herpes attributed to HSV-2

Characteristics	Outcome measures, n	Sample size, n	Univariable analyses				Multivariable analyses			
			**PR (95% CI)**	***P*-value**	**LR test *P*-value**	Adjusted R^2^*	**Model 1, APR (95% CI)**	***P*-value**	**Model 2, APR (95% CI)**	***P*-value**
**WHO Region**										
Region of the Americas	44	1831	ref.		0.040	4.1	ref.		ref.	
European	35	903	0.90 (0.74–1.08)	0.254			0.91 (0.77–1.08)	0.284	0.94 (0.80–1.11)	0.468
Western Pacific	24	610	0.75 (0.60–0.94)	0.012			0.78 (0.64–0.95)	0.015	0.80 (0.66–0.98)	0.029
**Country†**										
USA	42	1772	ref.		0.002	15.0	NA		NA	
Australia	15	388	0.79 (0.61–1.02)	0.067			NA		NA	
Denmark	3	62	0.60 (0.28–1.28)	0.184			NA		NA	
Israel	4	110	0.75 (0.48–1.16)	0.196			NA		NA	
Japan	8	206	0.69 (0.51–0.95)	0.022			NA		NA	
Netherlands	7	157	0.50 (0.34–0.74)	0.001			NA		NA	
Sweden	6	201	1.27 (0.95–1.70)	0.099			NA		NA	
UK	11	322	0.93 (0.72–1.19)	0.565			NA		NA	
Mixed countries‡	7	126	0.79 (0.56–1.12)	0.182			NA		NA	
**Criteria for defining the neonatal period**										
0–29 d	49	1732	ref.		0.790	0.0	NA		NA	
0–90 d	30	1133	0.97 (0.79–1.19)	0.755			NA		NA	
Unclear	24	479	1.05 (0.85–1.31)	0.645			NA		NA	
**Risk of bias: case definition criteria domain**										
Low risk of bias	79	2865	ref.		0.540	0.0	NA		NA	
High risk of bias	24	479	1.06 (0.87–1.30)	0.540			NA		NA	
**Risk of bias: sample size domain**										
Low risk of bias	96	3272	ref.		0.038	7.1	ref.		ref.	
High risk of bias	7	72	1.40 (1.02–1.94)	0.038			1.40 (1.05–1.86)	0.022	1.41 (1.07–1.86)	0.016
**Year of data collection category**										
<1995	38	1211	ref.		<0.001	22.7	ref.		NA	
1995–2004	21	596	0.87 (0.71–1.06)	0.159			0.87 (0.72–1.06)	0.160	NA	
≥2005	44	1537	0.69 (0.58–0.82)	<0.001			0.71 (0.60–0.84)	<0.001	NA	
**Year of data collection**	103	3344	0.99 (0.98–0.99)	<0.001	<0.001	27.1	NA		0.99 (0.98–0.99)	<0.001

APR – adjusted proportion ratio, CI – confidence interval, LR – likelihood ratio, NA – not applicable; PR – proportion ratio, ref – reference, WHO – World Health Organization

*Adjusted *R^2^* is a goodness-of-fit statistic indicating the proportion of variance explained by the model after accounting for the number of predictors. Adjusted *R^2^* in the final multivariable model 1 = 45.49%. Adjusted *R^2^* in the final multivariable model 2 = 45.83%.

†The country variable was excluded from the multivariable model due to collinearity with the region variable.

‡This category includes countries with two or fewer studies, including Canada, Finland, France, Germany, Switzerland, and the Republic of Korea.

Compared to the Region of the Americas, the European Region had a 1.22-fold (95% CI = 1.04–1.43) higher proportion of nHSV-1, while the Western Pacific Region had a 1.35-fold (95% CI = 1.14–1.62) higher proportion of nHSV-1 (**Table 5**). Conversely, compared to the Region of the Americas, the European Region had a 0.94-fold (95% CI = 0.80–1.11) lower proportion of nHSV-2, while the Western Pacific Region had a 0.80-fold (95% CI = 0.66–0.98) lower proportion of nHSV-2 (**Table 6**).

Regarding the influence of study methods on observed proportions, there was no evidence that variations in the criteria for defining the neonatal period or in the ROB classification for the case definition criteria domain affected the observed proportions of nHSV-1 or nHSV-2. However, only for nHSV-2 proportions was there evidence that studies classified as high ROB in the sample size domain reported higher proportions (**Table 6**).

Sensitivity analyses on the meta-regression models for nHSV-1 and nHSV-2 proportions, restricted to studies with reported data collection years and excluding those with imputed years, yielded results identical to the main analysis, showing a 1.01-fold per year increase in the proportion of nHSV-1 (95% CI = 1.01–1.02) and a 0.99-fold per year decrease in the proportion of nHSV-2 (95% CI = 0.98–0.99), thereby confirming the observed temporal trends.

## DISCUSSION

The findings indicate that the global incidence rate of nHSV, based on the best available estimate from the three reporting regions, is approximately eight per 100 000 live births, corresponding to roughly one affected newborn per 10 000 live births with this severe condition. However, this global estimate conceals substantial regional variations. The incidence rate in the Region of the Americas was approximately 13 per 100 000 live births, more than twice the rates observed in the European and Western Pacific regions, at five and three per 100 000 live births, respectively. Importantly, the study identified a rising trend in the nHSV incidence rate, increasing by more than 3% annually, underscoring the growing public health urgency to address nHSV.

The results indicated that, globally, nHSV cases are nearly equally attributed to HSV-1 and HSV-2 infections, though their relative contributions vary by region. HSV-1 accounted for the highest proportion of cases in the Western Pacific Region (58%), followed by the European Region (50%) and the Region of the Americas (40%). Conversely, HSV-2 accounted for the highest proportion of cases in the Region of the Americas (61%), followed by the European Region (50%) and the Western Pacific Region (42%).

Notably, we identified a shifting epidemiologic pattern, with the contribution of HSV-1 increasing by 1% per year and that of HSV-2 declining at a similar rate. The implications of this shift for the clinical severity of nHSV remain uncertain and would be best examined through a dedicated investigation of temporal changes in disease severity. Existing evidence indicates that nHSV-2 is more strongly associated with CNS and disseminated disease [11], which are linked to higher morbidity and mortality than the SEM form [1–3]. As such, an increasing overall incidence rate of nHSV accompanied by a shift in etiologic types may have more nuanced implications for disease burden than incidence rate trends alone.

The study’s empirical findings require explanation, as they may reflect underlying shifts in the epidemiology of HSV infections or the influence of additional factors, such as greater temporal overlap between infection and pregnancy. Further work, such as dedicated mathematical modelling studies, is needed to disentangle and quantify the relative contributions of these different mechanistic pathways.

The observed patterns may, for example, be linked to documented changes in the epidemiology of HSV-1 and HSV-2 infections [15–26]. In regions where nHSV data are available, HSV-1 has increasingly been acquired genitally rather than orally [20–26], while HSV-2 prevalence has shown a slow, gradual decline over time [15–19]. Genital HSV-1 infections have been reported to increase by 1–4% annually in these regions [20–24], consistent with the 3.5% annual increase in nHSV incidence rate observed in the present study. Moreover, evidence suggesting higher transmissibility of HSV-1 to neonates compared with HSV-2 may further contribute to the observed trends [61].

Interestingly, while a declining prevalence of HSV-2 might be expected to reduce the incidence of nHSV, this may not necessarily occur. A paradoxical effect could emerge – as HSV-2 prevalence declines, the basic reproduction number decreases, leading to an increase in the average age at infection [18,62]. This shift could result in greater overlap between primary genital herpes, when the virus is most likely to be transmitted to neonates [1–3,6] and peak pregnancy age, thereby potentially increasing the incidence of nHSV.

The pooled global estimate of the nHSV incidence rate, 8.2 per 100 000 live births, aligns with a mathematical modelling estimate of 10.3 per 100 000 live births [5]. However, both likely underestimate the true incidence rate. Diagnosing nHSV is inherently challenging due to its diverse clinical manifestations, overlap with other neonatal conditions, and the need for testing multiple anatomical sites (*e.g.* CSF, blood, vesicles) [1,2,33]. Laboratory confirmation is also complex and resource-intensive [1,2,33]. The relative rarity of nHSV further contributes to underdiagnosis or misdiagnosis by clinicians [63].

Reporting of nHSV cases is not mandatory in most countries [31]. Even in high-income settings such as the USA, despite the severe consequences of the disease, nHSV is not a nationally notifiable condition [32]. As of 2023, only six states require case notification, leaving 44 states without formal mechanisms to monitor the disease burden [32].

These challenges in diagnosing and documenting nHSV hinder the development of effective surveillance systems, particularly in low- and middle-income countries, as evidenced by the absence of data from three WHO regions. Active surveillance for nHSV is implemented in only a few countries worldwide [64-69]. Establishing a standardised case definition and designating nHSV as a nationally notifiable condition are essential steps to enhance case detection and strengthen surveillance systems [32].

Published evidence and this review support the underdiagnosis and underestimation of nHSV incidence rate. Most analysed data were derived from surveillance or administrative records, which, as demonstrated in an Australian analysis, may capture fewer than half of actual cases [70]. Retrospective case reviews are particularly prone to underreporting and often skewed toward more severe cases, partly due to the absence of a standardised diagnostic code for nHSV [5].

Further supporting this underestimation, we found that data from clinical settings or health care databases reported substantially higher incidence rates than those from national surveillance registries or reports (**Table 2**). This underestimation is also reinforced by findings from prospective cohort studies of nHSV acquisition, which, although seldom conducted, tend to report higher incidence rates [71].

Our findings highlight the need for expanded diagnostic availability in low- and middle-income settings and for strengthened surveillance systems that integrate laboratory and epidemiological data to improve global case detection. Increasing global awareness of nHSV is equally vital, particularly in low- and middle-income settings where diagnostic challenges persist [5,70]. Furthermore, well-designed prospective cohort studies that collect primary data on nHSV are essential for improving the understanding, prevention, and management of this condition [5,71].

The estimates in this study are based on data from only three WHO regions, with no available data from the African Region, Eastern Mediterranean Region, or South-East Asia Region, where nHSV is difficult to diagnose and is likely substantially under-ascertained [31,63]. The African Region is expected to have a disproportionately higher nHSV incidence rate, reflecting the high prevalence of maternal HSV-2 infection [14] and the substantial burden of HIV [72]. HIV increases HSV-2 genital shedding at delivery, thereby elevating the risk of neonatal transmission [5,73]. In addition, lower caesarean section rates in the African Region and other low- and middle-income countries, in the presence of genital lesions, may further contribute to increased nHSV incidence rate [5,71]. However, given the widespread acquisition of HSV-1 in childhood in this region [45] – and consequently lower genital acquisition in adulthood [20–26] – a relatively small proportion of nHSV cases is expected to be attributable to HSV-1, with the majority due to HSV-2. In contrast, in the Eastern Mediterranean Region and South-East Asia Region, maternal HSV-2 prevalence is relatively lower [16,74], and childhood HSV-1 prevalence is also lower [45,75,76], resulting in a greater proportion of genital HSV-1 acquisition occurring in adulthood [20–26]. These patterns may lead to a lower overall nHSV incidence rate but a greater relative contribution of nHSV-1 compared with nHSV-2. Nevertheless, because HSV-1 prevalence in these regions remains higher than in developed countries [75,76], the absolute incidence rate of nHSV-1 is still likely to be substantially lower than that observed in developed settings.

The observed and rising incidence rate of nHSV underscores the urgent need to expand advancements in nHSV management, such as timely diagnosis and improved clinical outcomes achieved in high-income countries [11], to low- and middle-income settings. These findings also emphasise the importance of developing HSV vaccines, both prophylactic and therapeutic, alongside advancements in microbicides, diagnostic tools, and treatment options [5,77,78]. In particular, a vaccine has the potential to serve as a cornerstone strategy for reducing the burden of nHSV by preventing the underlying maternal infections responsible for neonatal transmission [77,79,80].

This study has limitations and considerations. Data were available from only three of the six WHO regions, with certain countries contributing disproportionately larger data sets, including the USA in the Region of the Americas, the UK and the Netherlands in the European Region, and Australia in the Western Pacific Region.

The review was restricted to PubMed and Embase, the two principal international biomedical databases. While additional databases (*e.g.* Web of Science and Scopus) and regional repositories could have been included, they largely index the same core literature and are unlikely to provide substantial added value for a rare, underdiagnosed condition like nHSV, which is primarily documented in high-resource settings. The dual-pronged search approach implemented in this study – combining comprehensive database searches with targeted national surveillance data – captured both published and programmatic sources. In light of these considerations, it is unlikely that relevant or representative data were omitted in a manner that materially affected the study's findings or conclusions.

The ROB assessment in this study was conducted using a specific approach, the Joanna Briggs Institute critical appraisal tool [36,37], supplemented by guidance from the Cochrane approach [27]. This combination represents the current state-of-the-art methodology for systematic reviews of proportion measures and was therefore adopted for this analysis. To our knowledge, no alternative applicable risk-of-bias tools have been specifically developed for rare-disease surveillance studies with characteristics comparable to those of nHSV.

Evidence of publication bias was detected, but only for incidence rate measures. Sensitivity analysis suggested that the observed bias was primarily driven by regional and country-level differences in the number of live births and nHSV incidence rates, rather than true publication bias (Figure S5 and Table S5 in the **Online Supplementary Document**). A small number of outlying values may also have contributed to the observed asymmetry (Figure S5 in the **Online Supplementary Document**).

Methods for nHSV ascertainment and confirmation varied across studies, utilising different combinations of clinical and laboratory approaches. Some of these methods tended to be biased toward more severe cases, potentially leading to underestimates of incidence rates and of the relative contribution of nHSV-1 *vs*. nHSV-2. Studies with unclear case-ascertainment methods tended to report higher incidence rates than those based on laboratory-confirmed cases (**Table 2**), raising concern about potential case misclassification.

Most data on nHSV were derived from large-scale, quality surveillance platforms in high-resource settings. These platforms are among the most reliable global sources for identifying nHSV, offering wide geographic coverage and extended observation periods. However, within these systems, case definitions, diagnostic practices, and laboratory capacities may vary over time and across regions. Additionally, surveillance reports developed for public health or programmatic purposes may not consistently include detailed methodological documentation, even when high diagnostic standards have been applied. As a result, some data points in our review were conservatively labelled as ‘unclear’ for diagnostic confirmation solely because the exact methods were not explicitly reported, not due to concerns about data validity or rigour.

It was not possible to adjust pooled estimates for differences in specific laboratory diagnostic methods or include diagnostic methods as covariates in the meta-regression analyses. Surveillance studies often involve multiple laboratories using different, but guideline-consistent, diagnostic approaches. These surveillance platforms span extended time periods during which diagnostic practices may also evolve, including increased adoption of PCR. Comparative data on the sensitivity of PCR *vs*. culture for detecting HSV from neonatal surface swabs remain limited due to the rarity of nHSV, which hinders statistically powered comparisons [2,81]. Sensitivity may also vary by sample type (*e.g.* vesicle, CSF, blood) [2]. Nonetheless, current evidence indicates that PCR is more sensitive than culture and is now considered the gold standard for confirming nHSV [81].

Unexpectedly elevated nHSV incidence rates were reported in certain geographic settings, suggesting the possibility of isolated outbreaks, such as in neonatal wards [82], specific cultural practices, such as ritual circumcision [83], or methodological heterogeneity or inconsistency in the definition and ascertainment of nHSV cases, such as with respect to the definition of the neonatal period [84].

The definition of the neonatal period is a critical determinant of nHSV case capture and was therefore explicitly incorporated into both the eligibility criteria and the meta-regression analyses. A narrow definition of the neonatal period (zero to 29 days) may fail to capture cases in which clinical diagnosis occurs after 29 days, despite infection having been acquired intrapartum or shortly thereafter. Conversely, an extended definition (zero to 90 days) may include infections acquired after the neonatal period (beyond 29 days of life), which may reflect horizontal rather than vertical transmission and thus should not be classified as nHSV. Accordingly, studies that defined nHSV outside the standard diagnostic window of zero to 90 days were excluded, as a proportion of identified cases in such studies could plausibly represent post-neonatal horizontal transmission.

Despite these conceptual considerations, variation in the definition of the neonatal period was not found to influence incidence rate estimates or HSV-type proportions in the meta-regression analyses (**Table 2**, **Table 5**, **Table 6**). Importantly, only studies meeting predefined, rigorous inclusion criteria were retained in this systematic review, ensuring consistency in case definitions and strengthening the validity and interpretability of the study findings.

The study applied specific inclusion criteria to ensure that valid and comparable data were incorporated into the evidence base, including those that excluded studies that did not test multiple anatomical sites. This approach reflects the inherent diagnostic challenges of nHSV, given its heterogeneous clinical manifestations and overlap with other, more common neonatal conditions [1,2,33]. Rigorous diagnostic criteria are therefore essential to distinguish true nHSV cases from other neonatal illnesses.

Notably, these criteria were developed in alignment with the available evidence base and with the standards used by existing surveillance systems, and they are consistent with guidelines applied across countries and time periods. They were not intended to, nor do they systematically, exclude data from low-resource settings. The absence of data from such settings reflects the lack of surveillance systems capable of capturing this rare condition, rather than exclusion driven by the study's eligibility criteria.

Given the rarity of nHSV and the extensive catchment areas covered by individual studies, some studies overlapped in terms of time, location, or population. As a result, certain measures were not entirely independent, and it was not possible to disentangle these overlaps with the available data. Additionally, due to the rarity of nHSV, many studies that performed HSV typing relied on relatively small sample sizes, which were occasionally skewed toward specific disease forms, potentially biasing the reported proportions of nHSV-1 *vs*. nHSV-2.

In a small number of studies, the number of live births was imputed using the median value from other studies within the same geographic setting; as such, these estimates may not be exact and could, in principle, influence variance estimates and study weights, introducing some uncertainty and potential circularity. However, this applied to only seven of 140 measures (5.0%) and is therefore not likely to materially affect the overall results. This was corroborated by the sensitivity analysis excluding these measures, which yielded results consistent with the main analysis.

Dual HSV-1/HSV-2–positive neonatal infections were included in both the nHSV-1 and nHSV-2 categories; however, this applied to only a very small proportion of studies (n = 2, 1.9%), and in those studies, only a few specimens were included relative to the overall sample size. Consistent with this, the median proportion of nHSV-1 among nHSV cases was 46.2%, whereas that of nHSV-2 was 53.8%, totalling 100%, underscoring the rarity of dual-positive infections. Given their very low frequency, separate categorisation of dual-positive cases or restriction of etiologic proportions to mutually exclusive categories was not warranted.

Heterogeneity in incidence rates and the proportions of nHSV-1 *vs*. nHSV-2 was observed; however, the meta-regression analyses explained a large proportion of this variability, attributing it to differences in epidemiological factors such as region and year, as well as study methodologies such as site type (**Tables 2**, **Table 5**, **Table 6**).

This study has strengths. It is the first global systematic review, meta-analysis, and meta-regression analysis of nHSV, a rare but highly morbid and potentially fatal condition. A comprehensive search strategy with no time or language restrictions was employed, ensuring broad and inclusive data collection. Rigorous study quality assessments were conducted, and the overall high quality of the included studies enhances the reliability and robustness of the findings. The meta-regression analyses yielded insights into the role of key epidemiological and methodological factors, helping to explain the observed heterogeneity in nHSV incidence rates and proportions. Finally, extensive sensitivity analyses were conducted across the different analytical components of this study to evaluate the potential impact of the limitations discussed above; these analyses yielded results consistent with the corresponding main analyses, thereby reinforcing the robustness of the study's conclusions.

## CONCLUSIONS

The global incidence rate of nHSV is estimated at approximately one case per 10 000 live births, with an annual increase of over 3%. While HSV-1 and HSV-2 contribute nearly equally to cases worldwide, incidence rates and etiological patterns vary by region. The proportion of nHSV-1 cases is rising by 1% annually, while that of nHSV-2 is declining at the same rate, reflecting shifts in HSV epidemiology, particularly the increasing incidence of genital HSV-1. Addressing this burden requires expanding management strategies in low- and middle-income settings while advancing HSV vaccines, diagnostics, and treatments to reduce the global impact of nHSV.

## Additional material


Online Supplementary Document
