# Supplementary material for: Global epidemiology of neonatal herpes: systematic review, meta-analyses, and meta-regressions
Source: J Glob Health. 2026 Mar 20;16:04104. doi: 10.7189/jogh.16.04104 (PMC13003892; doi:10.7189/jogh.16.04104)
Supplement: Online Supplementary Document [file jogh-16-04104-s001.pdf]

Supplement to: Aldien AS, Harfouche M, Alareeki A, Chemaitelly H, Abu-Raddad LJ. Global epidemiology of neonatal herpes: systematic review, meta-analyses, and meta-regressions. J Glob Health. 2026;16:04104.

Table of Contents

Table S1. PRISMA checklist. Preferred Reporting Items for Systematic Reviews and Meta-analyses (PRISMA) checklist [1,2]. 3

Box S1. Data sources and search criteria for the systematic review of neonatal herpes epidemiology. 5

Box S2. Countries and their corresponding World Health Organization regional classifications [3]. 6

Box S3. Variables extracted from the publications that met the inclusion criteria. 7

Table S2. Risk of bias assessment. Description of the risk of bias assessment for publications that met the inclusion criteria, conducted using the Joanna Briggs Institute critical appraisal tool [4-6]. 8

Box S4. Variables included in meta-regression analyses. Factors (variables) chosen a priori and integrated into both the univariable and multivariable meta-regression analyses in this study. 10

Table S3. Studies reporting neonatal herpes incidence rates, categorized by World Health Organization region. 11

Table S4. Studies reporting neonatal herpes proportions attributed to HSV-1 versus HSV-2, categorized by World Health Organization region. 15

Figure S1. Risk of bias assessment of individual incidence rate studies. Traffic light plot of the risk of bias assessment for individual studies reporting incidence rates of neonatal herpes, based on the Joanna Briggs Institute's critical appraisal tool [4-6]. 18

Figure S2. Risk of bias assessment of individual HSV-1 versus HSV-2 studies. Traffic light plot of the risk of bias assessment for individual studies reporting neonatal herpes proportions attributed to HSV-1 versus HSV-2, based on the Joanna Briggs Institute's critical appraisal tool [4-6]. 22

Figure S3. Summary of risk of bias assessment of incidence rate studies. Summary plot of the risk of bias assessment of individual studies reporting incidence rate of neonatal herpes, based on the Joanna Briggs Institute's critical appraisal tool [4-6]. 25

Figure S4. Summary of risk of bias assessment of HSV-1 versus HSV-2 studies. Summary plot of the risk of bias assessment of individual studies reporting neonatal herpes proportions attributed to HSV-1 versus HSV-2, based on the Joanna Briggs Institute's critical appraisal tool [4-6]. 26

Table S5. Assessment of publication bias using Doi plots and the LFK index. 27

Figure S5. Doi plots for assessing publication bias. Doi plot of studies reporting neonatal herpes A) incidence rate, B) cases attributed to HSV-1, and C) cases attributed to HSV-2. 28

Figure S6. Forest plot for neonatal herpes incidence rate. Forest plot illustrating the global and regional pooled mean incidence rates of neonatal herpes. 29

Table S6. Leave-one-out sensitivity analysis. Leave-one-out analysis of pooled mean estimates for the incidence rate of neonatal herpes. 31

Table S7. Sensitivity analyses on the meta-regressions. Sensitivity analyses on the main meta-regression analyses for the incidence rate of neonatal herpes. 34

Figure S7. Forest plot of HSV-1 contribution to neonatal herpes. Forest plot illustrating the global and regional pooled mean proportion of neonatal herpes cases attributed to HSV-1. 35

Figure S8. Forest plot of HSV-2 contribution to neonatal herpes. Forest plot illustrating the global and regional pooled mean proportion of neonatal herpes cases attributed to HSV-2. 38

References 41

Table S1. PRISMA checklist. Preferred Reporting Items for Systematic Reviews and Meta-analyses (PRISMA) checklist [1,2].

Section and topic	Item #	Checklist item	Location where item is reported
**Title**			
Title	1	Identify the report as a systematic review.	Title
**Abstract**			
Abstract	2	See the PRISMA 2020 for Abstracts checklist (table 2).	Abstract
**Introduction**			
Rationale	3	Describe the rationale for the review in the context of existing knowledge.	Introduction
Objectives	4	Provide an explicit statement of the objective(s) or question(s) the review addresses.	Introduction
**Methods**			
Eligibility criteria	5	Specify the inclusion and exclusion criteria for the review and how studies were grouped for the syntheses.	Methods: Study selection process and inclusion and exclusion criteria
Information sources	6	Specify all databases, registers, websites, organisations, reference lists and other sources searched or consulted to identify studies. Specify the date when each source was last searched or consulted.	Methods: Data sources and search strategy; Box S1; Figure 1
Search strategy	7	Present the full search strategies for all databases, registers and websites, including any filters and limits used.	Box S1
Selection process	8	Specify the methods used to decide whether a study met the inclusion criteria of the review, including how many reviewers screened each record and each report retrieved, whether they worked independently, and if applicable, details of automation tools used in the process.	Methods: Study selection process and inclusion and exclusion criteria
Data collection process	9	Specify the methods used to collect data from reports, including how many reviewers collected data from each report, whether they worked independently, any processes for obtaining or confirming data from study investigators, and if applicable, details of automation tools used in the process.	Methods: Data extraction; Boxes S2 and S3
Data items	10a	List and define all outcomes for which data were sought. Specify whether all results that were compatible with each outcome domain in each study were sought (e.g., for all measures, time points, analyses), and if not, the methods used to decide which results to collect.	Methods: Data extraction; Boxes S2 and S3
	10b	List and define all other variables for which data were sought (e.g., participant and intervention characteristics, funding sources). Describe any assumptions made about any missing or unclear information.	Methods: Critical appraisal of studies; Assessment of publication bias; Table S2;
Study risk of bias assessment	11	Specify the methods used to assess risk of bias in the included studies, including details of the tool(s) used, how many reviewers assessed each study and whether they worked independently, and if applicable, details of automation tools used in the process.	Methods: Critical appraisal of studies; Assessment of publication bias; Table S2
Effect measures	12	Specify for each outcome the effect measure(s) (e.g. risk ratio, mean difference) used in the synthesis or presentation of results.	Methods: Data extraction; Meta-analyses; Meta-regressions; Box S4
Synthesis methods	13a	Describe the processes used to decide which studies were eligible for each synthesis (e.g. tabulating the study intervention characteristics and comparing against the planned groups for each synthesis (item #5)).	Methods: Meta-analyses; Meta-regressions; Box S4
	13b	Describe any methods required to prepare the data for presentation or synthesis, such as handling of missing summary statistics, or data conversions.	Methods: Meta-analyses; Meta-regressions; Box S4
	13c	Describe any methods used to tabulate or visually display results of individual studies and syntheses.	Methods: Meta-analyses; Critical appraisal of studies; Assessment of publication bias
	13d	Describe any methods used to synthesise results and provide a rationale for the choice(s). If meta-analysis was performed, describe the model(s), method(s) to identify the presence and extent of statistical heterogeneity, and software package(s) used.	Methods: Meta-analyses
	13e	Describe any methods used to explore possible causes of heterogeneity among study results (e.g. subgroup analysis, meta-regression).	Methods: Meta-regressions; Box S4
	13f	Describe any sensitivity analyses conducted to assess robustness of the synthesised results.	Methods: Meta-regressions; Critical appraisal of studies; Assessment of publication bias; Box S4
Reporting bias assessment	14	Describe any methods used to assess risk of bias due to missing results in a synthesis (arising from reporting biases).	N/A
Certainty assessment	15	Describe any methods used to assess certainty (or confidence) in the body of evidence for an outcome.	Methods: Meta-analyses; Meta-regressions; Box S4
**Results**			
Study selection	16a	Describe the results of the search and selection process, from the number of records identified in the search to the number of studies included in the review, ideally using a flow diagram (see Figure 1).	Results: Search results and scope of evidence; Figure 1; Tables S3 and S4
	16b	Cite studies that might appear to meet the inclusion criteria, but which were excluded, and explain why they were	Figure 1; Tables S3 and S4

		excluded.	
Study characteristics	17	Cite each included study and present its characteristics.	Results: Search results and scope of evidence; Tables S3 and S4
Risk of bias in studies	18	Present assessments of risk of bias for each included study.	Results: Risk of bias assessments and publication bias; Figures S1-S5; Table S5
Results of individual studies	19	For all outcomes, present, for each study: (a) summary statistics for each group (where appropriate) and (b) an effect estimate and its precision (e.g. confidence/credible interval), ideally using structured tables or plots.	Results: Overview and pooled mean estimates of nHSV incidence rate; Overview and pooled mean estimates of nHSV-1 and nHSV-2 proportions; Table 1; Tables 3-4; Figures S6-S8
Results of syntheses	20a	For each synthesis, briefly summarise the characteristics and risk of bias among contributing studies.	Results: Search results and scope of evidence; Risk of bias assessments and publication bias; Figures S1-S5; Table S5
	20b	Present results of all statistical syntheses conducted. If meta-analysis was done, present for each the summary estimate and its precision (e.g. confidence/credible interval) and measures of statistical heterogeneity. If comparing groups, describe the direction of the effect.	Results: Overview and pooled mean estimates of nHSV incidence rate; Overview and pooled mean estimates of nHSV-1 and nHSV-2 proportions; Table 1; Tables 3-4; Figures S6-S8
	20c	Present results of all investigations of possible causes of heterogeneity among study results.	Results: Predictors of nHSV incidence rate and sources of between-study heterogeneity; Predictors of nHSV-1 and nHSV-2 proportions and sources of between-study heterogeneity; Table 2; Tables 5-6; Table S7
	20d	Present results of all sensitivity analyses conducted to assess the robustness of the synthesised results.	Results: Predictors of nHSV incidence rate and sources of between-study heterogeneity; Predictors of nHSV-1 and nHSV-2 proportions and sources of between-study heterogeneity; Table 2; Tables 5-6; Table S7
Reporting biases	21	Present assessments of risk of bias due to missing results (arising from reporting biases) for each synthesis assessed.	N/A
Certainty of evidence	22	Present assessments of certainty (or confidence) in the body of evidence for each outcome assessed.	Results: Overview and pooled mean estimates of nHSV incidence rate; Overview and pooled mean estimates of nHSV-1 and nHSV-2 proportions; Predictors of nHSV incidence rate and sources of between-study heterogeneity; Predictors of nHSV-1 and nHSV-2 proportions and sources of between-study heterogeneity; Tables 1-6; Figures S6-S8
**Discussion**			
Discussion	23a	Provide a general interpretation of the results in the context of other evidence.	Discussion: paragraphs 1-13
	23b	Discuss any limitations of the evidence included in the review.	Discussion: paragraphs 14-23
	23c	Discuss any limitations of the review processes used.	N/A
	23d	Discuss implications of the results for practice, policy, and future research.	Discussion: paragraph 24
**Other information**			
Registration and protocol	24a	Provide registration information for the review, including register name and registration number, or state that the review was not registered.	N/A
	24b	Indicate where the review protocol can be accessed, or state that a protocol was not prepared.	N/A
	24c	Describe and explain any amendments to information provided at registration or in the protocol.	N/A
Support	25	Describe sources of financial or non-financial support for the review, and the role of the funders or sponsors in the review.	Funding; Acknowledgement
Competing interests	26	Declare any competing interests of review authors.	Competing interests
Availability of data, code, and other materials	27	Report which of the following are publicly available and where they can be found: template data collection forms; data extracted from included studies; data used for all analyses; analytic code; any other materials used in the review.	Data availability statement

Abbreviations: nHSV, Neonatal herpes simplex virus; N/A, Not applicable.

Box S1. Data sources and search criteria for the systematic review of neonatal herpes epidemiology.

**PubMed (last searched on December 12, 2024):**
("Infant, Newborn"[MeSH] OR "Neonat*"[Text]) AND (Simplexvirus[MeSH] OR "Herpesvirus"[Text] OR "HSV-2"[Text] OR "HSV"[Text] OR "HSV-1"[Text]) OR (Neonat* herpes[Text] OR "neonatal HSV"[Text] OR "neonatal HSV-1"[Text] OR "neonatal HSV-2"[Text] OR herpes newborn[Text] OR HSV newborn[Text] OR HSV-1 newborn[Text] OR HSV-2 newborn[Text] OR Infant herpes[Text] OR Infant HSV[Text] OR Infant HSV-1[Text] OR Infant HSV-2[Text] OR Perinatal herpes[Text] OR Perinatal HSV[Text] OR Perinatal HSV-1[Text] OR Perinatal HSV-2[Text] OR intrauterine Herpes[Text] OR intrauterine HSV[Text] OR intrauterine HSV-1[Text] OR intrauterine HSV-2[Text] OR congenital HSV[Text] OR congenital HSV-1[Text] OR congenital HSV-2[Text] OR Congenital Herpes[Text] OR neonatal herpetic keratitis[Text] OR keratoconjunctivitis in neonate[Text] OR Neonatal herpes encephalitis[Text] OR disseminated neonatal HSV[Text] OR disseminated neonatal herpes[Text] OR Mother-to-Child Transmission Herpes[Text] OR vertical transmission herpes[Text])
**Embase (last searched on December 12, 2024):**
((exp infant/ or exp newborn/) OR (Neonat*).mp.) AND ((exp herpes/ or exp herpes simplex/ or exp Simplexvirus/) OR (Herpes or Simplexvirus or Herpesvirus or HSV or HSV-1 or HSV-2).mp.) OR ((Neonat* herpes or neonatal HSV or neonatal HSV-1 or neonatal HSV-2 or herpes newborn medor HSV newborn or HSV-1 newborn or HSV-2 newborn or Infant herpes or Infant HSV or Infant HSV-1 or Infant HSV-2 or Perinatal herpes or Perinatal HSV or Perinatal HSV-1 or Perinatal HSV-2 or intrauterine Herpes or intrauterine HSV or intrauterine HSV-1 or intrauterine HSV-2 or congenital HSV or congenital HSV-1 or congenital HSV-2 or Congenital Herpes or neonatal herpetic keratitis or keratoconjunctivitis in neonate or Neonatal herpes encephalitis or disseminated neonatal HSV or disseminated neonatal herpes or Mother-to-Child Transmission Herpes or vertical transmission herpes or (skin, eye, and mouth disease*)).mp.)
**The International Network of Paediatric Surveillance Units (last searched on December 12, 2024)**
Neonatal herpes, neonatal herpes simplex virus, HSV-1, HSV-2.

Abbreviations: HSV-1, Herpes simplex virus type 1; HSV-2, Herpes simplex virus type 2.

Box S2. Countries and their corresponding World Health Organization regional classifications [3].

**African Region**
Algeria, Angola, Benin, Botswana, Burkina Faso, Burundi, Cabo Verde, Cameroon, Central African Republic, Chad, Comoros, Congo, Cote d'Ivoire, Democratic Republic of the Congo, Equatorial Guinea, Eritrea, Eswatini, Ethiopia, Gabon, Gambia, Ghana, Guinea, Guinea-Bissau, Kenya, Lesotho, Liberia, Madagascar, Malawi, Mali, Mauritania, Mauritius, Mozambique, Namibia, Niger, Nigeria, Rwanda, Sao Tome and Principe, Senegal, Seychelles, Sierra Leone, South Africa, South Sudan, Togo, Uganda, United Republic of Tanzania, Zambia, Zimbabwe.
**Eastern Mediterranean Region**
Afghanistan, Bahrain, Djibouti, Egypt, Iran (Islamic Republic of), Iraq, Jordan, Kuwait, Lebanon, Libya, Morocco, Oman, Pakistan, Qatar, Saudi Arabia, Somalia, Sudan, Syrian Arab Republic, Tunisia, United Arab Emirates, Yemen.
**European Region**
Albania, Andorra, Armenia, Austria, Azerbaijan, Belarus, Belgium, Bosnia and Herzegovina, Bulgaria, Croatia, Cyprus, Czechia, Denmark, Estonia, Finland, France, Georgia, Germany, Greece, Hungary, Iceland, Ireland, Israel, Italy, Kazakhstan, Kyrgyzstan, Latvia, Lithuania, Luxembourg, Malta, Monaco, Montenegro, Netherlands (Kingdom of the), North Macedonia, Norway, Poland, Portugal, Republic of Moldova, Romania, Russian Federation, San Marino, Serbia, Slovakia, Slovenia, Spain, Sweden, Switzerland, Tajikistan, Türkiye, Turkmenistan, Ukraine, United Kingdom of Great Britain and Northern Ireland, Uzbekistan.
**Region of the Americas**
Antigua and Barbuda, Argentina, Bahamas, Barbados, Belize, Bolivia (Plurinational State of), Brazil, Canada, Chile, Colombia, Costa Rica, Cuba, Dominica, Dominican Republic, Ecuador, El Salvador, Grenada, Guatemala, Guyana, Haiti, Honduras, Jamaica, Mexico, Nicaragua, Panama, Paraguay, Peru, Saint Kitts and Nevis, Saint Lucia, Saint Vincent and the Grenadines, Suriname, Trinidad and Tobago, United States of America, Uruguay, Venezuela (Bolivarian Republic of).
**South-East Asian Region**
Bangladesh, Bhutan, Democratic People's Republic of Korea, India, Indonesia, Maldives, Myanmar, Nepal, Sri Lanka, Thailand, Timor-Leste.
**Western Pacific Region**
Australia, Brunei Darussalam, Cambodia, China, Cook Islands, Fiji, Japan, Kiribati, Lao People's Democratic Republic, Malaysia, Marshall Islands, Micronesia (Federated States of), Mongolia, Nauru, New Zealand, Niue, Palau, Papua New Guinea, Philippines, Republic of Korea, Samoa, Singapore, Solomon Islands, Tonga, Tuvalu, Vanuatu, Viet Nam.

Box S3. Variables extracted from the publications that met the inclusion criteria.

Study characteristics:

- Author(s)
- Year of publication
- Full citation
- Country
- City
- Study design
- Sampling methodology
- Year(s) of data collection
- Site type (national surveillance registry/report, clinical setting, healthcare database, virological laboratory, mixed/unclear)
- Estimate type (national, regional, or institutional)
- Criteria for defining the neonatal period (0 to 29 days, 0 to 90 days, unclear)
- Method of ascertainment (laboratory confirmation, clinical and/or laboratory confirmation, unclear)
- Laboratory methods used to type nHSV cases

Outcome measures:

- Number of live births
- Number of nHSV cases
- nHSV incidence rate
- Number of typed nHSV cases
- Number and proportion of typed nHSV cases attributed to HSV-1
- Number and proportion of typed nHSV cases attributed to HSV-2

Abbreviations: nHSV, Neonatal herpes simplex virus.

Table S2. Risk of bias assessment. Description of the risk of bias assessment for publications that met the inclusion criteria, conducted using the Joanna Briggs Institute critical appraisal tool [4-6].

Domain	Assessment summary
**1. Sample frame**	
Was the sample frame appropriate to address the target population?	Low ROB. All included studies applied an appropriate sample frame, as they estimated neonatal herpes incidence outcomes among the entire population of live births within clearly defined geographic/catchment areas and over specified time periods.
**2. Participant sampling**	
Were study participants sampled in an appropriate way?	Low ROB. All included studies assessed neonatal herpes incidence outcomes among the entire population of live births within clearly defined geographic/catchment areas and specified time periods.
**3. Sample size**	
**Was the sample size adequate?**	For neonatal herpes incidence rate, ROB was low. All included studies estimated incidence rate among the entire population of live births within clearly defined geographic/catchment areas and specified time periods.<br>For the proportion of neonatal herpes cases attributed to HSV-1 versus HSV-2, studies were classified as low ROB if at least 50% of identified cases underwent laboratory typing for HSV-1 versus HSV-2. Studies with less than 50% of cases typed were considered high ROB.
**4. Subjects & setting**	
Were the study subjects and setting described in detail?	Low ROB. All included studies assessed neonatal herpes incidence outcomes among the entire population of live births within clearly defined geographic/catchment areas and specified time periods.
**5. Data coverage**	
Was the data analysis conducted with sufficient coverage of the identified sample?	Low ROB. All included studies calculated neonatal herpes incidence outcomes based on all identified cases among the entire population of live births within clearly defined geographic/catchment areas and specified time periods.
**6. Case definition**	
Was the case definition clearly defined?	Studies were classified as low ROB if neonatal status was clearly and adequately defined, including an explicit statement of the period used to define neonatal herpes (e.g., 0–29 days or 0–90 days). Studies were considered high ROB if neonatal status was unclear or inadequately defined, such as lacking specification of the time period during which neonatal herpes cases were identified.
**7. Diagnostic methods**	
Were valid methods used for identification of the condition?	This systematic review was designed with strict inclusion criteria requiring neonatal herpes cases to be diagnosed using valid, objective, and standardized approaches within well-defined populations, catchment areas, and timeframes. Neonatal herpes cases were required to be diagnosed either through laboratory testing or appropriate clinical evaluation. Accordingly, studies meeting these criteria were classified as low ROB.<br>However, some studies were included but classified as high ROB if, despite the study context suggesting that appropriate diagnostic methods were applied, the methods for diagnosing neonatal herpes were not clearly described (e.g., unspecified or unclear clinical/laboratory procedures).
**8. Statistical analysis**	
Was there appropriate statistical analysis?	Low ROB. All included studies calculated neonatal herpes incidence outcomes based on all identified cases among the entire population of live births within clearly defined geographic/catchment areas and specified time periods. Definitions of both the numerator and denominator were unambiguous and consistent.
**9. Confounding factors**	
Were important confounders identified and appropriately handled?	Low ROB. All included studies calculated neonatal herpes incidence outcomes based on all identified cases among the entire population of live births within clearly defined geographic/catchment areas and specified time periods. Definitions of both the numerator and denominator were unambiguous and consistent. Adjustment for confounding factors was not applicable, as the outcomes were based on complete population-level data.
**10. Subpopulation criteria**	

Were criteria for defining and reporting subpopulations clearly specified and applied?	Low ROB. All included studies calculated neonatal herpes incidence outcomes based on all identified cases among the entire population of live births within clearly defined geographic/catchment areas and specified time periods. None of the studies assessed incidence outcomes within specific subpopulations of live births or among specific subpopulations of pregnant women.

Abbreviations: HSV-1; Herpes simplex virus type 1; HSV-2, Herpes simplex virus type 2; ROB, Risk of bias.

Box S4. Variables included in meta-regression analyses. Factors (variables) chosen a priori and integrated into both the univariable and multivariable meta-regression analyses in this study.

1- WHO region as defined in Box S2
2- Country as defined in Box S2
3- Site type
   - National surveillance registry/report
   - Clinical setting (e.g., hospital, neonatal intensive care unit, or paediatric clinic)
   - Healthcare databases
   - Virological laboratory
   - Mixed/Unclear
4- Estimate type
   - National estimate
   - Regional estimate (e.g., state, province, city, or county)
   - Institutional estimate
5- Criteria for defining the neonatal period
   - 0 to 29 days
   - 0 to 90 days
   - Unclear
6- Method of ascertainment
   - Laboratory confirmation
   - Laboratory and/or clinical confirmation
   - Unclear
7- Year of data collection category[a]
   - <1995
   - 1995-2004
   - ≥2005
8- Year of data collection as a continuous term

Abbreviations: WHO, World Health Organization.
[a]The categories were determined based on the median time between the year of publication and the year of data collection, which was approximately 6 years. This interval was rounded to 5 years to have a 5-year bracket.

Table S3. Studies reporting neonatal herpes incidence rates, categorized by World Health Organization region.

Country	Name	Year(s) of data collection	Estimate type	Method of ascertainment	Live births (N)[a]	nHSV cases (n)[b]	Incidence rate (per 100,000 live births)[c]	95% CI (per 100,000 live births)[d]
**Region of the Americas**								
Canada	Kropp, 2006 [7]	2000-2003	National	Laboratory confirmation	983,051	58	5.9	4.6-7.6
Canada	Selin, 1988 [8]	1980-1980	Regional	Laboratory confirmation	17,000	0	0.0	0.2-21.7
Canada	Selin, 1988 [8]	1981-1981	Regional	Laboratory confirmation	17,000	1	5.9	1.4-32.8
Canada	Selin, 1988 [8]	1982-1982	Regional	Laboratory confirmation	17,000	1	5.9	1.4-32.8
Canada	Selin, 1988 [8]	1983-1983	Regional	Laboratory confirmation	17,000	3	17.6	6.4-51.6
Canada	Selin, 1988 [8]	1984-1984	Regional	Laboratory confirmation	17,000	5	29.4	6.4-51.6
Canada	Selin, 1988 [8]	1985-1985	Regional	Laboratory confirmation	17,000	0	0.0	0.1-21.7
Canada	Selin, 1988 [8]	1986-1986	Regional	Laboratory confirmation	17,000	2	11.8	3.6-42.5
United States	Boehm, 1981 [9]	1976-1980	Regional	Laboratory confirmation	100,000	7	7.0	3.5-14.4
United States	Brown, 1991 [10]	1984-1989	Regional	Laboratory confirmation	18,630	10	53.7	29.5-98.7
United States	Brown, 2003 [11]	1982-1999	Regional	Unclear	58,362	18	30.8	19.6-48.7
United States	Chen, 2024 [12]	2009-2020	Regional	Laboratory confirmation	1,057,061	1,090	103.1	97.2-109.4
United States	Desai, 2011 [13]	2011-2011	Institutional	Unclear	55,670	84	151.0	122.1-186.9
United States	Dinh, 2008 [14]	2000-2004	Regional	Laboratory and/or clinical confirmation	55,556	1	1.8	0.4-10.0
United States	Dinh, 2008 [14]	2000-2004	Regional	Laboratory and/or clinical confirmation	250,000	12	4.8	2.8-8.4
United States	Dinh, 2008 [14]	2000-2004	Regional	Laboratory and/or clinical confirmation	327,869	20	6.1	4.0-9.4
United States	Dinh, 2008 [14]	2000-2004	Regional	Laboratory and/or clinical confirmation	400,000	4	1.0	0.4-2.6
United States	Dinh, 2008 [14]	2000-2004	Regional	Laboratory and/or clinical confirmation	127,660	6	4.7	2.2-10.2
United States	Dinh, 2008 [14]	2000-2004	Regional	Laboratory and/or clinical confirmation	754,717	40	5.3	3.9-7.2
United States	Dinh, 2008 [14]	2000-2004	Regional	Laboratory and/or clinical confirmation	54,348	15	27.6	16.8-45.5
United States	Dinh, 2008 [14]	2000-2004	Regional	Laboratory and/or clinical confirmation	400,000	14	3.5	2.1-5.9
United States	Donda, 2019 [15]	2003-2005	National	Laboratory and/or clinical confirmation	10,218,156	814	8.0	7.4-8.5
United States	Donda, 2019 [15]	2006-2008	National	Laboratory and/or clinical confirmation	10,710,853	762	7.1	6.6-7.6
United States	Donda, 2019 [15]	2009-2011	National	Laboratory and/or clinical confirmation	9,980,522	894	9.0	8.4-9.6
United States	Donda, 2019 [15]	2012-2014	National	Laboratory and/or clinical confirmation	11,877,289	1,160	9.8	9.2-10.3
United States	Flagg, 2011 [16]	2006-2006	National	Laboratory and/or clinical confirmation	4,106,488	682	16.6	14.4-18.8
United States	Gutierrez, 1999 [17]	1985-1985	Regional	Laboratory and/or clinical confirmation	470,816	55	11.7	9.0-15.2
United States	Gutierrez, 1999 [17]	1990-1990	Regional	Laboratory and/or clinical confirmation	611,666	69	11.3	8.9-14.3
United States	Gutierrez, 1999 [17]	1995-1995	Regional	Laboratory and/or clinical confirmation	551,226	63	11.4	8.9-14.6
United States	Handel, 2011 [18]	1997-2008	Regional	Laboratory and/or clinical confirmation	1,516,949	179	11.8	10.2-13.7
United States	Mahant, 2019 [19]	2009-2009	National	Unclear	289,903	94	32.4	28.0-42.0
United States	Mahant, 2019 [19]	2010-2010	National	Unclear	271,634	88	32.4	28.0-42.0
United States	Mahant, 2019 [19]	2011-2011	National	Unclear	224,809	71	31.6	26.0-42.0
United States	Mahant, 2019 [19]	2012-2012	National	Unclear	294,414	147	49.9	45.0-62.0
United States	Mahant, 2019 [19]	2013-2013	National	Unclear	286,604	145	50.6	45.0-62.0
United States	Mahant, 2019 [19]	2014-2014	National	Unclear	369,052	168	45.5	41.0-56.0
United States	Mahant, 2019 [19]	2015-2015	National	Unclear	369,808	187	50.6	46.0-61.0
United States	Mahnert, 2007 [20]	1999-2003	Institutional	Laboratory confirmation	78,115	4	5.1	1.4-13.1
United States	Mark, 2006 [21]	1987-2002	Regional	Unclear	1,089,595	91	8.4	6.8-10.3
United States	Matthias, 2021 [22]	2011-2017	Regional	Laboratory confirmation	1,534,140	483	31.5	25.0-41.3
United States	Morris, 2008 [23]	1995-2003	Regional	Unclear	4,782,094	580	12.1	11.2-13.2
United States	Nahmias, 1985 [24]	1975-1980	Institutional	Unclear	3,500	1	28.6	6.9-159.0
United States	Owusu-Edusei, 2015 [25]	2005-2005	National	Laboratory and/or clinical confirmation	58,423	17	29.1	18.3-46.6
United States	Owusu-Edusei, 2015 [25]	2006-2006	National	Laboratory and/or clinical confirmation	62,038	14	22.6	13.5-37.9

United States	Owusu-Edusei, 2015 [25]	2007-2007	National	Laboratory and/or clinical confirmation	109,063	38	34.8	25.4-47.8
United States	Owusu-Edusei, 2015 [25]	2008-2008	National	Laboratory and/or clinical confirmation	111,766	29	25.9	18.1-37.3
United States	Owusu-Edusei, 2015 [25]	2009-2009	National	Laboratory and/or clinical confirmation	133,603	52	38.9	29.7-51.0
United states	Pooser, 2023 [26]	2019-2019	National	Unclear	3,573,248	561	15.7	14.5-17.1
United States	Roberts, 1995 [27]	1984-1991	Institutional	Laboratory confirmation	100,000	3	3.0	1.1-8.8
United states	Saremi, 2024 [28]	2015-2019	Regional	Laboratory confirmation	100,000	2	2.1	0.6-7.3
United states	Saremi, 2024 [28]	2015-2019	Regional	Laboratory confirmation	100,000	2	2.3	0.7-7.7
United states	Saremi, 2024 [28]	2015-2019	Regional	Laboratory confirmation	100,000	31	31.5	22.3-44.6
United States	DOH of New York, 2007 [29]	2007-2007	Regional	Laboratory confirmation	127,273	7	5.5	2.7-11.3
United States	DOH of New York, 2008 [30]	2008-2008	Regional	Laboratory confirmation	253,165	40	15.8	11.6-21.5
United States	DOH of New York, 2009 [31]	2009-2009	Regional	Laboratory confirmation	250,000	25	10.0	6.8-14.8
United States	DOH of New York, 2010 [32]	2010-2010	Regional	Laboratory confirmation	246,032	31	12.6	8.9-17.9
United States	DOH of New York, 2011 [33]	2011-2011	Regional	Laboratory confirmation	243,243	27	11.1	7.6-16.1
United States	DOH of New York, 2012 [34]	2012-2012	Regional	Laboratory confirmation	240,310	31	12.9	9.1-18.3
United States	DOH of New York, 2013 [35]	2013-2013	Regional	Laboratory confirmation	240,000	24	10.0	6.4-14.9
United States	DOH of New York, 2014 [36]	2014-2014	Regional	Laboratory confirmation	235,772	29	12.3	8.6-17.7
United States	DOH of New York, 2015 [37]	2015-2015	Regional	Laboratory confirmation	239,130	22	9.2	6.1-13.9
United States	DOH of New York, 2016 [38]	2016-2016	Regional	Laboratory confirmation	242,857	34	14.0	10.0-19.6
United States	DOH of New York, 2017 [39]	2017-2017	Regional	Laboratory confirmation	250,000	20	8.0	5.1-12.4
United States	DOH of New York, 2018 [40]	2018-2018	Regional	Laboratory confirmation	440,000	22	5.0	3.3-7.6
United States	DOH of New York, 2019 [41]	2019-2019	Regional	Laboratory confirmation	16,500,000	33	0.2	0.1-0.3
United States	DOH of New York, 2020 [42]	2020-2020	Regional	Laboratory confirmation	11,000,000	11	0.1	0.1-0.2
United States	DOH of New York, 2021 [43]	2021-2021	Regional	Laboratory confirmation	18,000,000	18	0.1	0.1-0.2
United States	DOH of New York, 2022 [44]	2022-2022	Regional	Laboratory confirmation	16,500,000	33	0.2	0.1-0.3
United States	Stone, 1989 [45]	1984-1984	National	Laboratory confirmation	3,700,000	139	3.8	3.2-4.4
United States	Sullivan Bolyai, 1983 [46]	1966-1969	Regional	Laboratory and/or clinical confirmation	115,385	3	2.6	0.0-3.8
United States	Sullivan Bolyai, 1983 [46]	1970-1973	Regional	Laboratory and/or clinical confirmation	83,333	4	4.8	0.0-10.1
United States	Sullivan Bolyai, 1983 [46]	1974-1977	Regional	Laboratory and/or clinical confirmation	81,081	6	7.4	0.2-14.3
United States	Sullivan Bolyai, 1983 [46]	1978-1981	Regional	Laboratory and/or clinical confirmation	126,050	15	11.9	3.6-20.1
United States	Sullivan Bolyai, 1983 [46]	1982-1982	Regional	Laboratory and/or clinical confirmation	24,823	7	28.2	17.4-46.0
United States	Warford, 1986 [47]	1980-1980	Institutional	Laboratory confirmation	23,719	0	0.0	0.1-15.6
United States	Warford, 1986 [47]	1982-1982	Institutional	Laboratory confirmation	23,719	2	8.4	2.6-30.5
United States	Warford, 1986 [47]	1983-1983	Institutional	Laboratory confirmation	23,719	2	8.4	2.6-30.5
United States	Warford, 1986 [47]	1984-1984	Institutional	Laboratory confirmation	23,719	6	25.3	11.9-55.0
United States	Whitley, 2007 [48]	1997-2002	National	Laboratory and/or clinical confirmation	233,487	178	76.2	65.8-88.3
United States	Xu, 2008 [49]	1997-2002	Regional	Laboratory confirmation	270,703	24	8.9	5.4-12.4
**European Region**								
Denmark	Dungu, 2023 [50]	2010-2019	National	Laboratory confirmation	596,927	54	9.0	7.0-12.0
Denmark	Fonnest, 1997 [51]	1977-1984	National	Laboratory and/or clinical confirmation	423,729	10	2.4	1.3-4.3
Denmark	Fonnest, 1997 [51]	1984-1991	National	Laboratory and/or clinical confirmation	438,596	20	4.6	3.0-7.0
Finland	Koskiniemi, 1989 [52]	1970-1985	Regional	Laboratory confirmation	240,000	79	33.0	26.5-41.1
France	Bouthry, 2024 [53]	2013-2023	Institutional	Laboratory confirmation	666,667	8	1.2	0.0-3.3
Germany	Kidszun, 2022 [54]	2017-2018	National	Laboratory confirmation	1,574,468	37	2.4	1.7-3.0
Israel	Lagziel, 2024 [55]	201-2021	National	Laboratory confirmation	1,272,509	75	5.9	4.7-7.4
Israel	Kohelet, 2004 [56]	1999-2002	Institutional	Laboratory confirmation	6,953	11	158.2	89.2-282.9
Israel	Koren, 2013 [57]	2001-2007	National	Laboratory confirmation	261,488	22	8.4	5.6-12.7
Netherlands	Gaytant, 2000 [58]	1987-1991	National	Laboratory confirmation	950,000	19	2.0	1.3-3.1
Netherlands	Gaytant, 2000 [58]	1992-1998	National	Laboratory confirmation	1,358,000	33	2.4	1.7-3.4
Netherlands	Hemelaar, 2015 [59]	2006-2011	National	Laboratory confirmation	1,100,399	52	4.7	3.6-6.2
Netherlands	Poeran, 2008 [60]	1999-2005	National	Laboratory confirmation	1,031,250	33	3.2	2.3-4.5

Netherlands	van Everdingen, 1993 [61]	1981-1985	National	Laboratory confirmation	910,000	26	2.9	2.0-4.2
Netherlands	van Oeffelen, 2018 [62]	2012-2015	National	Laboratory and/or clinical confirmation	687,500	33	4.8	3.4-6.7
Spain	Lecumberri Garcia, 2015 [63]	2000-2015	Institutional	Laboratory confirmation	78,400	7	8.9	4.4-18.4
Sweden	Engman, 2008 [64]	1989-2000	Regional	Laboratory confirmation	267,690	19	7.1	4.6-11.1
Sweden	Forsgren, 1990 [65]	1979-1989	Regional	Laboratory confirmation	200,000	12	6.0	3.5-10.5
Switzerland	Kucera, 2012 [66]	2004-2007	Institutional	Unclear	6,507	0	0.0	0.4-56.7
Switzerland	Pascual, 2011 [67]	2002-2008	National	Laboratory confirmation	437,500	7	1.6	0.6-3.3
UK and Ireland	Dudley, 2023 [68]	2019-2022	National	Laboratory and/or clinical confirmation	1,710,145	118	6.9	5.8-8.3
UK and Ireland	Fidler, 2020 [69]	2019-2020	National	Laboratory and/or clinical confirmation	652,778	47	7.2	5.4-9.6
UK and Ireland	Fidler, 2021 [70]	2019-2021	National	Laboratory and/or clinical confirmation	855,072	59	6.9	4.2-6.5
UK and Ireland	Tookey, 1996 [71]	1986-1991	National	Laboratory and/or clinical confirmation	4,606,061	76	1.7	1.3-2.0
UK and Ireland	Tookey, 2020 [72]	2004-2006	National	Laboratory confirmation	2,374,302	85	3.6	2.9-4.4
UK	Batra, 2014 [73]	2006-2012	Regional	Laboratory confirmation	57,291	10	17.5	8.4-32.1
UK	Clarke, 2014 [74]	1986-1991	National	Unclear	4,560,000	76	1.7	1.3-2.1
UK	Clarke, 2014 [74]	2004-2006	National	Unclear	2,580,000	86	3.3	2.7-4.1
UK	Clarke, 2014 [74]	2012-2012	National	Laboratory confirmation	688,116	66	9.6	7.5-12.2
UK	Kadambari, 2020 [75]	1999-2006	National	Laboratory and/or clinical confirmation	5,428,864	233	4.3	3.7-5.0
UK	Kadambari, 2020 [75]	2007-2016	National	Laboratory and/or clinical confirmation	6,786,080	645	9.5	8.7-10.2
UK	Kadambari, 2020 [75]	1968-1998	National	Laboratory and/or clinical confirmation	1,111,111	10	0.9	0.4-1.6
UK	Sachane, 2017 [76]	2004-2015	Institutional	Unclear	67,500	8	11.9	6.1-23.4
**Western Pacific Region**								
Australia	Berkhout, 2021 [77]	2005-2017	Regional	Laboratory confirmation	791,667	76	9.6	7.0-11.5
Australia	Deverell, 2012 [78]	2011-2011	National	Laboratory confirmation	296,296	8	2.7	1.4-5.3
Australia	Deverell, 2013 [79]	2012-2012	National	Laboratory confirmation	302,013	9	2.98	1.6-5.7
Australia	Deverell, 2014 [80]	2013-2013	National	Laboratory and/or clinical confirmation	309,735	14	4.5	2.7-7.6
Australia	Deverell, 2016 [81]	2014-2014	National	Laboratory and/or clinical confirmation	307,692	10	3.3	1.8-6.0
Australia	Deverell, 2017a [82]	2015-2015	National	Laboratory confirmation	299,625	16	5.3	3.3-8.7
Australia	Deverell, 2017b [83]	2016-2016	National	Laboratory confirmation	306,122	6	2.0	0.9-4.3
Australia	Elder, 1995 [84]	1990-1991	Regional	Laboratory confirmation	37,440	5	13.4	4.5-42.6
Australia	Garland, 1992 [85]	1982-1988	Institutional	Laboratory confirmation	66,000	6	9.1	4.3-19.8
Australia	Jones, 2010 [86]	1997-2007	National	Laboratory and/or clinical confirmation	2,816,092	98	3.5	2.9-4.2
Australia	Mindel, 2000 [87]	1995-1998	Institutional	Laboratory confirmation	13,372	1	7.5	1.8-41.7
Australia	Teutsch, 2018 [88]	2017-2017	National	Laboratory and/or clinical confirmation	303,030	4	1.3	0.5-3.4
Australia	Teutsch, 2020 [89]	2019-2019	National	Laboratory confirmation	303,030	8	2.6	1.1-5.2
Australia	Teutsch, 2021 [90]	2020-2020	National	Laboratory confirmation	305,344	8	2.6	1.1-5.2
Australia	Teutsch, 2022 [91]	2021-2021	National	Laboratory confirmation	305,677	14	4.6	2.7-7.7
Australia	Teutsch, 2023 [92]	2022-2022	National	Laboratory confirmation	295,858	10	3.4	1.8-6.3
Australia	Zurynski, 2010 [93]	2008-2008	National	Laboratory and/or clinical confirmation	300,000	9	3.0	1.6-5.7
Australia	Zurynski, 2010 [93]	2009-2009	National	Laboratory and/or clinical confirmation	303,030	10	3.3	1.8-6.1
Australia	Zurynski, 2011 [94]	2010-2010	National	Laboratory and/or clinical confirmation	307,692	4	1.3	0.5-3.3
Japan	Morishima, 2000 [95]	1978-1982	National	Unclear	1,600,000	16	1.0	0.6-1.6
Japan	Morishima, 2000 [95]	1983-1983	National	Unclear	1,500,000	24	1.6	1.1-2.4
Japan	Morishima, 2000 [95]	1984-1984	National	Unclear	1,619,048	34	2.1	1.5-2.9
Japan	Morishima, 2000 [95]	1985-1985	National	Unclear	1,444,444	39	2.7	2.0-3.7
Japan	Morishima, 2000 [95]	1986-1986	National	Unclear	1,303,030	43	3.3	2.5-4.4
Japan	Morishima, 2000 [95]	1987-1987	National	Unclear	1,216,216	45	3.7	2.8-5.0
Japan	Torii, 2013 [96]	2006-2008	National	Laboratory confirmation	1,461,538	38	2.6	1.9-3.6
Japan	Yamada, 2015 [97]	2011-2011	National	Laboratory confirmation	788,673	7	0.9	0.4-1.8

Abbreviations: CI, Confidence interval; DOH, Department of Health; nHSV, Neonatal herpes simplex virus; UK, United Kingdom.

[a]If the number of live births was not explicitly reported in a study, it was estimated by dividing the number of nHSV cases by the reported incidence rate. When both live births and cases were not reported, the number of live births was estimated using the median live births from other studies within the same geographic area

[b]The number of nHSV cases was extracted directly from the study when explicitly reported. If not provided, it was calculated by multiplying the incidence rate by the number of live births. In some studies, the number of cases was estimated using statistical methods, such as capture-recapture, applied to reported cases from various sources. As a result, the estimated number of cases may not always be an exact integer.

[c]If a study employed multiple methods to estimate the incidence rate within the same geographic area and time period, the estimate from the method deemed most representative was extracted.

[d]If a study did not provide a 95% CI for the incidence rate, it was calculated using a Bayesian approach employing a Beta-binomial model.

Table S4. Studies reporting neonatal herpes proportions attributed to HSV-1 versus HSV-2, categorized by World Health Organization region.

Country	Name	Year of data collection	Laboratory method[a]	Typed nHSV cases	Proportion of HSV-1 (%)[b]	Proportion of HSV-2 (%)[b]
**Region of the Americas**						
Canada	Kropp, 2006 [7]	2000-2003	PCR, culture, and IgM serology	48	62.5	37.5
Canada	Selin, 1988 [8]	1980-1986	Mixed	11	45.5	54.5
United States	Boehm, 1981 [9]	1976-1980	Culture	7	28.6	71.4
United States	Brown, 1991 [10]	1984-1989	Culture	10	30.0	70.0
United States	Brown, 2003 [11]	1982-1999	PCR or culture	18	44.4	55.6
United States	Brown, 2007 [98]	2007-2007	PCR or culture	25	40.0	60.0
United States	Burchett, 1992 [99]	1983-1988	Culture	22	31.8	68.2
United States	Cantey, 2012 [100]	2005-2010	PCR	21	47.6	52.4
United States	Caviness, 2008a [101]	1991-2005	PCR, culture, and/or DFA	40	27.5	72.5
United States	Caviness, 2008b [102]	2001-2005	PCR, culture, and/or DFA	10	40.0	60.0
United States	CDC, 2012 [103]	2006-2011	PCR	70	52.9	47.1
United States	Ch'ien, 1975 [104]	2007-2007	Culture	11	36.4	63.6
United States	Corey, 1988 [105]	1981-1986	Culture	33	42.4	57.6
United States	Dendi, 2020 [106]	2001-2019	PCR	88	44.2	55.8
United States	Dominguez, 2018 [107]	2017-2017	PCR or culture	7	57.1	42.9
United States	Ericson, 2022 [108]	2002-2014	PCR or culture	44	36.4	63.6
United States	Graf, 2024 [109]	2001-2023	PCR	15	86.7	13.3
United States	Handel, 2011 [18]	2006-2010	Mixed	55	50.9	49.1
United States	Kimberlin, 2001 [110]	1989-1997	PCR or culture	74	27.0	73.0
United States	Kimberlin, 2011 [111]	1997-2008	PCR or culture	53	32.1	67.9
United states	Kotzbauer, 2014 [112]	2006-2013	PCR or culture	13	46.2	53.8
United States	Lao, 2019 [113]	2006-2015	Mixed	93	46.2	53.8
United States	Long, 2011 [114]	1988-2009	PCR or culture	17	11.8	88.2
United States	Mahnert, 2007 [20]	1999-2003	PCR or culture	3	33.3	66.7
United States	Matthias, 2021 [22]	2011-2017	PCR or culture	164	44.5	55.5
United States	Mejias, 2023 [115]	2007-2022	PCR or culture	12	58.3	41.7
United States	Melvin, 2022 [116]	1980-1989	Culture	43	34.9	65.1
United States	Melvin, 2022 [116]	1990-1999	PCR or culture	29	44.8	55.2
United States	Melvin, 2022 [116]	2000-2009	PCR or culture	38	44.7	55.3
United States	Melvin, 2022 [116]	2010-2016	PCR or culture	21	57.1	42.9
United States	Nahmias, 1969 [117]	2007-2007	Culture	25	12.0	88.0
United states	O'Riordan, 2006 [118]	1989-2003	PCR or culture	12	0.0	100
United States	Pass, 1981 [119]	2007-2007	Mixed	11	18.2	81.8
United States	Schillinger, 2013 [120]	2006-2012	PCR	76	52.6	47.4
United States	Stone, 1989 [45]	1983-1985	Culture	105	32.4	67.6
United States	Sullender, 1987 [121]	1976-1985	Mixed	55	29.1	70.9
United States	Sullivan Bolyai, 1983 [46]	1965-1982	Neutralization	27	22.2	77.8
United States	Whitley, 1988 [122]	1973-1981	Mixed	80	26.3	73.8
United States	Whitley, 1991 [123]	1981-1988	Mixed	184	35.3	64.7
United States	Xu, 2008 [49]	1997-2002	PCR, culture, and/or DFA	17	41.2	58.8
United States	Samies, 2019 [124]	2014-2018	PCR or culture	18	66.7	33.3
United States	Yeager, 1980 [125]	1976-1979	Culture	11	27.3	72.7
United States	Yeager, 1984 [126]	-	Mixed	19	52.6	47.4
United States and Canada	Cruz, 2018 [127]	2005-2013	PCR or culture	96	55.2	46.9
**European Region**						
Denmark	Dungu, 2023 [50]	2010-2019	PCR	53	98.1	1.9
Denmark	Fonnest, 1997 [51]	1977-1984	Culture	7	42.9	57.1
Denmark	Fonnest, 1997 [51]	1984-1991	Culture	2	50.0	50.0
Finland	Koskiniemi, 1989 [52]	1970-1985	Mixed	13	7.7	92.3

France	Bouthry, 2024 [53]	2013-2023	PCR	8	50.0	50.0
Germany	Kidszun, 2022 [54]	2017-2018	PCR or culture	24	83.3	25.0
Israel	Kohelet, 2004 [56]	1999-2002	PCR or culture	5	0.0	100
Israel	Koren, 2013 [57]	2001-2007	PCR or culture	3	33.3	66.7
Israel	Lagziel, 2024 [55]	2015-2022	PCR or culture	85	90.6	9.4
Israel	Leventon-Kriss, 1983 [128]	1973-1981	Neutralization	17	76.5	23.5
Netherlands	Gaytant, 2000 [58]	1992-1998	Culture	27	88.9	11.1
Netherlands	Hemelaar, 2015 [59]	2006-2011	PCR or culture	35	94.3	5.7
Netherlands	Keunig, 2019	2006-2017	PCR	6	33.3	66.7
Netherlands	Poeran, 2008 [60]	1999-2005	PCR or culture	28	53.6	46.4
Netherlands	van Everdingen, 1993 [61]	1981-1985	Culture	22	68.2	31.8
Netherlands	van Everdingen, 1993 [61]	1987-1991	Culture	16	75.0	25.0
Netherlands	van Oeffelen, 2018 [62]	2012-2015	PCR	23	78.3	21.7
Sweden	Brown, 2007 [98]	1973-2001	PCR or culture	35	28.6	71.4
Sweden	Engman, 2008 [64]	1989-2000	Mixed	9	22.2	77.8
Sweden	Forsgren, 1990 [65]	1979-1989	PCR	48	25.0	75.0
Sweden	Lewensohn-Fuchs, 2003 [129]	1979-1998	Mixed	28	10.7	89.3
Sweden	Malm, 1991 [130]	1971-1986	Mixed	45	22.2	77.8
Sweden	Malm, 1999 [131]	1973-1996	PCR	36	19.4	80.6
Switzerland	Pascual, 2011 [67]	2002-2008	PCR, culture, and IgM serology	6	66.7	33.3
UK and Ireland	Dudley, 2023 [68]	2019-2022	PCR	78	37.0	63.0
UK and Ireland	Fidler, 2021 [70]	2019-2021	Mixed	54	53.7	46.3
UK and Ireland	Tookey, 1996 [71]	1986-1991	Mixed	49	51.0	49.0
UK and Ireland	Tookey, 2007 [132]	2004-2004	Mixed	24	45.8	54.2
UK and Ireland	Tookey, 2007 [132]	2005-2005	Mixed	31	48.4	51.6
UK and Ireland	Tookey, 2007 [132]	2006-2006	Mixed	24	37.5	62.5
UK	Batra, 2014 [73]	2006-2013	PCR	17	52.9	47.1
UK	Kashyap, 2014 [133]	2010-2013	Mixed	11	54.5	45.5
UK	Khandwalla, 2021 [134]	2010-2019	PCR	10	10.0	90.0
UK	Vaughan, 2024 [135]	-	Mixed	4	75.0	25.0
UK	Waheed, 2022 [136]	2013-2020	Mixed	20	30.0	70.0
**Western Pacific Region**						
Australia	Tiffany, 2005 [137]	1990-2003	PCR or culture	14	21.4	78.6
Australia	Berkhout, 2021 [77]	2005-2017	PCR	144	72.2	27.8
Australia	Deverell, 2012 [78]	2011-2011	PCR or culture	10	70.0	30.0
Australia	Deverell, 2017a [82]	2015-2015	PCR or culture	16	68.8	31.3
Australia	Deverell, 2017b [83]	2016-2016	PCR or culture	6	83.3	16.7
Australia	Elder, 1995 [84]	1977-1992	Mixed	13	7.7	92.3
Australia	Garland, 1992 [85]	1982-1988	Mixed	6	50.0	50.0
Australia	Jones, 2005 [138]	2004-2004	Mixed	8	62.5	37.5
Australia	Jones, 2014 [139]	1997-2011	Mixed	118	62.7	37.3
Australia	Malouf, 1995 [140]	1979-1992	Mixed	9	11.1	88.9
Australia	Nunez, 2019 [141]	2018-2018	PCR	4	75.0	25.0
Australia	Teutsch, 2020 [89]	2019-2019	PCR	8	50.0	50.0
Australia	Teutsch, 2021 [90]	2020-2020	PCR	8	75.0	25.0
Australia	Teutsch, 2022 [91]	2021-2021	PCR	14	78.6	21.4
Australia	Teutsch, 2023 [92]	2022-2022	PCR or immunofluorescence	10	50.0	50.0
Japan	Kawada, 2004 [142]	-	PCR	19	47.4	52.6
Japan	Kimura, 2002 [143]	2000-2000	PCR	34	64.7	35.3
Japan	Morishima, 2000 [95]	1978-1987	Culture	65	67.7	32.3
Japan	Morishima, 2000 [95]	1988-1991	Culture	12	58.3	41.7
Japan	Morishima, 2000 [95]	1992-1993	Culture	18	77.8	22.2
Japan	Morishima, 2000 [95]	1994-1999	Culture	39	64.1	35.9
Japan	Okanishi, 2015 [144]	2001-2011	PCR	6	16.7	83.3
Japan	Torii, 2013 [96]	2006-2008	Mixed	13	53.8	46.2

Republic of Korea	Kim, 2018 [145]	2008-2017	Mixed	16	62.5	37.5

Abbreviations: CDC, Centers for Disease Control and Prevention; CI, Confidence interval; DFA, Direct fluorescence assay; HSV-1, Herpes simplex virus type 1; HSV-2, Herpes simplex virus type 2; IgM, Immunoglobulin M; nHSV, Neonatal herpes simplex virus; PCR, Polymerase chain reaction; UK, United Kingdom.

[a]Mixed refers to the use of a combination of different laboratory methods, including PCR, culture, and other diagnostic techniques.

[b]Cases with an unknown nHSV type were excluded from the total to ensure accurate measurement of the proportions of cases attributed to HSV-1 and HSV-2. If a case tested positive for both HSV-1 and HSV-2, it was included in both categories. As a result, the proportions attributed to HSV-1 and HSV-2 may not always sum to 100%.

Figure S1. Risk of bias assessment of individual incidence rate studies. Traffic light plot of the risk of bias assessment for individual studies reporting incidence rates of neonatal herpes, based on the Joanna Briggs Institute's critical appraisal tool [4-6].

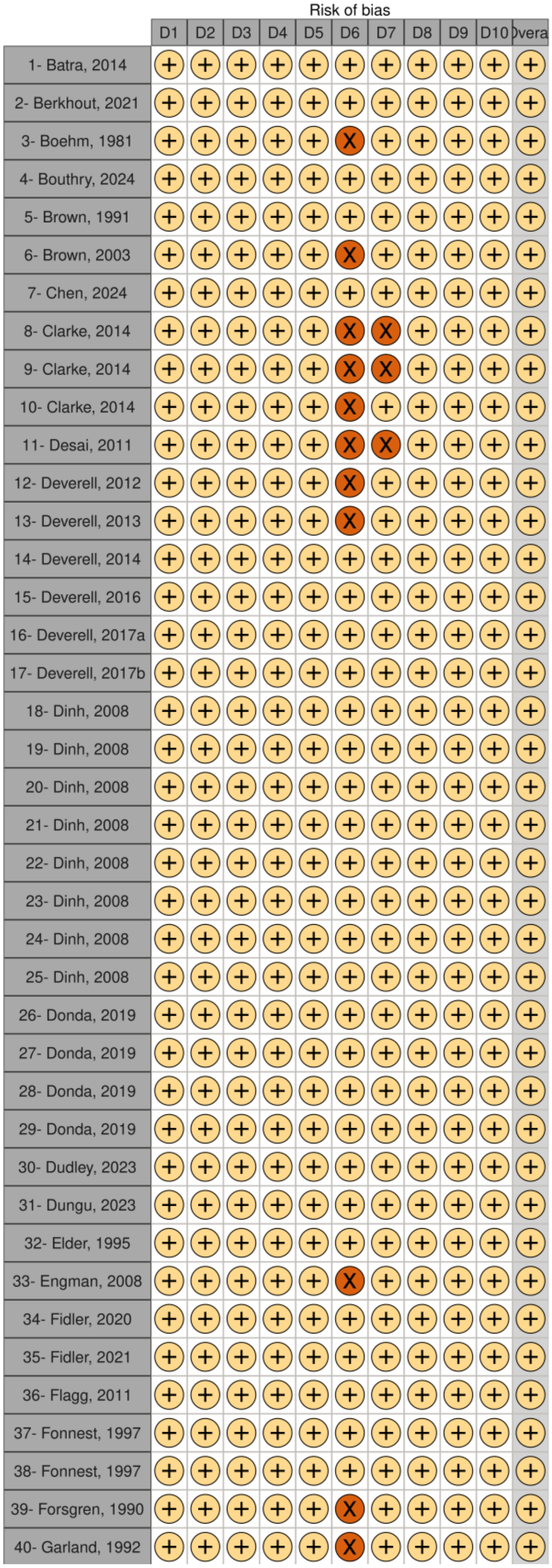

Risk of bias

	D1	D2	D3	D4	D5	D6	D7	D8	D9	D10	vera
41- Gaytant, 2000	+	+	+	+	+	+	+	+	+	+	+
42- Gaytant, 2000	+	+	+	+	+	+	+	+	+	+	+
43- Gutierrez, 1999	+	+	+	+	+	+	+	+	+	+	+
44- Gutierrez, 1999	+	+	+	+	+	+	+	+	+	+	+
45- Gutierrez, 1999	+	+	+	+	+	+	+	+	+	+	+
46- Handel, 2011	+	+	+	+	+	+	+	+	+	+	+
47- Hemelaar, 2015	+	+	+	+	+	+	+	+	+	+	+
48- Jones, 2010	+	+	+	+	+	+	+	+	+	+	+
49- Kadambari, 2020	+	+	+	+	+	+	+	+	+	+	+
50- Kadambari, 2020	+	+	+	+	+	+	+	+	+	+	+
51- Kadambari, 2020	+	+	+	+	+	+	+	+	+	+	+
52- Kidszun, 2022	+	+	+	+	+	+	+	+	+	+	+
53- Kohelet, 2004	+	+	+	+	+	X	+	+	+	+	+
54- Koren, 2013	+	+	+	+	+	+	+	+	+	+	+
55- Koskiniemi, 1989	+	+	+	+	+	+	+	+	+	+	+
56- Kropp, 2006	+	+	+	+	+	+	+	+	+	+	+
57- Kucera, 2012	+	+	+	+	+	X	X	+	+	+	+
58- Lagziel, 2024	+	+	+	+	+	+	+	+	+	+	+
59- Lecumberri Garcia, 2015	+	+	+	+	+	X	+	+	+	+	+
60- Mahant, 2019	+	+	+	+	+	+	X	+	+	+	+
61- Mahant, 2019	+	+	+	+	+	+	X	+	+	+	+
62- Mahant, 2019	+	+	+	+	+	+	X	+	+	+	+
63- Mahant, 2019	+	+	+	+	+	+	X	+	+	+	+
64- Mahant, 2019	+	+	+	+	+	+	X	+	+	+	+
65- Mahant, 2019	+	+	+	+	+	+	X	+	+	+	+
66- Mahant, 2019	+	+	+	+	+	+	X	+	+	+	+
67- Mahnert, 2007	+	+	+	+	+	+	+	+	+	+	+
68- Mark, 2006	+	+	+	+	+	+	+	+	+	+	+
69- Matthias, 2021	+	+	+	+	+	+	+	+	+	+	+
70- Mindel, 2000	+	+	+	+	+	X	+	+	+	+	+
71- Morishima, 2000	+	+	+	+	+	X	+	+	+	+	+
72- Morishima, 2000	+	+	+	+	+	X	+	+	+	+	+
73- Morishima, 2000	+	+	+	+	+	X	+	+	+	+	+
74- Morishima, 2000	+	+	+	+	+	X	+	+	+	+	+
75- Morishima, 2000	+	+	+	+	+	X	+	+	+	+	+
76- Morishima, 2000	+	+	+	+	+	X	+	+	+	+	+
77- Morris, 2008	+	+	+	+	+	+	X	+	+	+	+
78- Nahmias, 1985	+	+	+	+	+	X	X	+	+	+	+
79- Owusu-Edusei, 2015	+	+	+	+	+	X	+	+	+	+	+
80- Owusu-Edusei, 2015	+	+	+	+	+	X	+	+	+	+	+

Risk of bias

	D1	D2	D3	D4	D5	D6	D7	D8	D9	D10	vera
81- Owusu-Edusei, 2015	+	+	+	+	+	X	+	+	+	+	+
82- Owusu-Edusei, 2015	+	+	+	+	+	X	+	+	+	+	+
83- Owusu-Edusei, 2015	+	+	+	+	+	X	+	+	+	+	+
84- Pascual, 2011	+	+	+	+	+	+	+	+	+	+	+
85- Poeran, 2008	+	+	+	+	+	+	+	+	+	+	+
86- Pooser, 2023	+	+	+	+	+	X	+	+	+	+	+
87- Roberts, 1995	+	+	+	+	+	X	+	+	+	+	+
88- Sachane, 2017	+	+	+	+	+	+	X	+	+	+	+
89- Saremi, 2024	+	+	+	+	+	+	+	+	+	+	+
90- Saremi, 2024	+	+	+	+	+	+	+	+	+	+	+
91- Saremi, 2024	+	+	+	+	+	+	+	+	+	+	+
92- Selin, 1988	+	+	+	+	+	X	+	+	+	+	+
93- Selin, 1988	+	+	+	+	+	X	+	+	+	+	+
94- Selin, 1988	+	+	+	+	+	X	+	+	+	+	+
95- Selin, 1988	+	+	+	+	+	X	+	+	+	+	+
96- Selin, 1988	+	+	+	+	+	X	+	+	+	+	+
97- Selin, 1988	+	+	+	+	+	X	+	+	+	+	+
98- Selin, 1988	+	+	+	+	+	X	+	+	+	+	+
99- State of New York DOH, 2007	+	+	+	+	+	+	+	+	+	+	+
100- State of New York DOH, 2008	+	+	+	+	+	+	+	+	+	+	+
101- State of New York DOH, 2009	+	+	+	+	+	+	+	+	+	+	+
102- State of New York DOH, 2010	+	+	+	+	+	+	+	+	+	+	+
103- State of New York DOH, 2011	+	+	+	+	+	+	+	+	+	+	+
104- State of New York DOH, 2012	+	+	+	+	+	+	+	+	+	+	+
105- State of New York DOH, 2013	+	+	+	+	+	+	+	+	+	+	+
106- State of New York DOH, 2014	+	+	+	+	+	+	+	+	+	+	+
107- State of New York DOH, 2015	+	+	+	+	+	+	+	+	+	+	+
108- State of New York DOH, 2016	+	+	+	+	+	+	+	+	+	+	+
109- State of New York DOH, 2017	+	+	+	+	+	+	+	+	+	+	+
110- State of New York DOH, 2018	+	+	+	+	+	+	+	+	+	+	+
111- State of New York DOH, 2019	+	+	+	+	+	+	+	+	+	+	+
112- State of New York DOH, 2020	+	+	+	+	+	+	+	+	+	+	+
113- State of New York DOH, 2021	+	+	+	+	+	+	+	+	+	+	+
114- State of New York DOH, 2022	+	+	+	+	+	+	+	+	+	+	+
115- Stone, 1989	+	+	+	+	+	+	+	+	+	+	+
116- Sullivan Bolyai, 1983	+	+	+	+	+	+	+	+	+	+	+
117- Sullivan Bolyai, 1983	+	+	+	+	+	+	+	+	+	+	+
118- Sullivan Bolyai, 1983	+	+	+	+	+	+	+	+	+	+	+
119- Sullivan Bolyai, 1983	+	+	+	+	+	+	+	+	+	+	+
120- Sullivan Bolyai, 1983	+	+	+	+	+	+	+	+	+	+	+

Risk of bias

	D1	D2	D3	D4	D5	D6	D7	D8	D9	D10	vera
121- Teutsch, 2018	+	+	+	+	+	+	+	+	+	+	+
122- Teutsch, 2020	+	+	+	+	+	+	+	+	+	+	+
123- Teutsch, 2021	+	+	+	+	+	+	+	+	+	+	+
124- Teutsch, 2022	+	+	+	+	+	+	+	+	+	+	+
125- Teutsch, 2023	+	+	+	+	+	+	+	+	+	+	+
126- Tookey, 1996	+	+	+	+	+	X	+	+	+	+	+
127- Tookey, 2020	+	+	+	+	+	+	+	+	+	+	+
128- Torii, 2013	+	+	+	+	+	X	+	+	+	+	+
129- van Everdingen, 1993	+	+	+	+	+	+	+	+	+	+	+
130- van Oeffelen, 2018	+	+	+	+	+	X	+	+	+	+	+
131- Warford, 1986	+	+	+	+	+	+	+	+	+	+	+
132- Warford, 1986	+	+	+	+	+	+	+	+	+	+	+
133- Warford, 1986	+	+	+	+	+	+	+	+	+	+	+
134- Warford, 1986	+	+	+	+	+	+	+	+	+	+	+
135- Whitley, 2007	+	+	+	+	+	+	+	+	+	+	+
136- Xu, 2008	+	+	+	+	+	+	+	+	+	+	+
137- Yamada, 2015	+	+	+	+	+	X	+	+	+	+	+
138- Zurynski, 2010	+	+	+	+	+	X	+	+	+	+	+
139- Zurynski, 2010	+	+	+	+	+	X	+	+	+	+	+
140- Zurynski, 2011	+	+	+	+	+	X	+	+	+	+	+

D1: Sample Representativeness
D2: Participant Recruitment
D3: Sample Size
D4: Description Of Study Subjects And Settings
D5: Data Analysis Coverage
D6: Case Definition Criteria
D7: Diagnostic Reliability
D8: Statistical Analysis Methods
D9: Confounding Factors
D10: Criteria For Subpopulations

Judgement
X High
\+ Low

Figure S2. Risk of bias assessment of individual HSV-1 versus HSV-2 studies. Traffic light plot of the risk of bias assessment for individual studies reporting neonatal herpes proportions attributed to HSV-1 versus HSV-2, based on the Joanna Briggs Institute's critical appraisal tool [4-6].

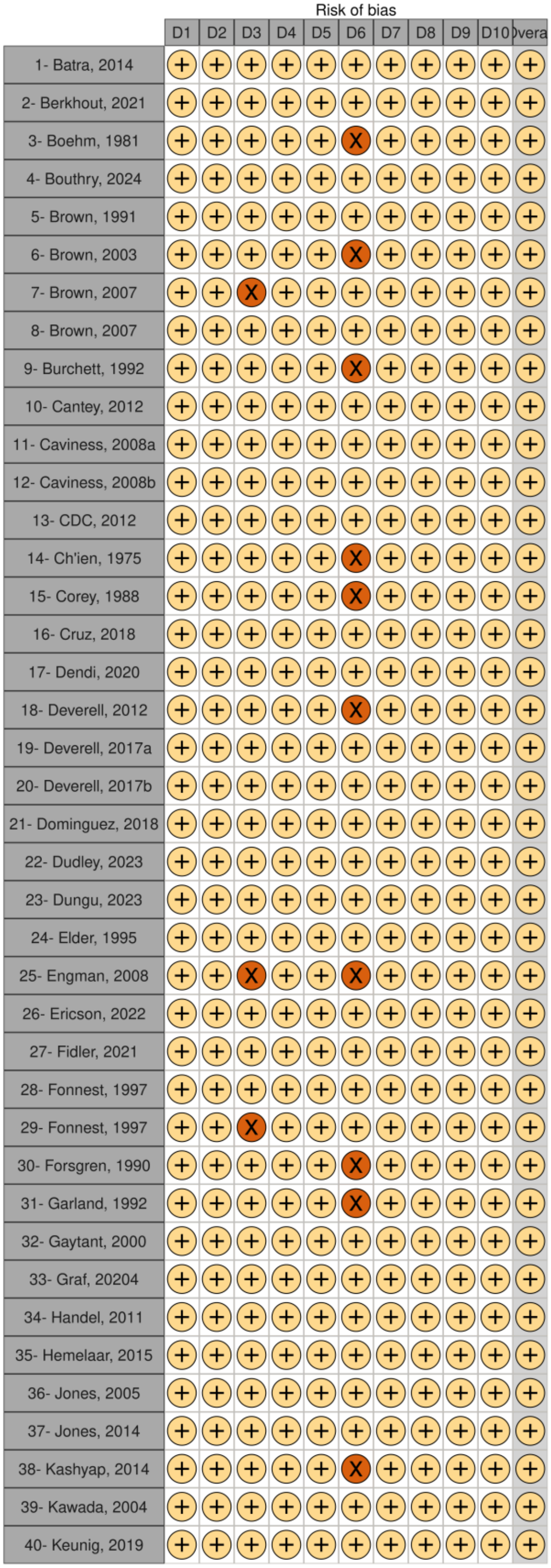

Risk of bias

	D1	D2	D3	D4	D5	D6	D7	D8	D9	D10	vera
41- Khandwalla, 2021	+	+	+	+	+	X	+	+	+	+	+
42- Kidszun, 2022	+	+	+	+	+	+	+	+	+	+	+
43- Kim, 2018	+	+	+	+	+	+	+	+	+	+	+
44- Kimberlin, 2001	+	+	+	+	+	+	+	+	+	+	+
45- Kimberlin, 2011	+	+	+	+	+	+	+	+	+	+	+
46- Kimura, 2002	+	+	+	+	+	+	+	+	+	+	+
47- Kohelet, 2004	+	+	X	+	+	X	+	+	+	+	+
48- Koren, 2013	+	+	+	+	+	+	+	+	+	+	+
49- Koskiniemi, 1989	+	+	X	+	+	+	+	+	+	+	+
50- Kotzbauer, 2014	+	+	+	+	+	+	+	+	+	+	+
51- Kropp, 2006	+	+	+	+	+	+	+	+	+	+	+
52- Lagziel, 2024	+	+	+	+	+	+	+	+	+	+	+
53- Lao, 2019	+	+	+	+	+	+	+	+	+	+	+
54- Leventon-Kriss, 1983	+	+	+	+	+	+	+	+	+	+	+
55- Lewensohn-Fuchs, 2003	+	+	+	+	+	+	+	+	+	+	+
56- Long, 2011	+	+	+	+	+	+	+	+	+	+	+
57- Mahnert, 2007	+	+	+	+	+	+	+	+	+	+	+
58- Malm, 1991	+	+	+	+	+	+	+	+	+	+	+
59- Malm, 1999	+	+	+	+	+	+	+	+	+	+	+
60- Malouf, 1995	+	+	+	+	+	+	+	+	+	+	+
61- Matthias, 2021	+	+	+	+	+	+	+	+	+	+	+
62- Mejias, 2023	+	+	X	+	+	+	+	+	+	+	+
63- Melvin, 2022	+	+	+	+	+	+	+	+	+	+	+
64- Melvin, 2022	+	+	+	+	+	+	+	+	+	+	+
65- Melvin, 2022	+	+	+	+	+	+	+	+	+	+	+
66- Melvin, 2022	+	+	+	+	+	+	+	+	+	+	+
67- Morishima, 2000	+	+	+	+	+	X	+	+	+	+	+
68- Morishima, 2000	+	+	+	+	+	X	+	+	+	+	+
69- Morishima, 2000	+	+	+	+	+	X	+	+	+	+	+
70- Morishima, 2000	+	+	+	+	+	X	+	+	+	+	+
71- Nahmias, 1969	+	+	+	+	+	X	+	+	+	+	+
72- Nunez, 2019	+	+	+	+	+	+	+	+	+	+	+
73- Okanishi, 2015	+	+	X	+	+	+	+	+	+	+	+
74- O'Riordan, 2006	+	+	+	+	+	+	+	+	+	+	+
75- Pascual, 2011	+	+	+	+	+	+	+	+	+	+	+
76- Pass, 1981	+	+	+	+	+	X	+	+	+	+	+
77- Poeran, 2008	+	+	+	+	+	+	+	+	+	+	+
78- Samies, 2019	+	+	+	+	+	+	+	+	+	+	+
79- Schillinger, 2013	+	+	+	+	+	+	+	+	+	+	+
80- Selin, 1988	+	+	+	+	+	+	+	+	+	+	+

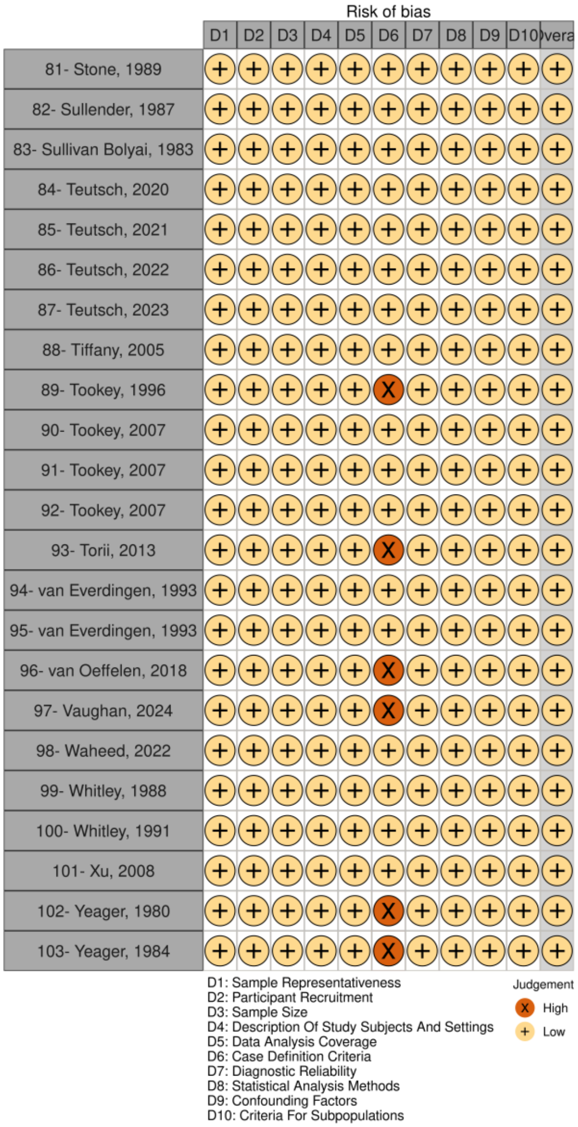
Risk of bias
D1
D2
D3
D4
D5
D6
D7
D8
D9
D10
81- Stone, 1989
82- Sullender, 1987
83- Sullivan Bolyai, 1983
84- Teutsch, 2020
85- Teutsch, 2021
86- Teutsch, 2022
87- Teutsch, 2023
88- Tiffany, 2005
89- Tookey, 1996
90- Tookey, 2007
91- Tookey, 2007
92- Tookey, 2007
93- Torii, 2013
94- van Everdingen, 1993
95- van Everdingen, 1993
96- van Oeffelen, 2018
97- Vaughan, 2024
98- Waheed, 2022
99- Whitley, 1988
100- Whitley, 1991
101- Xu, 2008
102- Yeager, 1980
103- Yeager, 1984
D1: Sample Representativeness
D2: Participant Recruitment
D3: Sample Size
D4: Description Of Study Subjects And Settings
D5: Data Analysis Coverage
D6: Case Definition Criteria
D7: Diagnostic Reliability
D8: Statistical Analysis Methods
D9: Confounding Factors
D10: Criteria For Subpopulations
Judgement
High
Low

Figure S3. Summary of risk of bias assessment of incidence rate studies. Summary plot of the risk of bias assessment of individual studies reporting incidence rate of neonatal herpes, based on the Joanna Briggs Institute's critical appraisal tool [4-6].

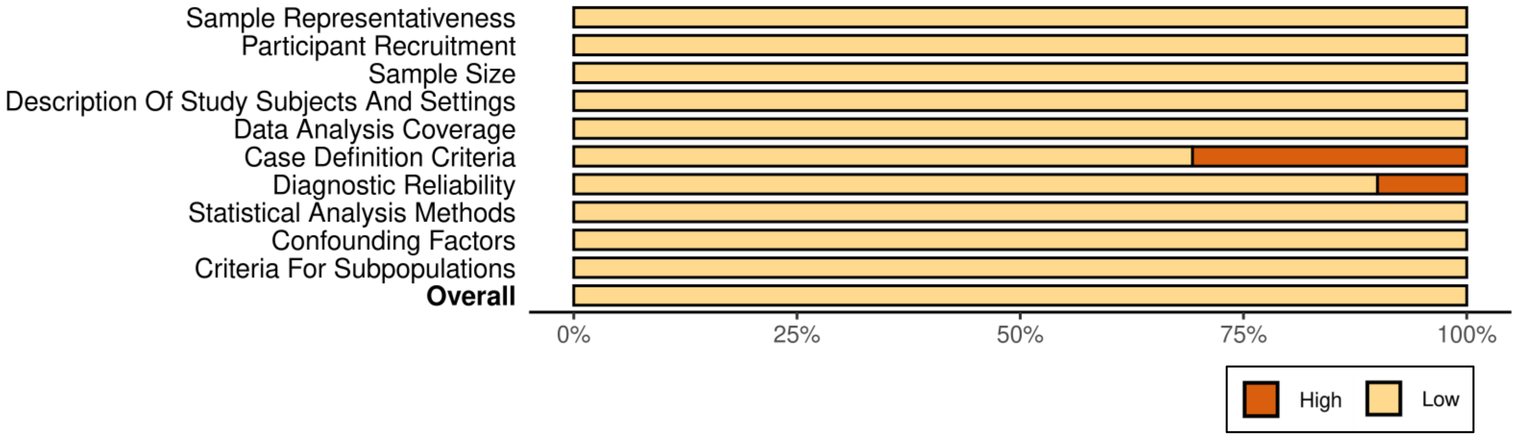

Figure S4. Summary of risk of bias assessment of HSV-1 versus HSV-2 studies. Summary plot of the risk of bias assessment of individual studies reporting neonatal herpes proportions attributed to HSV-1 versus HSV-2, based on the Joanna Briggs Institute's critical appraisal tool [4-6].

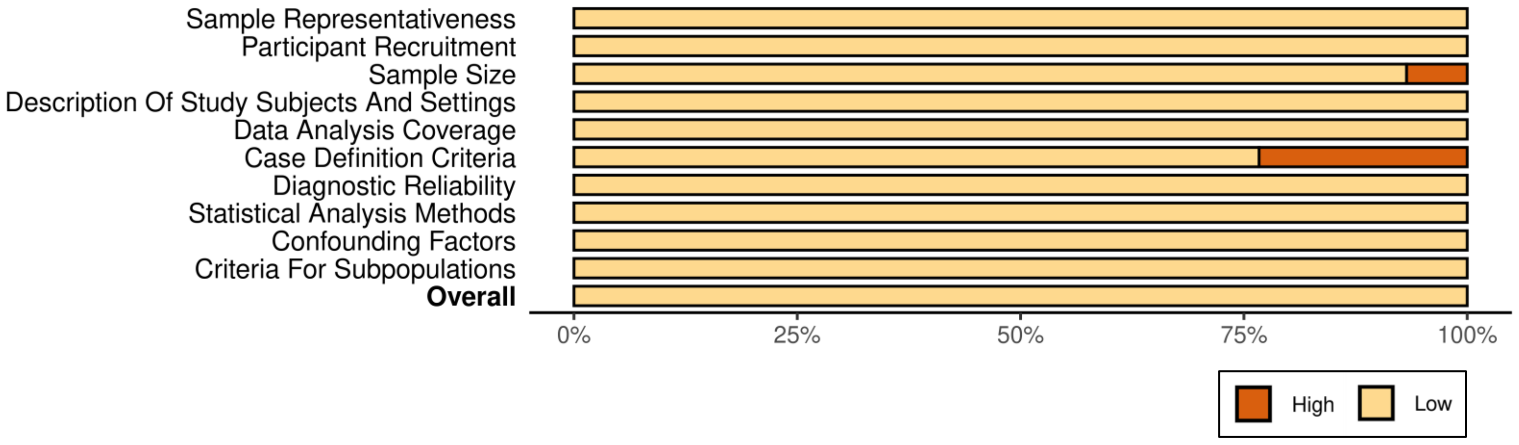

Table S5. Assessment of publication bias using Doi plots and the LFK index.

Outcome type	Outcome measures	LFK index	Doi plot inspection	Interpretation
nHSV incidence rate	140	5.31	Asymmetrical Doi plot	Indicative of potential publication bias
Region of the Americas	80	3.73	Asymmetrical Doi plot	Indicative of potential publication bias
European Region	33	1.96	Asymmetrical Doi plot	Indicative of potential publication bias
Western Pacific Region	27	2.15	Asymmetrical Doi plot	Indicative of potential publication bias
nHSV cases attributable to HSV-1	103	-0.08	Symmetrical Doi plot	No publication bias
nHSV cases attributable to HSV-2	103	0.09	Symmetrical Doi plot	No publication bias

Abbreviations: nHSV, Neonatal herpes simplex virus.

Figure S5. Doi plots for assessing publication bias. Doi plot of studies reporting neonatal herpes A) incidence rate, B) cases attributed to HSV-1, and C) cases attributed to HSV-2.

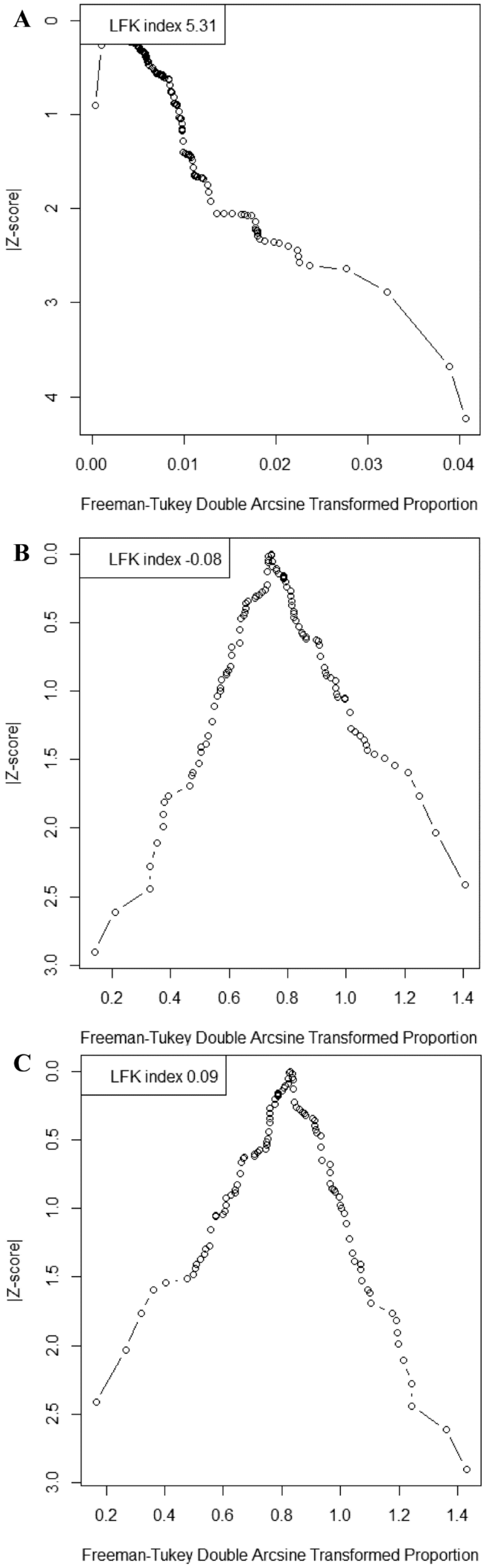

Figure S6. Forest plot for neonatal herpes incidence rate. Forest plot illustrating the global and regional pooled mean incidence rates of neonatal herpes.

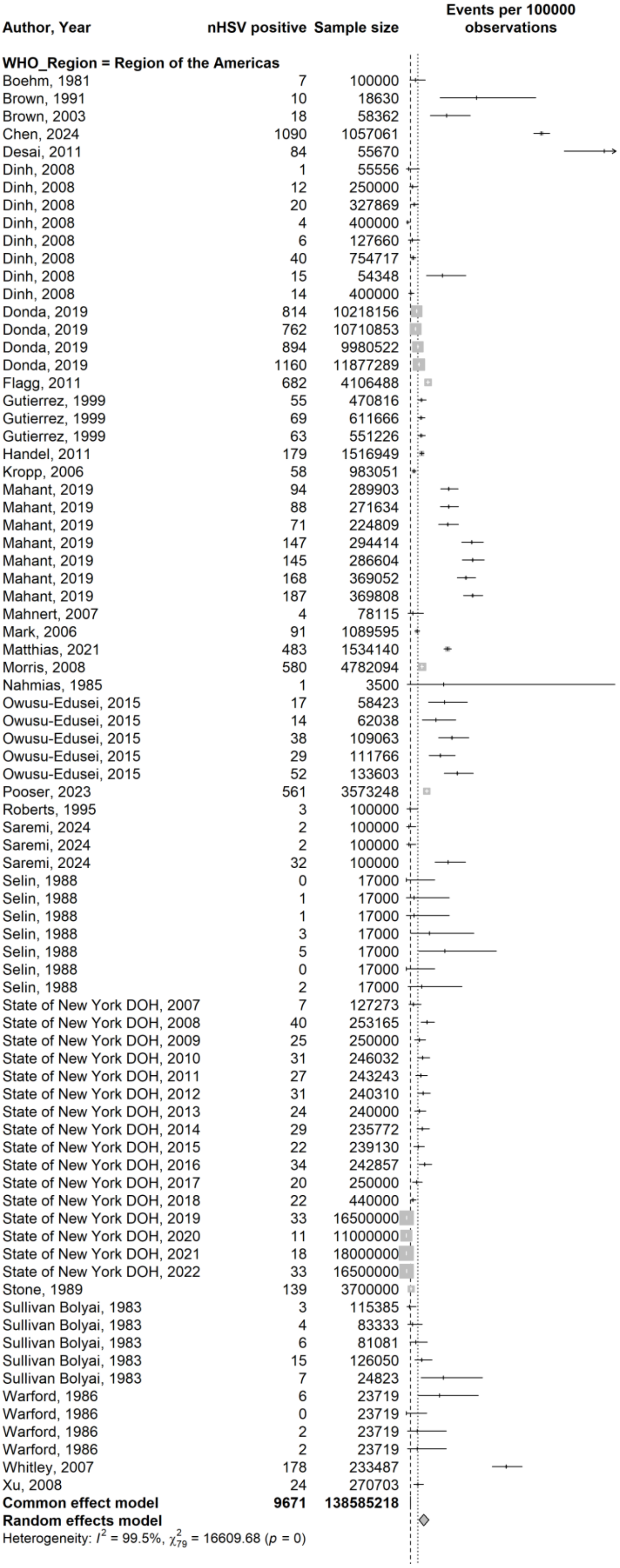

Author, Year	nHSV positive	Sample size	W(Random)	Prev(%)	95%CI
**WHO_Region = Region of the Americas**					
Boehm, 1981	7	100000	0.7%	7.0	[2.8; 14.4]
Brown, 1991	10	18630	0.6%	53.7	[25.7; 98.7]
Brown, 2003	18	58362	0.7%	30.8	[18.3; 48.7]
Chen, 2024	1090	1057061	0.8%	103.1	[97.1; 109.4]
Desai, 2011	84	55670	0.7%	150.9	[120.4; 186.8]
Dinh, 2008	1	55556	0.7%	1.8	[0.0; 10.0]
Dinh, 2008	12	250000	0.7%	4.8	[2.5; 8.4]
Dinh, 2008	20	327869	0.7%	6.1	[3.7; 9.4]
Dinh, 2008	4	400000	0.7%	1.0	[0.3; 2.6]
Dinh, 2008	6	127660	0.7%	4.7	[1.7; 10.2]
Dinh, 2008	40	754717	0.8%	5.3	[3.8; 7.2]
Dinh, 2008	15	54348	0.7%	27.6	[15.4; 45.5]
Dinh, 2008	14	400000	0.7%	3.5	[1.9; 5.9]
Donda, 2019	814	10218156	0.8%	8.0	[7.4; 8.5]
Donda, 2019	762	10710853	0.8%	7.1	[6.6; 7.6]
Donda, 2019	894	9980522	0.8%	9.0	[8.4; 9.6]
Donda, 2019	1160	11877289	0.8%	9.8	[9.2; 10.3]
Flagg, 2011	682	4106488	0.8%	16.6	[15.4; 17.9]
Gutierrez, 1999	55	470816	0.8%	11.7	[8.8; 15.2]
Gutierrez, 1999	69	611666	0.8%	11.3	[8.8; 14.3]
Gutierrez, 1999	63	551226	0.8%	11.4	[8.8; 14.6]
Handel, 2011	179	1516949	0.8%	11.8	[10.1; 13.7]
Kropp, 2006	58	983051	0.8%	5.9	[4.5; 7.6]
Mahant, 2019	94	289903	0.7%	32.4	[26.2; 39.7]
Mahant, 2019	88	271634	0.7%	32.4	[26.0; 39.9]
Mahant, 2019	71	224809	0.7%	31.6	[24.7; 39.8]
Mahant, 2019	147	294414	0.7%	49.9	[42.2; 58.7]
Mahant, 2019	145	286604	0.7%	50.6	[42.7; 59.5]
Mahant, 2019	168	369052	0.7%	45.5	[38.9; 52.9]
Mahant, 2019	187	369808	0.7%	50.6	[43.6; 58.4]
Mahnert, 2007	4	78115	0.7%	5.1	[1.4; 13.1]
Mark, 2006	91	1089595	0.8%	8.4	[6.7; 10.3]
Matthias, 2021	483	1534140	0.8%	31.5	[28.7; 34.4]
Morris, 2008	580	4782094	0.8%	12.1	[11.2; 13.2]
Nahmias, 1985	1	3500	0.3%	28.6	[0.7; 159.1]
Owusu-Edusei, 2015	17	58423	0.7%	29.1	[17.0; 46.6]
Owusu-Edusei, 2015	14	62038	0.7%	22.6	[12.3; 37.9]
Owusu-Edusei, 2015	38	109063	0.7%	34.8	[24.7; 47.8]
Owusu-Edusei, 2015	29	111766	0.7%	25.9	[17.4; 37.3]
Owusu-Edusei, 2015	52	133603	0.7%	38.9	[29.1; 51.0]
Pooser, 2023	561	3573248	0.8%	15.7	[14.4; 17.1]
Roberts, 1995	3	100000	0.7%	3.0	[0.6; 8.8]
Saremi, 2024	2	100000	0.7%	2.0	[0.2; 7.2]
Saremi, 2024	2	100000	0.7%	2.0	[0.2; 7.2]
Saremi, 2024	32	100000	0.7%	32.0	[21.9; 45.2]
Selin, 1988	0	17000	0.5%	0.0	[0.0; 21.7]
Selin, 1988	1	17000	0.5%	5.9	[0.1; 32.8]
Selin, 1988	1	17000	0.5%	5.9	[0.1; 32.8]
Selin, 1988	3	17000	0.5%	17.6	[3.6; 51.6]
Selin, 1988	5	17000	0.5%	29.4	[9.6; 68.6]
Selin, 1988	0	17000	0.5%	0.0	[0.0; 21.7]
Selin, 1988	2	17000	0.5%	11.8	[1.4; 42.5]
State of New York DOH, 2007	7	127273	0.7%	5.5	[2.2; 11.3]
State of New York DOH, 2008	40	253165	0.7%	15.8	[11.3; 21.5]
State of New York DOH, 2009	25	250000	0.7%	10.0	[6.5; 14.8]
State of New York DOH, 2010	31	246032	0.7%	12.6	[8.6; 17.9]
State of New York DOH, 2011	27	243243	0.7%	11.1	[7.3; 16.1]
State of New York DOH, 2012	31	240310	0.7%	12.9	[8.8; 18.3]
State of New York DOH, 2013	24	240000	0.7%	10.0	[6.4; 14.9]
State of New York DOH, 2014	29	235772	0.7%	12.3	[8.2; 17.7]
State of New York DOH, 2015	22	239130	0.7%	9.2	[5.8; 13.9]
State of New York DOH, 2016	34	242857	0.7%	14.0	[9.7; 19.6]
State of New York DOH, 2017	20	250000	0.7%	8.0	[4.9; 12.4]
State of New York DOH, 2018	22	440000	0.8%	5.0	[3.1; 7.6]
State of New York DOH, 2019	33	16500000	0.8%	0.2	[0.1; 0.3]
State of New York DOH, 2020	11	11000000	0.8%	0.1	[0.0; 0.2]
State of New York DOH, 2021	18	18000000	0.8%	0.1	[0.1; 0.2]
State of New York DOH, 2022	33	16500000	0.8%	0.2	[0.1; 0.3]
Stone, 1989	139	3700000	0.8%	3.8	[3.2; 4.4]
Sullivan Bolyai, 1983	3	115385	0.7%	2.6	[0.5; 7.6]
Sullivan Bolyai, 1983	4	83333	0.7%	4.8	[1.3; 12.3]
Sullivan Bolyai, 1983	6	81081	0.7%	7.4	[2.7; 16.1]
Sullivan Bolyai, 1983	15	126050	0.7%	11.9	[6.7; 19.6]
Sullivan Bolyai, 1983	7	24823	0.6%	28.2	[11.3; 58.1]
Warford, 1986	6	23719	0.6%	25.3	[9.3; 55.1]
Warford, 1986	0	23719	0.6%	0.0	[0.0; 15.6]
Warford, 1986	2	23719	0.6%	8.4	[1.0; 30.5]
Warford, 1986	2	23719	0.6%	8.4	[1.0; 30.5]
Whitley, 2007	178	233487	0.7%	76.2	[65.5; 88.3]
Xu, 2008	24	270703	0.7%	8.9	[5.7; 13.2]
**Common effect model**	**9671**	**138585218**	.	**3.2**	**[3.1; 3.3]**
**Random effects model**			**56.2%**	**13.3**	**[9.9; 17.2]**

Heterogeneity: I^2 = 99.5%, χ^2_{79} = 16609.68 (p = 0)

Study	Events	Total	Weight	Rate	95% CI
**WHO_Region = European Region**					
Batra, 2014	10	57291	0.7%	17.5	[8.4; 32.1]
Bouthry, 2024	8	666667	0.8%	1.2	[0.5; 2.4]
Clarke, 2014	76	4560000	0.8%	1.7	[1.3; 2.1]
Clarke, 2014	86	2580000	0.8%	3.3	[2.7; 4.1]
Clarke, 2014	66	688116	0.8%	9.6	[7.4; 12.2]
Dudley, 2023	118	1710145	0.8%	6.9	[5.7; 8.3]
Dungu, 2023	54	596927	0.8%	9.0	[6.8; 11.8]
Engman, 2008	19	267690	0.7%	7.1	[4.3; 11.1]
Fidler, 2020	47	652778	0.8%	7.2	[5.3; 9.6]
Fidler, 2021	59	855072	0.8%	6.9	[5.3; 8.9]
Fonnest, 1997	10	423729	0.7%	2.4	[1.1; 4.3]
Fonnest, 1997	20	438596	0.8%	4.6	[2.8; 7.0]
Forsgren, 1990	12	200000	0.7%	6.0	[3.1; 10.5]
Gaytant, 2000	19	950000	0.8%	2.0	[1.2; 3.1]
Gaytant, 2000	33	1358000	0.8%	2.4	[1.7; 3.4]
Hemelaar, 2015	52	1100399	0.8%	4.7	[3.5; 6.2]
Kadambari, 2020	233	5428864	0.8%	4.3	[3.8; 4.9]
Kadambari, 2020	645	6786080	0.8%	9.5	[8.8; 10.3]
Kadambari, 2020	10	1111111	0.8%	0.9	[0.4; 1.7]
Kidszun, 2022	37	1574468	0.8%	2.4	[1.7; 3.2]
Kohelet, 2004	11	6953	0.4%	158.2	[79.0; 282.9]
Koren, 2013	22	261488	0.7%	8.4	[5.3; 12.7]
Koskiniemi, 1989	79	240000	0.7%	32.9	[26.1; 41.0]
Kucera, 2012	0	6507	0.4%	0.0	[0.0; 56.7]
Lagziel, 2024	75	1272509	0.8%	5.9	[4.6; 7.4]
Lecumberri Garcia, 2015	7	78400	0.7%	8.9	[3.6; 18.4]
Pascual, 2011	7	437500	0.8%	1.6	[0.6; 3.3]
Poeran, 2008	33	1031250	0.8%	3.2	[2.2; 4.5]
Sachane, 2017	8	67500	0.7%	11.9	[5.1; 23.4]
Tookey, 1996	76	4606061	0.8%	1.6	[1.3; 2.1]
Tookey, 2020	85	2374302	0.8%	3.6	[2.9; 4.4]
van Everdingen, 1993	26	910000	0.8%	2.9	[1.9; 4.2]
van Oeffelen, 2018	33	687500	0.8%	4.8	[3.3; 6.7]
**Common effect model**	**2076**	**43985903**	**.**	**3.7**	**[3.5; 3.8]**
**Random effects model**			**24.0%**	**5.2**	**[3.4; 7.3]**
Heterogeneity: $I^2 = 96.6\%$, $\chi^2_{32} = 954.12$ ($p < 0.0001$)					
**WHO_Region = Western Pacific Region**					
Berkhout, 2021	76	791667	0.8%	9.6	[7.6; 12.0]
Deverell, 2012	8	296296	0.7%	2.7	[1.2; 5.3]
Deverell, 2013	9	302013	0.7%	3.0	[1.4; 5.7]
Deverell, 2014	14	309735	0.7%	4.5	[2.5; 7.6]
Deverell, 2016	10	307692	0.7%	3.3	[1.6; 6.0]
Deverell, 2017a	16	299625	0.7%	5.3	[3.1; 8.7]
Deverell, 2017b	6	306122	0.7%	2.0	[0.7; 4.3]
Elder, 1995	5	37440	0.6%	13.4	[4.3; 31.2]
Garland, 1992	6	66000	0.7%	9.1	[3.3; 19.8]
Jones, 2010	98	2816092	0.8%	3.5	[2.8; 4.2]
Mindel, 2000	1	13372	0.5%	7.5	[0.2; 41.7]
Morishima, 2000	16	1600000	0.8%	1.0	[0.6; 1.6]
Morishima, 2000	24	1500000	0.8%	1.6	[1.0; 2.4]
Morishima, 2000	34	1619048	0.8%	2.1	[1.5; 2.9]
Morishima, 2000	39	1444444	0.8%	2.7	[1.9; 3.7]
Morishima, 2000	43	1303030	0.8%	3.3	[2.4; 4.4]
Morishima, 2000	45	1216216	0.8%	3.7	[2.7; 5.0]
Teutsch, 2018	4	303030	0.7%	1.3	[0.4; 3.4]
Teutsch, 2020	8	303030	0.7%	2.6	[1.1; 5.2]
Teutsch, 2021	8	305344	0.7%	2.6	[1.1; 5.2]
Teutsch, 2022	14	305677	0.7%	4.6	[2.5; 7.7]
Teutsch, 2023	10	295858	0.7%	3.4	[1.6; 6.2]
Torii, 2013	38	1461538	0.8%	2.6	[1.8; 3.6]
Yamada, 2015	7	788673	0.8%	0.9	[0.4; 1.8]
Zurynski, 2010	9	300000	0.7%	3.0	[1.4; 5.7]
Zurynski, 2010	10	303030	0.7%	3.3	[1.6; 6.1]
Zurynski, 2011	4	307692	0.7%	1.3	[0.4; 3.3]
**Common effect model**	**562**	**18902664**	**.**	**2.5**	**[2.3; 2.8]**
**Random effects model**			**19.9%**	**2.9**	**[2.2; 3.6]**
Heterogeneity: $I^2 = 84\%$, $\chi^2_{26} = 162.59$ ($p < 0.0001$)					
**Common effect model**	**12309**	**201473785**	**.**	**3.3**	**[3.2; 3.4]**
**Random effects model**			**100.0%**	**8.8**	**[6.9; 10.9]**
**Prediction interval**					**[0.0; 46.8]**
Heterogeneity: $I^2 = 99.2\%$, $\chi^2_{139} = 17805.70$ ($p = 0$)					

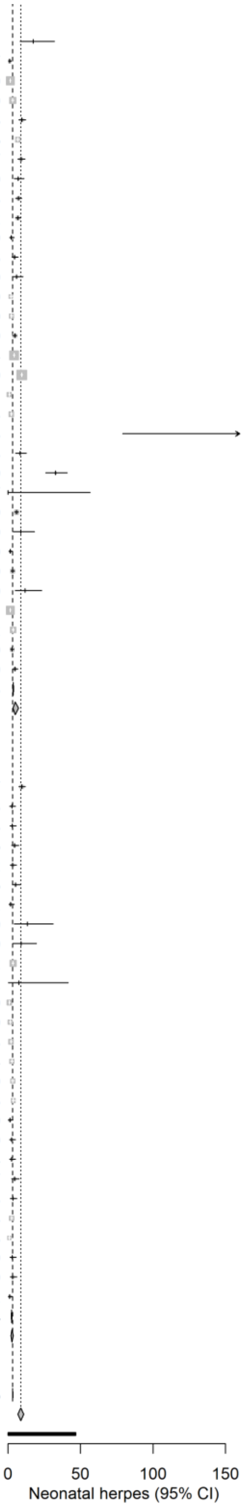

Abbreviations: CI, Confidence interval; nHSV, Neonatal herpes simplex virus.

Table S6. Leave-one-out sensitivity analysis. Leave-one-out analysis of pooled mean estimates for the incidence rate of neonatal herpes**.**

Study	Pooled mean neonatal herpes incidence rate (per 100,000 live births) (95% CI)	Absolute difference
Omitting Desai, 2011	8.4 (6.7-10.3)	0.431
Omitting Chen, 2024	8.4 (6.7-10.4)	0.370
Omitting Whitley, 2007	8.5 (6.7-10.6)	0.282
Omitting Mahant, 2019.6	8.6 (6.7-10.7)	0.199
Omitting Mahant, 2019.4	8.6 (6.7-10.7)	0.198
Omitting Kohelet, 2004	8.6 (6.8-10.7)	0.198
Omitting Mahant, 2019.3	8.6 (6.7-10.7)	0.196
Omitting Mahant, 2019.5	8.6 (6.8-10.7)	0.181
Omitting Owusu-Edusei, 2015.4	8.7 (6.8-10.8)	0.149
Omitting Brown, 1991	8.7 (6.8-10.8)	0.144
Omitting Owusu-Edusei, 2015.2	8.7 (6.8-10.8)	0.131
Omitting Koskiniemi, 1989	8.7 (6.8-10.8)	0.128
Omitting Mahant, 2019	8.7 (6.8-10.8)	0.127
Omitting Mahant, 2019.1	8.7 (6.8-10.8)	0.126
Omitting Matthias, 2021	8.7 (6.8-10.8)	0.125
Omitting State of New York DOH, 2021	8.9 (7.0-11.1)	0.123
Omitting State of New York DOH, 2020	8.9 (7.0-11.1)	0.123
Omitting Mahant, 2019.2	8.7 (6.8-10.8)	0.122
Omitting Saremi, 2024.2	8.7 (6.8-10.8)	0.119
Omitting State of New York DOH, 2019	8.9 (7.0-11.1)	0.118
Omitting State of New York DOH, 2022	8.9 (7.0-11.1)	0.118
Omitting Brown, 2003	8.7 (6.8-10.8)	0.108
Omitting Owusu-Edusei, 2015	8.7 (6.8-10.8)	0.101
Omitting Kadambari, 2020.2	8.9 (7.0-11.1)	0.094
Omitting Yamada, 2015	8.9 (7.0-11.1)	0.094
Omitting Dinh, 2008.6	8.7 (6.8-10.8)	0.094
Omitting Owusu-Edusei, 2015.3	8.7 (6.8-10.8)	0.093
Omitting Morishima, 2000	8.9 (7.0-11.1)	0.092
Omitting Dinh, 2008.3	8.9 (7.0-11.1)	0.090
Omitting Bouthry, 2024	8.9 (7.0-11.0)	0.087
Omitting Warford, 1986.1	8.9 (7.0-11.0)	0.086
Omitting Zurynski, 2011	8.9 (7.0-11.0)	0.083
Omitting Teutsch, 2018	8.9 (7.0-11.0)	0.083
Omitting Morishima, 2000.1	8.9 (7.0-11.0)	0.081
Omitting Sullivan Bolyai, 1983.4	8.7 (6.8-10.9)	0.081
Omitting Tookey, 1996	8.9 (7.0-11.0)	0.080
Omitting Clarke, 2014	8.9 (7.0-11.0)	0.080
Omitting Selin, 1988	8.9 (7.0-11.0)	0.079
Omitting Selin, 1988.5	8.9 (7.0-11.0)	0.079
Omitting Pascual, 2011	8.9 (7.0-11.0)	0.079
Omitting Kucera, 2012	8.9 (7.0-11.0)	0.076
Omitting Gaytant, 2000	8.9 (7.0-11.0)	0.074
Omitting Owusu-Edusei, 2015.1	8.7 (6.8-10.9)	0.073
Omitting Morishima, 2000.2	8.9 (7.0-11.0)	0.073
Omitting Selin, 1988.4	8.7 (6.8-10.9)	0.072
Omitting Deverell, 2017b	8.9 (7.0-11.0)	0.072
Omitting Warford, 1986	8.7 (6.8-10.9)	0.069
Omitting Kidszun, 2022	8.9 (7.0-11.0)	0.069
Omitting Gaytant, 2000.1	8.9 (7.0-11.0)	0.068
Omitting Fonnest, 1997	8.9 (7.0-11.0)	0.067
Omitting Saremi, 2024	8.9 (7.0-11.0)	0.066
Omitting Saremi, 2024.1	8.9 (7.0-11.0)	0.066
Omitting Torii, 2013	8.9 (7.0-11.0)	0.065
Omitting Dinh, 2008	8.9 (7.0-11.0)	0.064
Omitting Morishima, 2000.3	8.9 (7.0-11.0)	0.064
Omitting Teutsch, 2021	8.9 (7.0-11.0)	0.063
Omitting Teutsch, 2020	8.9 (7.0-11.0)	0.063
Omitting Deverell, 2012	8.9 (7.0-11.0)	0.062
Omitting van Everdingen, 1993	8.9 (6.9-11.0)	0.061
Omitting Sullivan Bolyai, 1983	8.9 (6.9-11.0)	0.060
Omitting Deverell, 2013	8.9 (6.9-11.0)	0.058

Omitting Zurynski, 2010	8.9 (6.9-11.0)	0.058
Omitting Poeran, 2008	8.9 (6.9-11.0)	0.057
Omitting Morishima, 2000.4	8.9 (6.9-11.0)	0.056
Omitting Clarke, 2014.1	8.9 (6.9-11.0)	0.056
Omitting Deverell, 2016	8.9 (6.9-11.0)	0.055
Omitting Zurynski, 2010.1	8.9 (6.9-11.0)	0.054
Omitting Roberts, 1995	8.9 (6.9-11.0)	0.054
Omitting Jones, 2010	8.9 (6.9-11.0)	0.054
Omitting Teutsch, 2023	8.9 (6.9-11.0)	0.053
Omitting Tookey, 2020	8.9 (6.9-11.0)	0.053
Omitting Dinh, 2008.7	8.9 (6.9-11.0)	0.052
Omitting Morishima, 2000.5	8.9 (6.9-11.0)	0.051
Omitting Stone, 1989	8.9 (6.9-11.0)	0.051
Omitting Flagg, 2011	8.8 (6.8-10.9)	0.050
Omitting Batra, 2014	8.8 (6.9-10.9)	0.048
Omitting Kadambari, 2020	8.9 (6.9-11.0)	0.045
Omitting Pooser, 2023	8.8 (6.9-10.9)	0.044
Omitting State of New York DOH, 2008	8.8 (6.9-10.9)	0.044
Omitting Nahmias, 1985	8.9 (6.9-11.0)	0.041
Omitting Fonnest, 1997.1	8.9 (6.9-11.0)	0.041
Omitting Deverell, 2014	8.9 (6.9-11.0)	0.041
Omitting Teutsch, 2022	8.9 (6.9-11.0)	0.040
Omitting Hemelaar, 2015	8.9 (6.9-11.0)	0.040
Omitting van Oeffelen, 2018	8.9 (6.9-11.0)	0.039
Omitting Dinh, 2008.1	8.9 (6.9-11.0)	0.038
Omitting Dinh, 2008.4	8.9 (6.9-11.0)	0.037
Omitting State of New York DOH, 2018	8.9 (6.9-11.0)	0.036
Omitting Sullivan Bolyai, 1983.1	8.9 (6.9-11.0)	0.035
Omitting Dinh, 2008.5	8.8 (6.9-11.0)	0.034
Omitting State of New York DOH, 2016	8.8 (6.9-10.9)	0.033
Omitting Deverell, 2017a	8.8 (6.9-11.0)	0.033
Omitting Selin, 1988.3	8.8 (6.9-10.9)	0.032
Omitting Mahnert, 2007	8.8 (6.9-11.0)	0.032
Omitting State of New York DOH, 2007	8.8 (6.9-11.0)	0.030
Omitting Lagziel, 2024	8.8 (6.9-11.0)	0.028
Omitting Kropp, 2006	8.8 (6.9-11.0)	0.028
Omitting State of New York DOH, 2012	8.8 (6.9-10.9)	0.026
Omitting Forsgren, 1990	8.8 (6.9-11.0)	0.026
Omitting Dinh, 2008.2	8.8 (6.9-11.0)	0.025
Omitting State of New York DOH, 2010	8.8 (6.9-10.9)	0.024
Omitting Elder, 1995	8.8 (6.9-10.9)	0.023
Omitting Selin, 1988.1	8.8 (6.9-11.0)	0.023
Omitting Selin, 1988.2	8.8 (6.9-11.0)	0.023
Omitting State of New York DOH, 2014	8.8 (6.9-10.9)	0.022
Omitting Morris, 2008	8.8 (6.9-10.9)	0.021
Omitting Handel, 2011	8.8 (6.9-10.9)	0.019
Omitting Dudley, 2023	8.8 (6.9-11.0)	0.019
Omitting Fidler, 2021	8.8 (6.9-11.0)	0.019
Omitting Sullivan Bolyai, 1983.3	8.8 (6.9-10.9)	0.019
Omitting Gutierrez, 1999	8.8 (6.9-10.9)	0.018
Omitting Sachane, 2017	8.8 (6.9-10.9)	0.017
Omitting Donda, 2019.1	8.8 (6.9-11.0)	0.017
Omitting Engman, 2008	8.8 (6.9-11.0)	0.017
Omitting Boehm, 1981	8.8 (6.9-11.0)	0.016
Omitting Gutierrez, 1999.2	8.8 (6.9-10.9)	0.016
Omitting Mindel, 2000	8.8 (6.9-11.0)	0.016
Omitting Fidler, 2020	8.8 (6.9-11.0)	0.016
Omitting Gutierrez, 1999.1	8.8 (6.9-10.9)	0.015
Omitting State of New York DOH, 2011	8.8 (6.9-10.9)	0.014
Omitting Sullivan Bolyai, 1983.2	8.8 (6.9-11.0)	0.013
Omitting Donda, 2019	8.8 (6.9-11.0)	0.010
Omitting State of New York DOH, 2017	8.8 (6.9-11.0)	0.009
Omitting Selin, 1988.6	8.8 (6.9-10.9)	0.007
Omitting Mark, 2006	8.8 (6.9-11.0)	0.007
Omitting Warford, 1986.2	8.8 (6.9-11.0)	0.007
Omitting Warford, 1986.3	8.8 (6.9-11.0)	0.007
Omitting State of New York DOH, 2009	8.8 (6.9-11.0)	0.006

Omitting State of New York DOH, 2013	8.8 (6.9-11.0)	0.006
Omitting Koren, 2013	8.8 (6.9-11.0)	0.006
Omitting Donda, 2019.3	8.8 (6.9-11.0)	0.004
Omitting Berkhout, 2021	8.8 (6.9-11.0)	0.003
Omitting Clarke, 2014.2	8.8 (6.9-11.0)	0.003
Omitting Xu, 2008	8.8 (6.9-11.0)	0.002
Omitting Kadambari, 2020.1	8.8 (6.9-11.0)	0.002
Omitting Donda, 2019.2	8.8 (6.9-11.0)	0.002
Omitting Lecumberri Garcia, 2015	8.8 (6.9-11.0)	0.002
Omitting Dungu, 2023	8.8 (6.9-11.0)	0.001
Omitting Garland, 1992	8.8 (6.9-11.0)	0.001
Omitting State of New York DOH, 2015	8.8 (6.9-11.0)	0.000

Abbreviations: CI, Confidence interval; DOH, Department of Health.

Table S7. Sensitivity analyses on the meta-regressions. Sensitivity analyses on the main meta-regression analyses for the incidence rate of neonatal herpes.

			Outcome measures	Number of live births	Univariable analyses				Multivariable analyses			
									Model 1		Model 2	
			Total n	Total N	Crude IRR (95% CI)	p-value	LR test p-value	Adjusted R^2	Adjusted IRR (95% CI)	p-value	Adjusted IRR (95% CI)	p-value
Population characteristics	WHO Region	Region of the Americas	80	138,585,218	1.00	-	<0.001	14.2	1.00	-	1.00	-
		European Region	33	43,985,903	0.50 (0.30-0.82)	0.006			0.54 (0.30-0.99)	0.046	0.51 (0.28-0.91)	0.024
		Western Pacific Region	27	18,902,664	0.30 (0.18-0.52)	<0.001			0.49 (0.25-0.99)	0.046	0.45 (0.23-0.90)	0.023
	Country[a]	United States	72	137,483,167	1.00	-	0.005	14.4	-	-	-	-
		Australia	19	7,969,715	0.36 (0.20-0.68)	0.002			-	-	-	-
		Canada	8	1,102,051	1.04 (0.34-3.17)	0.943			-	-	-	-
		Denmark	3	1,459,252	0.46 (0.11-1.83)	0.268			-	-	-	-
		Israel	3	1,540,950	1.89 (0.47-7.56)	0.363			-	-	-	-
		Japan	8	10,932,949	0.20 (0.08-0.48)	<0.001			-	-	-	-
		Netherlands	6	6,037,149	0.31 (0.12-0.84)	0.022			-	-	-	-
		United Kingdom	13	31,477,320	0.48 (0.23-0.96)	0.039			-	-	-	-
		Other countries[b]	8	3,471,232	0.48 (0.19-1.22)	0.120			-	-	-	-
Methodological characteristics	Site type	National surveillance registry/report	65	116,238,593	1.00	-	<0.001	26.4	1.00	-	1.00	-
		Clinical setting	35	9,049,159	3.17 (1.96-5.13)	<0.001			3.59 (1.94-6.63)	<0.001	4.09 (2.23-7.53)	<0.001
		Healthcare database	24	70,439,796	5.23 (3.12-8.78)	<0.001			3.24 (1.69-6.22)	0.001	3.26 (1.72-6.17)	<0.001
		Virological laboratory	4	2,069,357	1.41 (0.45-4.43)	0.556			2.11 (0.64-6.89)	0.215	2.17 (0.69-6.79)	0.184
		Mixed/Unclear	12	3,676,880	2.14 (0.97-4.74)	0.061			2.60 (1.09-6.20)	0.031	2.86 (1.23-6.65)	0.015
	Estimate type	National estimate	68	118,353,291	1.00	-	0.132	1.7	1.00	-	1.00	-
		Regional estimate	57	81,882934	1.27 (0.80-2.01)	0.311			1.05 (0.57-1.96)	0.866	1.09 (0.59-1.99)	0.789
		Institutional estimate	15	1,237,560	2.21 (0.99-4.95)	0.053			0.95 (0.38-2.37)	0.918	0.91 (0.37-2.21)	0.829
	Risk of bias: case definition criteria domain[c]	Low risk of bias	97	170,786,533	1.00	-	0.864	0.0	1.00	-	1.00	-
		High risk of bias	43	30,687,252	1.04 (0.64-1.70)	0.864			1.48 (0.93-2.36)	0.098	1.59 (1.01-2.51)	0.044
	Risk of bias: diagnostic reliability domain[c]	Low risk of bias	126	187,312,290	1.00	-	<0.001	9.6	1.00	-	1.00	-
		High risk of bias	14	14,161,495	3.93 (1.93-7.96)	<0.001			1.95 (0.98-3.87)	0.057	1.90 (0.97-3.70)	0.060
Temporal trends[d]	Year of data collection category	<1995	43	29,290,482	1.00	-	0.850	0.0	1.00	-	-	-
		1995-2004	23	31,919,950	1.19 (0.60-2.36)	0.607			1.48 (0.80-2.75)	0.209	-	-
		≥2005	74	140,263,353	1.13 (0.68-1.88)	0.642			1.75 (1.04-2.96)	0.036	-	-
	Year of data collection		140	201,473,785	1.00 (0.98-1.02)	0.710	0.710	0.0	-	-	1.03 (1.01-1.05)	0.003

Abbreviations: CI, Confidence interval; IRR, Incidence rate ratio; LR test, Likelihood ratio test; WHO, World Health Organization.
Adjusted R^2 in the final multivariable model 1 is 33.8%.
Adjusted R^2 in the final multivariable model 2 is 36.7%.
[a]The country variable was excluded from the multivariable model due to collinearity with the region variable.
[b]This category includes countries with two or fewer studies including Finland, France, Germany, Spain, Sweden, and Switzerland.
[c]Of the 10 risk of bias domains assessed in this systematic review, 8 were rated as low risk of bias across all studies and were therefore excluded from the meta-regression analyses. The only domains with variability were the "case definition criteria" domain and the "diagnostic reliability" domain.
[d]To assess temporal trends, the year of data collection was included in the multivariable models irrespective of the p-value criteria for model inclusion.

Figure S7. Forest plot of HSV-1 contribution to neonatal herpes. Forest plot illustrating the global and regional pooled mean proportion of neonatal herpes cases attributed to HSV-1.

Author, Year	HSV-1 positive	Sample size	W(Random)	Prev(%)	95%CI
**WHO_Region = Region of the Americas**					
Boehm, 1981	2	7	0.7%	28.6	[3.7; 71.0]
Brown, 1991	3	10	0.8%	30.0	[6.7; 65.2]
Brown, 2003	8	18	1.0%	44.4	[21.5; 69.2]
Brown, 2007	10	25	1.0%	40.0	[21.1; 61.3]
Burchett, 1992	7	22	1.0%	31.8	[13.9; 54.9]
Cantey, 2012	10	21	1.0%	47.6	[25.7; 70.2]
Caviness, 2008a	11	40	1.1%	27.5	[14.6; 43.9]
Caviness, 2008b	4	10	0.8%	40.0	[12.2; 73.8]
CDC, 2012	37	70	1.2%	52.9	[40.6; 64.9]
Ch'ien, 1975	4	11	0.9%	36.4	[10.9; 69.2]
Corey, 1988	14	33	1.1%	42.4	[25.5; 60.8]
Cruz, 2018	53	96	1.2%	55.2	[44.7; 65.4]
Dendi, 2020	39	88	1.2%	44.3	[33.7; 55.3]
Dominguez, 2018	4	7	0.7%	57.1	[18.4; 90.1]
Ericson, 2022	16	44	1.1%	36.4	[22.4; 52.2]
Graf, 20204	13	15	0.9%	86.7	[59.5; 98.3]
Handel, 2011	28	55	1.2%	50.9	[37.1; 64.6]
Kimberlin, 2001	20	74	1.2%	27.0	[17.4; 38.6]
Kimberlin, 2011	17	53	1.2%	32.1	[19.9; 46.3]
Kotzbauer, 2014	6	13	0.9%	46.2	[19.2; 74.9]
Kropp, 2006	30	48	1.2%	62.5	[47.4; 76.0]
Lao, 2019	43	93	1.2%	46.2	[35.8; 56.9]
Long, 2011	2	17	1.0%	11.8	[1.5; 36.4]
Mahnert, 2007	1	3	0.5%	33.3	[0.8; 90.6]
Matthias, 2021	73	164	1.2%	44.5	[36.8; 52.5]
Mejias, 2023	7	12	0.9%	58.3	[27.7; 84.8]
Melvin, 2022	15	43	1.1%	34.9	[21.0; 50.9]
Melvin, 2022	13	29	1.1%	44.8	[26.4; 64.3]
Melvin, 2022	17	38	1.1%	44.7	[28.6; 61.7]
Melvin, 2022	12	21	1.0%	57.1	[34.0; 78.2]
Nahmias, 1969	3	25	1.0%	12.0	[2.5; 31.2]
O'Riordan, 2006	0	12	0.9%	0.0	[0.0; 26.5]
Pass, 1981	2	11	0.9%	18.2	[2.3; 51.8]
Samies, 2019	12	18	1.0%	66.7	[41.0; 86.7]
Schillinger, 2013	40	76	1.2%	52.6	[40.8; 64.2]
Selin, 1988	5	11	0.9%	45.5	[16.7; 76.6]
Stone, 1989	34	105	1.2%	32.4	[23.6; 42.2]
Sullender, 1987	16	55	1.2%	29.1	[17.6; 42.9]
Sullivan Bolyai, 1983	6	27	1.1%	22.2	[8.6; 42.3]
Whitley, 1988	21	80	1.2%	26.2	[17.0; 37.3]
Whitley, 1991	65	184	1.3%	35.3	[28.4; 42.7]
Xu, 2008	7	17	1.0%	41.2	[18.4; 67.1]
Yeager, 1980	3	11	0.9%	27.3	[6.0; 61.0]
Yeager, 1984	10	19	1.0%	52.6	[28.9; 75.6]
**Common effect model**	**743**	**1831**	**.**	**39.9**	**[37.6; 42.3]**
**Random effects model**			**45.2%**	**39.6**	**[35.0; 44.3]**

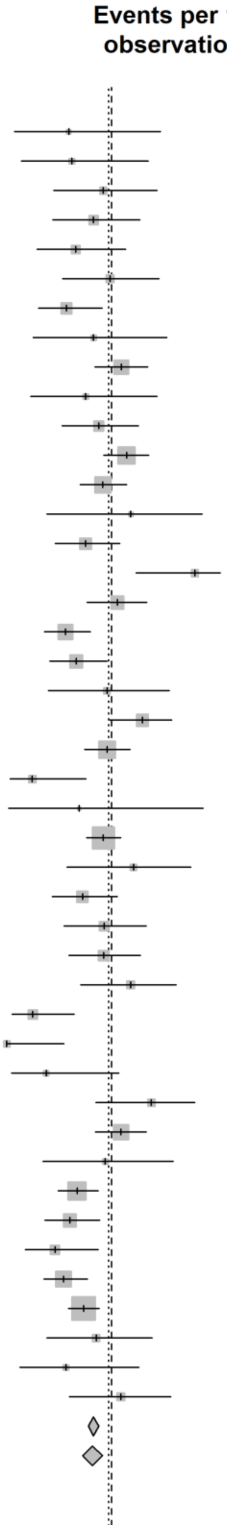

Heterogeneity: $I^2 = 65.3\%$, $\chi^2_{43} = 123.96$ ($p < 0.0001$)

**WHO_Region = European Region**				
Batra, 2014	9	17	1.0%	52.9 [27.8; 77.0]
Bouthry, 2024	4	8	0.8%	50.0 [15.7; 84.3]
Brown, 2007	10	35	1.1%	28.6 [14.6; 46.3]
Dudley, 2023	29	78	1.2%	37.2 [26.5; 48.9]
Dungu, 2023	52	53	1.2%	98.1 [89.9; 100.0]
Engman, 2008	2	9	0.8%	22.2 [2.8; 60.0]
Fidler, 2021	29	54	1.2%	53.7 [39.6; 67.4]
Fonnest, 1997	3	7	0.7%	42.9 [9.9; 81.6]
Fonnest, 1997	1	2	0.4%	50.0 [1.3; 98.7]
Forsgren, 1990	12	48	1.2%	25.0 [13.6; 39.6]
Gaytant, 2000	24	27	1.1%	88.9 [70.8; 97.6]
Hemelaar, 2015	33	35	1.1%	94.3 [80.8; 99.3]
Kashyap, 2014	6	11	0.9%	54.5 [23.4; 83.3]
Keunig, 2019	2	6	0.7%	33.3 [4.3; 77.7]
Khandwalla, 2021	1	10	0.8%	10.0 [0.3; 44.5]
Kidszun, 2022	20	24	1.0%	83.3 [62.6; 95.3]
Kohelet, 2004	0	5	0.6%	0.0 [0.0; 52.2]
Koren, 2013	13	17	1.0%	76.5 [50.1; 93.2]
Koskiniemi, 1989	1	13	0.9%	7.7 [0.2; 36.0]
Lagziel, 2024	77	85	1.2%	90.6 [82.3; 95.8]
Leventon-Kriss, 1983	1	3	0.5%	33.3 [0.8; 90.6]
Lewensohn-Fuchs, 2003	3	28	1.1%	10.7 [2.3; 28.2]
Malm, 1991	10	45	1.1%	22.2 [11.2; 37.1]
Malm, 1999	7	36	1.1%	19.4 [8.2; 36.0]
Pascual, 2011	4	6	0.7%	66.7 [22.3; 95.7]
Poeran, 2008	15	28	1.1%	53.6 [33.9; 72.5]
Tookey, 1996	25	49	1.2%	51.0 [36.3; 65.6]
Tookey, 2007	11	24	1.0%	45.8 [25.6; 67.2]
Tookey, 2007	15	31	1.1%	48.4 [30.2; 66.9]
Tookey, 2007	9	24	1.0%	37.5 [18.8; 59.4]
van Everdingen, 1993	15	22	1.0%	68.2 [45.1; 86.1]
van Everdingen, 1993	12	16	1.0%	75.0 [47.6; 92.7]
van Oeffelen, 2018	18	23	1.0%	78.3 [56.3; 92.5]
Vaughan, 2024	3	4	0.6%	75.0 [19.4; 99.4]
Waheed, 2022	6	20	1.0%	30.0 [11.9; 54.3]
**Common effect model**	**482**	**903**	**.**	**55.1 [51.7; 58.6]**
**Random effects model**			**33.2%**	**50.4 [39.6; 61.2]**

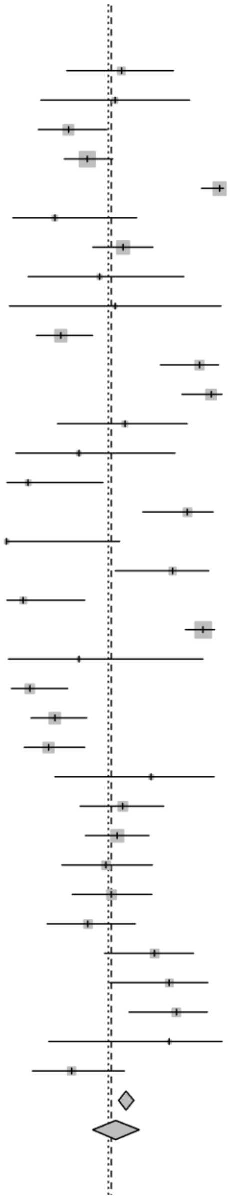

Heterogeneity: $I^2 = 90.3\%$, $\chi^2_{34} = 349.08$ ($p < 0.0001$)

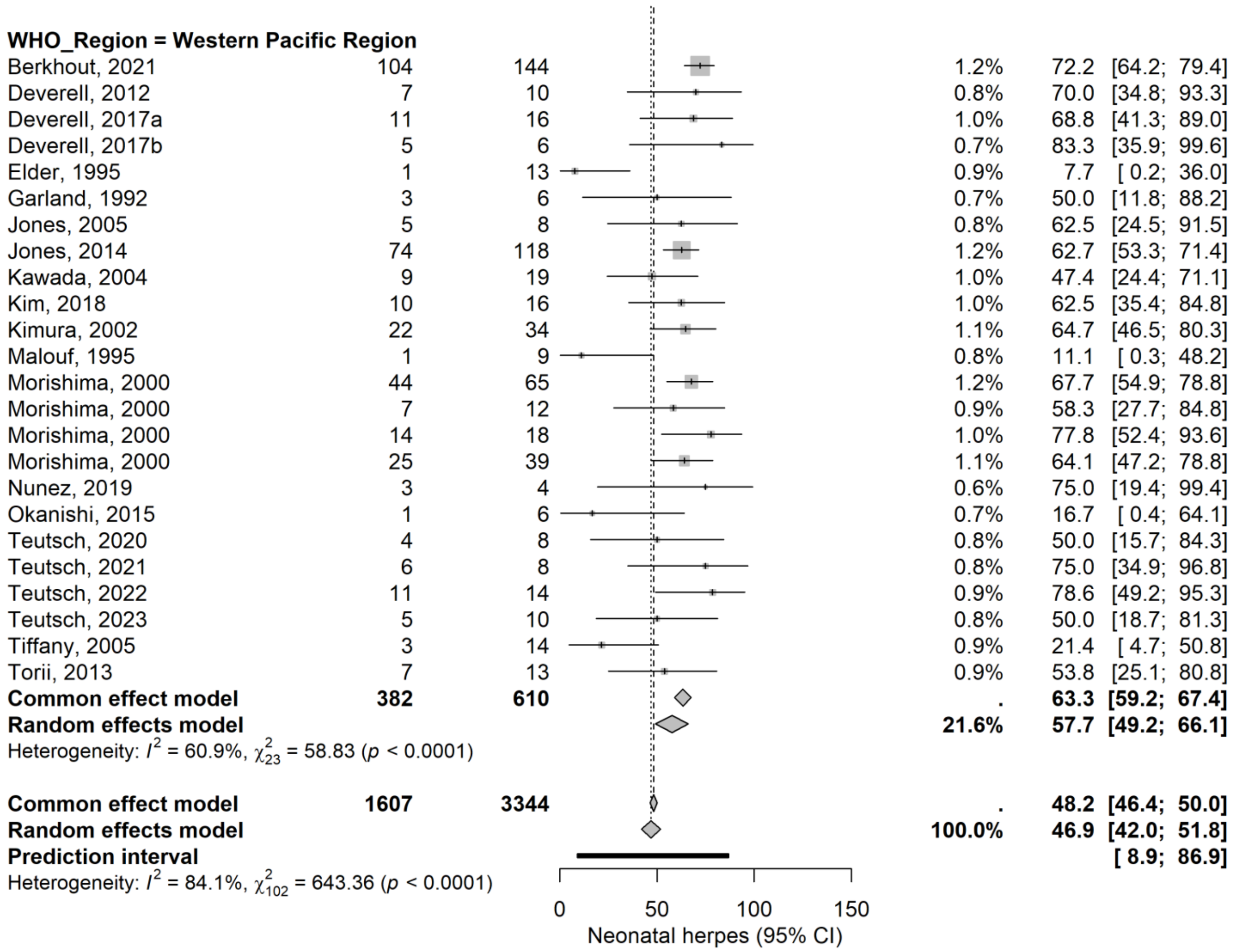

Abbreviations: CI, Confidence interval; HSV-1, Herpes simplex virus type 1.

Figure S8. Forest plot of HSV-2 contribution to neonatal herpes. Forest plot illustrating the global and regional pooled mean proportion of neonatal herpes cases attributed to HSV-2.

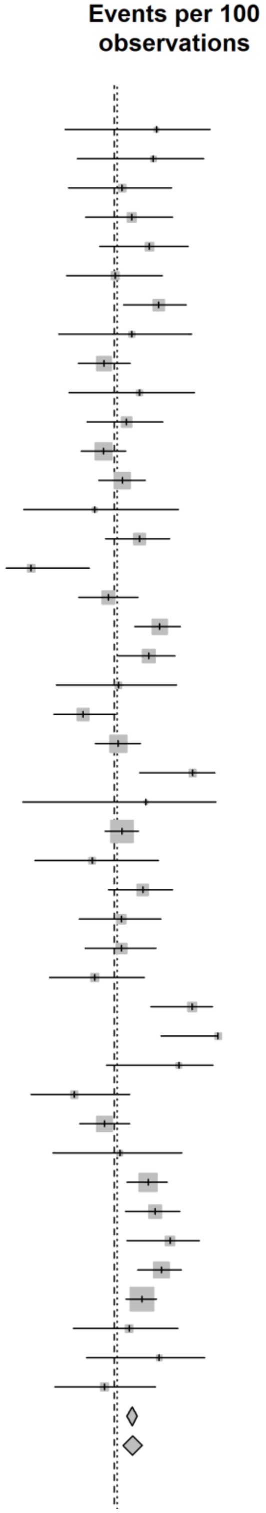

Author, Year	HSV-2 positive	Sample size	W(Random)	Prev(%)	95%CI
**WHO_Region = Region of the Americas**					
Boehm, 1981	5	7	0.7%	71.4	[29.0; 96.3]
Brown, 1991	7	10	0.8%	70.0	[34.8; 93.3]
Brown, 2003	10	18	1.0%	55.6	[30.8; 78.5]
Brown, 2007	15	25	1.1%	60.0	[38.7; 78.9]
Burchett, 1992	15	22	1.0%	68.2	[45.1; 86.1]
Cantey, 2012	11	21	1.0%	52.4	[29.8; 74.3]
Caviness, 2008a	29	40	1.1%	72.5	[56.1; 85.4]
Caviness, 2008b	6	10	0.8%	60.0	[26.2; 87.8]
CDC, 2012	33	70	1.2%	47.1	[35.1; 59.4]
Ch'ien, 1975	7	11	0.9%	63.6	[30.8; 89.1]
Corey, 1988	19	33	1.1%	57.6	[39.2; 74.5]
Cruz, 2018	45	96	1.2%	46.9	[36.6; 57.3]
Dendi, 2020	49	88	1.2%	55.7	[44.7; 66.3]
Dominguez, 2018	3	7	0.7%	42.9	[9.9; 81.6]
Ericson, 2022	28	44	1.1%	63.6	[47.8; 77.6]
Graf, 20204	2	15	0.9%	13.3	[1.7; 40.5]
Handel, 2011	27	55	1.2%	49.1	[35.4; 62.9]
Kimberlin, 2001	54	74	1.2%	73.0	[61.4; 82.6]
Kimberlin, 2011	36	53	1.2%	67.9	[53.7; 80.1]
Kotzbauer, 2014	7	13	0.9%	53.8	[25.1; 80.8]
Kropp, 2006	18	48	1.2%	37.5	[24.0; 52.6]
Lao, 2019	50	93	1.2%	53.8	[43.1; 64.2]
Long, 2011	15	17	1.0%	88.2	[63.6; 98.5]
Mahnert, 2007	2	3	0.5%	66.7	[9.4; 99.2]
Matthias, 2021	91	164	1.3%	55.5	[47.5; 63.2]
Mejias, 2023	5	12	0.9%	41.7	[15.2; 72.3]
Melvin, 2022	28	43	1.1%	65.1	[49.1; 79.0]
Melvin, 2022	16	29	1.1%	55.2	[35.7; 73.6]
Melvin, 2022	21	38	1.1%	55.3	[38.3; 71.4]
Melvin, 2022	9	21	1.0%	42.9	[21.8; 66.0]
Nahmias, 1969	22	25	1.1%	88.0	[68.8; 97.5]
O'Riordan, 2006	12	12	0.9%	100.0	[73.5; 100.0]
Pass, 1981	9	11	0.9%	81.8	[48.2; 97.7]
Samies, 2019	6	18	1.0%	33.3	[13.3; 59.0]
Schillinger, 2013	36	76	1.2%	47.4	[35.8; 59.2]
Selin, 1988	6	11	0.9%	54.5	[23.4; 83.3]
Stone, 1989	71	105	1.2%	67.6	[57.8; 76.4]
Sullender, 1987	39	55	1.2%	70.9	[57.1; 82.4]
Sullivan Bolyai, 1983	21	27	1.1%	77.8	[57.7; 91.4]
Whitley, 1988	59	80	1.2%	73.8	[62.7; 83.0]
Whitley, 1991	119	184	1.3%	64.7	[57.3; 71.6]
Xu, 2008	10	17	1.0%	58.8	[32.9; 81.6]
Yeager, 1980	8	11	0.9%	72.7	[39.0; 94.0]
Yeager, 1984	9	19	1.0%	47.4	[24.4; 71.1]
**Common effect model**	**1090**	**1831**	**.**	**60.2**	**[57.8; 62.5]**
**Random effects model**			**45.3%**	**60.5**	**[55.8; 65.1]**

Heterogeneity: $I^2 = 64.7\%$, $\chi^2_{43} = 121.76$ ($p < 0.0001$)

**WHO_Region = European Region**					
Batra, 2014	8	17	1.0%	47.1	[23.0; 72.2]
Bouthry, 2024	4	8	0.8%	50.0	[15.7; 84.3]
Brown, 2007	25	35	1.1%	71.4	[53.7; 85.4]
Dudley, 2023	49	78	1.2%	62.8	[51.1; 73.5]
Dungu, 2023	1	53	1.2%	1.9	[0.0; 10.1]
Engman, 2008	7	9	0.8%	77.8	[40.0; 97.2]
Fidler, 2021	25	54	1.2%	46.3	[32.6; 60.4]
Fonnest, 1997	4	7	0.7%	57.1	[18.4; 90.1]
Fonnest, 1997	1	2	0.4%	50.0	[1.3; 98.7]
Forsgren, 1990	36	48	1.2%	75.0	[60.4; 86.4]
Gaytant, 2000	3	27	1.1%	11.1	[2.4; 29.2]
Hemelaar, 2015	2	35	1.1%	5.7	[0.7; 19.2]
Kashyap, 2014	5	11	0.9%	45.5	[16.7; 76.6]
Keunig, 2019	4	6	0.7%	66.7	[22.3; 95.7]
Khandwalla, 2021	9	10	0.8%	90.0	[55.5; 99.7]
Kidszun, 2022	6	24	1.0%	25.0	[9.8; 46.7]
Kohelet, 2004	5	5	0.6%	100.0	[47.8; 100.0]
Koren, 2013	4	17	1.0%	23.5	[6.8; 49.9]
Koskiniemi, 1989	12	13	0.9%	92.3	[64.0; 99.8]
Lagziel, 2024	8	85	1.2%	9.4	[4.2; 17.7]
Leventon-Kriss, 1983	2	3	0.5%	66.7	[9.4; 99.2]
Lewensohn-Fuchs, 2003	25	28	1.1%	89.3	[71.8; 97.7]
Malm, 1991	35	45	1.1%	77.8	[62.9; 88.8]
Malm, 1999	29	36	1.1%	80.6	[64.0; 91.8]
Pascual, 2011	2	6	0.7%	33.3	[4.3; 77.7]
Poeran, 2008	13	28	1.1%	46.4	[27.5; 66.1]
Tookey, 1996	24	49	1.2%	49.0	[34.4; 63.7]
Tookey, 2007	13	24	1.0%	54.2	[32.8; 74.4]
Tookey, 2007	16	31	1.1%	51.6	[33.1; 69.8]
Tookey, 2007	15	24	1.0%	62.5	[40.6; 81.2]
van Everdingen, 1993	7	22	1.0%	31.8	[13.9; 54.9]
van Everdingen, 1993	4	16	1.0%	25.0	[7.3; 52.4]
van Oeffelen, 2018	5	23	1.0%	21.7	[7.5; 43.7]
Vaughan, 2024	1	4	0.6%	25.0	[0.6; 80.6]
Waheed, 2022	14	20	1.0%	70.0	[45.7; 88.1]
**Common effect model**	**423**	**903**	**.**	**45.1**	**[41.7; 48.6]**
**Random effects model**			**33.2%**	**49.9**	**[39.2; 60.6]**

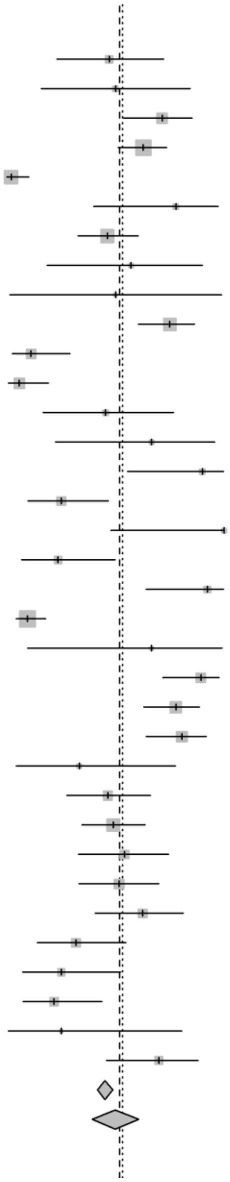

Heterogeneity: $I^2 = 90.1\%$, $\chi^2_{34} = 344.26$ ($p < 0.0001$)

WHO_Region = Western Pacific Region

Berkhout, 2021	40	144	1.2%	27.8	[20.6; 35.8]
Deverell, 2012	3	10	0.8%	30.0	[6.7; 65.2]
Deverell, 2017a	5	16	1.0%	31.2	[11.0; 58.7]
Deverell, 2017b	1	6	0.7%	16.7	[0.4; 64.1]
Elder, 1995	12	13	0.9%	92.3	[64.0; 99.8]
Garland, 1992	3	6	0.7%	50.0	[11.8; 88.2]
Jones, 2005	3	8	0.8%	37.5	[8.5; 75.5]
Jones, 2014	44	118	1.2%	37.3	[28.6; 46.7]
Kawada, 2004	10	19	1.0%	52.6	[28.9; 75.6]
Kim, 2018	6	16	1.0%	37.5	[15.2; 64.6]
Kimura, 2002	12	34	1.1%	35.3	[19.7; 53.5]
Malouf, 1995	8	9	0.8%	88.9	[51.8; 99.7]
Morishima, 2000	21	65	1.2%	32.3	[21.2; 45.1]
Morishima, 2000	5	12	0.9%	41.7	[15.2; 72.3]
Morishima, 2000	4	18	1.0%	22.2	[6.4; 47.6]
Morishima, 2000	14	39	1.1%	35.9	[21.2; 52.8]
Nunez, 2019	1	4	0.6%	25.0	[0.6; 80.6]
Okanishi, 2015	5	6	0.7%	83.3	[35.9; 99.6]
Teutsch, 2020	4	8	0.8%	50.0	[15.7; 84.3]
Teutsch, 2021	2	8	0.8%	25.0	[3.2; 65.1]
Teutsch, 2022	3	14	0.9%	21.4	[4.7; 50.8]
Teutsch, 2023	5	10	0.8%	50.0	[18.7; 81.3]
Tiffany, 2005	11	14	0.9%	78.6	[49.2; 95.3]
Torii, 2013	6	13	0.9%	46.2	[19.2; 74.9]
**Common effect model**	**228**	**610**	**.**	**36.7**	**[32.6; 40.8]**
**Random effects model**			**21.6%**	**42.3**	**[33.9; 50.8]**
Heterogeneity: I^2 = 60.9%, χ^2_{23} = 58.83 (p < 0.0001)					
**Common effect model**	**1741**	**3344**	**.**	**51.9**	**[50.1; 53.7]**
**Random effects model**			**100.0%**	**53.2**	**[48.4; 58.1]**
**Prediction interval**					**[13.4; 90.9]**
Heterogeneity: I^2 = 84.0%, χ^2_{102} = 636.38 (p < 0.0001)					

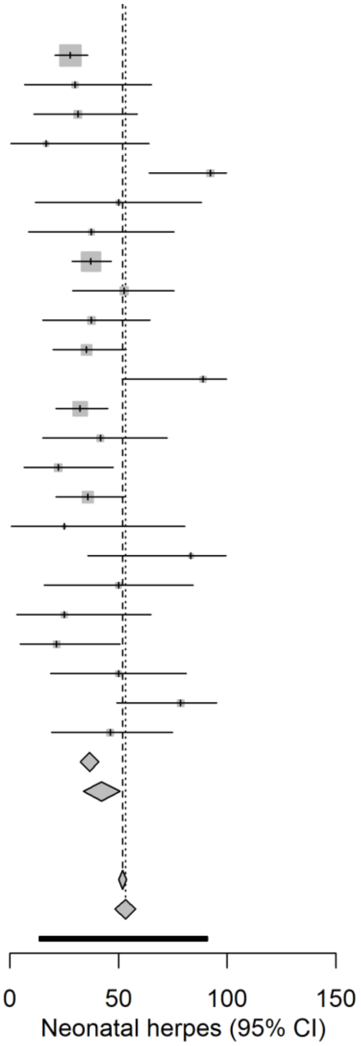

Abbreviations: CI, Confidence interval; HSV-2, Herpes simplex virus type 2.

References

1 Moher D, Liberati A, Tetzlaff J, Altman DG, Group P. Preferred reporting items for systematic reviews and meta-analyses: the PRISMA statement. PLoS medicine. 2009;6:e1000097.

2 Page MJ, McKenzie JE, Bossuyt PM, Boutron I, Hoffmann TC, Mulrow CD, et al. The PRISMA 2020 statement: an updated guideline for reporting systematic reviews. BMJ. 2021;372:n71.

3 World Health Organization. Alphabetical List of WHO Member States Available at https://www.who.int/countries; Accessed on: December 12, 2024.

4 Aromataris E, Lockwood C, Porritt K, Pilla B, Jordan Z. JBI Manual for Evidence Synthesis Available from: https://synthesismanual.jbi.global. Accessed on: April 2, 2024. 2024.

5 Munn Z, Moola S, Lisy K, Riitano D, Tufanaru C. Methodological guidance for systematic reviews of observational epidemiological studies reporting prevalence and cumulative incidence data. Int J Evid Based Healthc. 2015;13:147-53.

6 Munn Z, Moola S, Riitano D, Lisy K. The development of a critical appraisal tool for use in systematic reviews addressing questions of prevalence. Int J Health Policy Manag. 2014;3:123-8.

7 Kropp RY, Wong T, Cormier L, Ringrose A, Burton S, Embree JE, et al. Neonatal herpes simplex virus infections in Canada: results of a 3-year national prospective study. Pediatrics. 2006;117:1955-62.

8 Selin LK, Hammond GW, Aoki FY. Neonatal herpes simplex virus infection in Manitoba, 1980 to 1986, and implications for preventive strategies. Pediatric Infectious Disease Journal. 1988;7(10):733-4.

9 Boehm FH, Estes W, Wright PF, Growdon JF, Jr. Management of genital herpes simplex virus infection occurring during pregnancy. Am J Obstet Gynecol. 1981;141:735-40.

10 Brown ZA, Benedetti J, Ashley R, Burchett S, Selke S, Berry S, et al. Neonatal herpes simplex virus infection in relation to asymptomatic maternal infection at the time of labor. N Engl J Med. 1991;324:1247-52.

11 Brown ZA, Wald A, Morrow RA, Selke S, Zeh J, Corey L. Effect of serologic status and cesarean delivery on transmission rates of herpes simplex virus from mother to infant. JAMA. 2003;289:203-9.

12 Chen N, Kilpatrick R, VerHage EJ, Smith PB, Bukhari A, Hornik CD, et al. Epidemiology and treatment of herpes simplex virus in the neonatal intensive care unit. J Perinatol. 2024.

13 Desai N, Schillinger J, Bratu S, Eramo A, Bowers C, Agrawal A. Investigating a cluster of neonatal herpes at a single institution. Sexually Transmitted Infections. 2011;1):A107.

14 Dinh TH, Dunne EF, Markowitz LE, Weinstock H, Berman S. Assessing neonatal herpes reporting in the United States, 2000-2005. Sex Transm Dis. 2008;35:19-21.

15 Donda K, Sharma M, Amponsah JK, Bhatt P, Okaikoi M, Chaudhari R, et al. Trends in the incidence, mortality, and cost of neonatal herpes simplex virus hospitalizations in the United States from 2003 to 2014. J Perinatol. 2019;39:697-707.

16 Flagg EW, Weinstock H. Incidence of neonatal herpes simplex virus infections in the United States, 2006. Pediatrics. 2011;127:e1-8.

17 Gutierrez KM, Falkovitz Halpern MS, Maldonado Y, Arvin AM. The epidemiology of neonatal herpes simplex virus infections in California from 1985 to 1995. J Infect Dis. 1999;180:199-202.
18 Handel S, Klingler EJ, Washburn K, Blank S, Schillinger JA. Population-based surveillance for neonatal herpes in New York City, April 2006-September 2010. Sex Transm Dis. 2011;38:705-11.
19 Mahant S, Hall M, Schondelmeyer AC, Berry JG, Kimberlin DW, Shah SS. Neonatal Herpes Simplex Virus Infection Among Medicaid-Enrolled Children: 2009-2015. Pediatrics. 2019;143.
20 Mahnert N, Roberts SW, Laibl VR, Sheffield JS, Wendel GD, Jr. The incidence of neonatal herpes infection. Am J Obstet Gynecol. 2007;196:e55-6.
21 Mark KE, Kim HN, Wald A, Gardella C, Reed SD. Targeted prenatal herpes simplex virus testing: can we identify women at risk of transmission to the neonate? Am J Obstet Gynecol. 2006;194:408-14.
22 Matthias J, du Bernard S, Schillinger JA, Hong J, Pearson V, Peterman TA. Estimating Neonatal Herpes Simplex Virus Incidence and Mortality Using Capture-recapture, Florida. Clin Infect Dis. 2021;73:506-12.
23 Morris SR, Bauer HM, Samuel MC, Gallagher D, Bolan G. Neonatal herpes morbidity and mortality in California, 1995-2003. Sex Transm Dis. 2008;35:14-8.
24 Nahmias AJ, Schwahn MG. Neonatal herpes simplex: a worldwide disease which is potentially preventable and treatable. Progress in clinical and biological research. 1985;163 B:355-62.
25 Owusu-Edusei K, Jr., Flagg EW, Gift TL. Hospitalization cost per case of neonatal herpes simplex virus infection from claims data. J Pediatr Nurs. 2015;30:346-52.
26 Pooser M, Yuan Y, Karki S, O'Callaghan KP, Hufstetler K, Kreisel K. Estimated Incidence, Mortality, and Costs of Neonatal Herpes Simplex Virus Infections in the United States, 2019. Obstetrics and Gynecology. 2024;143:48S.
27 Roberts SW, Cox SM, Dax J, Wendel GD, Jr., Leveno KJ. Genital herpes during pregnancy: no lesions, no cesarean. Obstet Gynecol. 1995;85:261-4.
28 Saremi N, Lewis KA, Klausner JD. Neonatal Herpes Reporting in the United States: Review of Current Policies by State. J Pediatric Infect Dis Soc. 2024;13:297-9.
29 State of New York DOH. 2007 Communicable Disease Annual Reports. Available at https://www.health.ny.gov/statistics/diseases/communicable/ 2007.
30 State of New York DOH. 2008 Communicable Disease Annual Reports. Available at https://www.health.ny.gov/statistics/diseases/communicable/ 2008.
31 State of New York DOH. 2009 Communicable Disease Annual Reports. Available at https://www.health.ny.gov/statistics/diseases/communicable/ 2009.
32 State of New York DOH. 2010 Communicable Disease Annual Reports. Available at https://www.health.ny.gov/statistics/diseases/communicable/ 2010.
33 State of New York DOH. 2011 Communicable Disease Annual Reports. Available at https://www.health.ny.gov/statistics/diseases/communicable/ 2011.
34 State of New York DOH. 2012 Communicable Disease Annual Reports. Available at https://www.health.ny.gov/statistics/diseases/communicable/ 2012.
35 State of New York DOH. 2013 Communicable Disease Annual Reports. Available at https://www.health.ny.gov/statistics/diseases/communicable/ 2013.

36 State of New York DOH. 2014 Communicable Disease Annual Reports. Available at https://www.health.ny.gov/statistics/diseases/communicable/ 2014.
37 State of New York DOH. 2015 Communicable Disease Annual Reports. Available at https://www.health.ny.gov/statistics/diseases/communicable/ 2015.
38 State of New York DOH. 2016 Communicable Disease Annual Reports. Available at https://www.health.ny.gov/statistics/diseases/communicable/ 2016.
39 State of New York DOH. 2017 Communicable Disease Annual Reports. Available at https://www.health.ny.gov/statistics/diseases/communicable/ 2017.
40 State of New York DOH. 2018 Communicable Disease Annual Reports. Available at https://www.health.ny.gov/statistics/diseases/communicable/ 2018.
41 State of New York DOH. 2019 Communicable Disease Annual Reports. Available at https://www.health.ny.gov/statistics/diseases/communicable/ 2019.
42 State of New York DOH. 2020 Communicable Disease Annual Reports. Available at https://www.health.ny.gov/statistics/diseases/communicable/ 2020.
43 State of New York DOH. 2021 Communicable Disease Annual Reports. Available at https://www.health.ny.gov/statistics/diseases/communicable/ 2021.
44 State of New York DOH. 2022 Communicable Disease Annual Reports. Available at https://www.health.ny.gov/statistics/diseases/communicable/ 2022.
45 Stone KM, Brooks CA, Guinan ME, Alexander ER. National surveillance for neonatal herpes simplex virus infections. Sex Transm Dis. 1989;16:152-6.
46 Sullivan Bolyai J, Hull HF, Wilson C, Corey L. Neonatal herpes simplex virus infection in King County, Washington. Increasing incidence and epidemiologic correlates. Journal of the American Medical Association. 1983;250(22):3059-62.
47 Warford AL, Levy RA, Rekrut KA, Steinberg E. Herpes simplex virus testing of an obstetric population with an antigen enzyme-linked immunosorbent assay. Am J Obstet Gynecol. 1986;154:21-8.
48 Whitley R, Davis EA, Suppapanya N. Incidence of neonatal herpes simplex virus infections in a managed-care population. Sex Transm Dis. 2007;34:704-8.
49 Xu F, Gee JM, Naleway A, Zangwill KM, Ackerson B, Eriksen E, et al. Incidence of neonatal herpes simplex virus infections in two managed care organizations: implications for surveillance. Sex Transm Dis. 2008;35:592-8.
50 Dungu KHS, Lund S, Malchau Carlsen EL, Hartling UB, Matthesen AT, Franck KT, et al. Herpes simplex virus infection among neonates suspected of invasive bacterial infection: a population-based cohort study. Arch Dis Child Fetal Neonatal Ed. 2023.
51 Fonnest G, de la Fuente Fonnest I, Weber T. Neonatal herpes in Denmark 1977-1991. Acta Obstet Gynecol Scand. 1997;76:355-8.
52 Koskiniemi M, Happonen JM, Järvenpää AL, Pettay O, Vaheri A. Neonatal herpes simplex virus infection: a report of 43 patients. Pediatr Infect Dis J. 1989;8:30-5.
53 Bouthry E, Portet-Sulla V, Bouokazi MM, Perillaud-Dubois C, Javaugue FC, Jule L, et al. Neonatal herpes: case series in two obstetric centres over a 10-year period (2013-2023), France. Eur J Pediatr. 2024;183:3183-91.
54 Kidszun A, Bruns A, Schreiner D, Tippmann S, Winter J, Pokora RM, et al. Characteristics of neonatal herpes simplex virus infections in Germany: results of a 2-year prospective nationwide surveillance study. Arch Dis Child Fetal Neonatal Ed. 2022;107:188-92.

55 Lagziel TG, Jurkowicz M, Gordon O, Mor M, Megged O, Nasrallah E, et al. Israeli neonatal herpes simplex infection: Unique epidemiology and clinical profile. J Med Virol. 2024;96:e29934.
56 Kohelet D, Katz N, Sadan O, Somekh E. Herpes simplex virus infection after vacuum-assisted vaginally delivered infants of asymptomatic mothers. J Perinatol. 2004;24:147-9.
57 Koren A, Tasher D, Stein M, Yossepowitch O, Somekh E. Neonatal herpes simplex virus infections in Israel. Pediatr Infect Dis J. 2013;32:120-3.
58 Gaytant MA, Steegers EAP, Van Cromvoirt PLM, Semmekrot BA, Galama JMD. The incidence of neonatal herpes in the Netherlands. [Dutch]. Nederlands Tijdschrift voor Geneeskunde. 2000;144(38):1832-6.
59 Hemelaar SJ, Poeran J, Steegers EA, van der Meijden WI. Neonatal herpes infections in The Netherlands in the period 2006-2011. J Matern Fetal Neonatal Med. 2015;28:905-9.
60 Poeran J, Wildschut H, Gaytant M, Galama J, Steegers E, van der Meijden W. The incidence of neonatal herpes in The Netherlands. J Clin Virol. 2008;42:321-5.
61 van Everdingen JJ, Peeters MF, ten Have P. Neonatal herpes policy in The Netherlands. Five years after a consensus conference. J Perinat Med. 1993;21:371-5.
62 van Oeffelen L, Biekram M, Poeran J, Hukkelhoven C, Galjaard S, van der Meijden W, et al. Update on Neonatal Herpes Simplex Epidemiology in the Netherlands: A Health Problem of Increasing Concern? Pediatr Infect Dis J. 2018;37:806-13.
63 Lecumberri Garcia N, Lavilla Oiz A, Armendariz Cuevas Y, Sierra Colomina G, Torrus Carmona S, Goni Orayen C. Herpes simplex virus in our neonatal unit. Journal of Perinatal Medicine Conference: 12th World Congress of Perinatal Medicine. 2015;43.
64 Engman ML, Adolfsson I, Lewensohn-Fuchs I, Forsgren M, Mosskin M, Malm G. Neuropsychologic outcomes in children with neonatal herpes encephalitis. Pediatr Neurol. 2008;38:398-405.
65 Forsgren M. Genital herpes simplex virus infection and incidence of neonatal disease in Sweden. Scand J Infect Dis Suppl. 1990;69:37-41.
66 Kucera P, Gerber S, Marques-Vidal P, Meylan PR. Seroepidemiology of herpes simplex virus type 1 and 2 in pregnant women in Switzerland: an obstetric clinic based study. Eur J Obstet Gynecol Reprod Biol. 2012;160:13-7.
67 Pascual A, Moessinger A, Gerber S, Meylan P, Swiss Paediatric Surveillance U. Neonatal herpes simplex virus infections in Switzerland: results of a 6-year national prospective surveillance study. Clin Microbiol Infect. 2011;17:1907-10.
68 Dudley J, Heath P, Yan G, Fidler K. 912 Herpes simplex virus in infants under 90 days of age in UK and Ireland: results of the 2019–2022 BPSU study. BMJ Publishing Group Ltd; 2023.
69 Fidler K. British Paediatric Surveillance Unit Annual report, 2019-2020. British Paediatric Surveillance Unit: Royal College of Paediatrics and Child Health: 2020.
70 Fidler K, Heath P, Dudley J, Yan G, Lynn R. Neonatal hsv disease in infants under 90 days of age in uk and ireland: BPSU study interim analysis. Archives of Disease in Childhood. 2021;106(SUPPL 1):A390-A1.
71 Tookey P, Peckham CS. Neonatal herpes simplex virus infection in the British Isles. Paediatr Perinat Epidemiol. 1996;10:432-42.
72 Tookey PA, Mahdavi S, Peckham CS. Surveillance of neonatal herpes in the British Isles 2004-2006. F1000Research. 2020;9 (no pagination).

73 Batra D, Davies P, Manktelow BN, Smith C. The incidence and presentation of neonatal herpes in a single UK tertiary centre, 2006-2013. Arch Dis Child. 2014;99:916-21.
74 Clarke E, Shone E, Wright C, Patel R. Neonatal herpes simplex virus infection-is the UK special anymore? Sexually Transmitted Diseases. 2014;1):S60.
75 Kadambari S, Pollard AJ, Goldacre MJ, Goldacre R. Congenital viral infections in England over five decades: a population-based observational study. Lancet Infect Dis. 2020;20:220-9.
76 Sachane K, Ironton R, Pelosi E. Neonatal HSV-experience over a decade in a tertiary neonatal unit in UK. Archives of Disease in Childhood. 2017;102(Supplement 1):A189.
77 Berkhout A, Kapoor V, Heney C, Jones CA, Clark JE, Vaska VL, et al. Herpes Simplex Virus Infection in Infants: 13 Year Evaluation (2005-2017) of Laboratory Confirmed Cases in Queensland, Australia. Pediatr Infect Dis J. 2021;40:209-14.
78 Deverell M, Zurynski Y, Elliott E, chief investigators of Ass. Australian Paediatric Surveillance Unit annual report, 2011. Communicable diseases intelligence quarterly report. 2012;36(3):E263-7.
79 Deverell M, Zurynski Y, Elliott E, all chief investigators of Ass. Australian Paediatric Surveillance Unit annual report, 2012. Commun Dis Intell Q Rep. 2013;37:E394-7.
80 Deverell M, Zurynski YA, Elliott EJ. Australian Paediatric Surveillance Unit annual report, 2013. Commun Dis Intell Q Rep. 2014;38:E343-2.
81 Deverell M, Zurynski Y, Elliott E, all chief investigators of Ass. Australian Paediatric Surveillance Unit annual report, 2014. Commun Dis Intell Q Rep. 2016;40:E216-20.
82 Deverell M, Phu A, Zurynski YA, Elliott EJ. Australian Paediatric Surveillance Unit annual report, 2015. Communicable diseases intelligence quarterly report. 2017;41(2):E181-E5.
83 Deverell M, Phu A, Zurynski Y, Elliott E. Australian Paediatric Surveillance Unit Annual Report, 2016. Commun Dis Intell Q Rep. 2017;41:E288-e93.
84 Elder DE. Neonatal herpes simplex infection: Keys to early diagnosis. Journal of Paediatrics and Child Health. 1995;31(4):307-11.
85 Garland SM. Neonatal herpes simplex: Royal Women's Hospital 10-year experience with management guidelines for herpes in pregnancy. Aust N Z J Obstet Gynaecol. 1992;32:331-4.
86 Jones C, Isaacs D, McIntyre P, Cunningham T, Garland S. Australian Paediatric Surveillance Unit, Biannual Research Report 2007- 2008. 2010.
87 Mindel A, Taylor J, Tideman RL, Seifert C, Berry G, Wagner K, et al. Neonatal herpes prevention: a minor public health problem in some communities. Sex Transm Infect. 2000;76:287-91.
88 Teutsch S, Zurynski Y, Elliott E, chief investigators of Ass. Australian Paediatric Surveillance Unit Annual Report, 2017. Commun Dis Intell (2018). 2018;42.
89 Teutsch SM, Nunez CA, Morris A, McGregor S, King J, Brotherton JM, et al. Australian Paediatric Surveillance Unit (APSU) Annual Surveillance Report 2019. Commun Dis Intell (2018). 2020;44.
90 Teutsch SM, Nunez CA, Morris A, Eslick GD, Khandaker G, Berkhout A, et al. Australian Paediatric Surveillance Unit (APSU) Annual Surveillance Report 2020. Commun Dis Intell (2018). 2021;45.

91 Teutsch SM, Nunez CA, Morris A, Eslick GD, Berkhout A, Novakovic D, et al. Australian Paediatric Surveillance Unit (APSU) Annual Surveillance Report 2021. Commun Dis Intell (2018). 2022;46.

92 Teutsch SM, Nunez CA, Morris A, Eslick GD, Elliott EJ. Australian Paediatric Surveillance Unit (APSU) Annual Surveillance Report 2022. Commun Dis Intell (2018). 2023;47.

93 Zurynski Y, Davey E, Elliott EJ. Australian Paediatric Surveillance Unit annual report, 2008 and 2009. Commun Dis Intell Q Rep. 2010;34:285-90.

94 Zurynski Y, Elliott EJ. Australian Paediatric Surveillance Unit annual report, 2010. Commun Dis Intell Q Rep. 2011;35:244-9.

95 Morishima T. Clinical statistics on alpha-herpesvirus in Japan. [Japanese]. Nippon rinsho. 2000;Japanese journal of clinical medicine. 58(4):845-50.

96 Torii Y, Kimura H, Ito Y, Hayakawa M, Tanaka T, Tajiri H, et al. Clinicoepidemiologic status of mother-to-child infections: A nationwide survey in Japan. Pediatric Infectious Disease Journal. 2013;32(6):699-701.

97 Yamada H, Tairaku S, Morioka I, Sonoyama A, Tanimura K, Deguchi M, et al. Nationwide survey of mother-to-child infections in Japan. J Infect Chemother. 2015;21:161-4.

98 Brown EL, Gardella C, Malm G, Prober CG, Forsgren M, Krantz EM, et al. Effect of maternal herpes simplex virus (HSV) serostatus and HSV type on risk of neonatal herpes. Acta Obstet Gynecol Scand. 2007;86:523-9.

99 Burchett SK, Corey L, Mohan KM, Westall J, Ashley R, Wilson CB. Diminished interferon-gamma and lymphocyte proliferation in neonatal and postpartum primary herpes simplex virus infection. J Infect Dis. 1992;165:813-8.

100 Cantey JB, Mejías A, Wallihan R, Doern C, Brock E, Salamon D, et al. Use of blood polymerase chain reaction testing for diagnosis of herpes simplex virus infection. J Pediatr. 2012;161:357-61.

101 Caviness AC, Demmler GJ, Selwyn BJ. Clinical and laboratory features of neonatal herpes simplex virus infection: a case-control study. Pediatr Infect Dis J. 2008;27:425-30.

102 Caviness AC, Demmler GJ, Almendarez Y, Selwyn BJ. The prevalence of neonatal herpes simplex virus infection compared with serious bacterial illness in hospitalized neonates. J Pediatr. 2008;153:164-9.

103 Centers for Disease C, Prevention. Neonatal herpes simplex virus infection following Jewish ritual circumcisions that included direct orogenital suction - New York City, 2000-2011. Mmwr. 2012;Morbidity and mortality weekly report. 61(22):405-9.

104 Ch'ien LT, Whitley RJ, Nahmias AJ, Lewin EB, Linnemann CC, Jr., Frenkel LD, et al. Antiviral chemotherapy and neonatal herpes simplex virus infecition: a pilot study--experience with adenine arabinoside (ARA-A). Pediatrics. 1975;55:678-85.

105 Corey L, Stone EF, Whitley RJ, Mohan K. Differrence between herpes simplex virus type 1 and type 2 neonatal encephalitis in neurological outcome. Lancet. 1988;1(8575-8576):1-4.

106 Dendi A, Garcia IVH, Mejias A, Hanlon CT, Sanchez PJ. Neonatal Herpes Simplex Virus (HSV) Infection: Is It the Only Pathogen? Open Forum Infectious Diseases. 2020;7(SUPPL 1):S712-S3.

107 Dominguez SR, Pretty K, Hengartner R, Robinson CC. Comparison of herpes simplex virus PCR with culture for virus detection in multisource surface swab specimens from neonates. Journal of Clinical Microbiology. 2018;56(10) (no pagination).
108 Ericson JE, Benjamin DK, Jr., Boakye-Agyeman F, Balevic SJ, Cotten CM, Adler-Shohet F, et al. Exposure-safety relationship for acyclovir in the treatment of neonatal herpes simplex virus disease. Early Hum Dev. 2022;170:105616.
109 Graf RJ, Viviana Hoyos Garcia I, Dendi A, White NO, Pifer T, Harris R, et al. Mucosal Site Detection of Herpes Simplex Virus in Neonates. J Pediatr. 2024;275:114212.
110 Kimberlin DW, Lin CY, Jacobs RF, Powell DA, Corey L, Gruber WC, et al. Safety and efficacy of high-dose intravenous acyclovir in the management of neonatal herpes simplex virus infections. Pediatrics. 2001;108:230-8.
111 Kimberlin DW, Whitley RJ, Wan W, Powell DA, Storch G, Ahmed A, et al. Oral acyclovir suppression and neurodevelopment after neonatal herpes. N Engl J Med. 2011;365:1284-92.
112 Kotzbauer D, Frank G, Dong W, Shore S. Clinical and laboratory characteristics of disseminated herpes simplex virus infection in neonates. Hosp Pediatr. 2014;4:167-71.
113 Lao S, Flagg EW, Schillinger JA. Incidence and Characteristics of Neonatal Herpes: Comparison of Two Population-Based Data Sources, New York City, 2006-2015. Sex Transm Dis. 2019;46:125-31.
114 Long SS, Pool TE, Vodzak J, Daskalaki I, Gould JM. Herpes simplex virus infection in young infants during 2 decades of empiric acyclovir therapy. Pediatr Infect Dis J. 2011;30:556-61.
115 Mejias A, Taveras J, Dendi AA, Pifer T, Crisan R, Sanchez PJ. 1751. The Conundrum of Neonatal Herpes Simplex Virus (HSV) Disease: Co-infections do Happen! Open Forum Infectious Diseases. 2023;10:S815.
116 Melvin AJ, Mohan KM, Vora SB, Selke S, Sullivan E, Wald A. Neonatal Herpes Simplex Virus Infection: Epidemiology and Outcomes in the Modern Era. J Pediatric Infect Dis Soc. 2022;11:94-101.
117 Nahmias AJ, Dowdle WR, Josey WE, Naib ZM, Painter LM, Luce C. Newborn infection with Herpesvirus hominis types 1 and 2. J Pediatr. 1969;75:1194-203.
118 O'Riordan DP, Golden WC, Aucott SW. Herpes simplex virus infections in preterm infants. Pediatrics. 2006;118:e1612-20.
119 Pass RF, Dworsky ME, Whitley RJ, August AM, Stagno S, Alford CA, Jr. Specific lymphocyte blastogenic responses in children with cytomegalovirus and herpes simplex virus infections acquired early in infancy. Infect Immun. 1981;34:166-70.
120 Schillinger JA, Washburn K, Klingler E, Blank S, Pathela P. Comparison of the clinical and demographic characteristics of neonatal herpes infections caused by herpes simplex virus type 1 and type 2; findings from a population-based surveillance system, 2006-2012. Sexually Transmitted Infections Conference: STI and AIDS World Congress. 2013;89.
121 Sullender WM, Miller JL, Yasukawa LL. Humoral and cell-mediated immunity in neonates with herpes simplex virus infection. Journal of Infectious Diseases. 1987;155(1):28-37.
122 Whitley RJ, Corey L, Arvin A, Lakeman FD, Sumaya CV, Wright PF, et al. Changing presentation of herpes simplex virus infection in neonates. J Infect Dis. 1988;158:109-16.

123 Whitley R, Arvin A, Prober C, Burchett S, Corey L, Powell D, et al. A controlled trial comparing vidarabine with acyclovir in neonatal herpes simplex virus infection. Infectious Diseases Collaborative Antiviral Study Group. N Engl J Med. 1991;324:444-9.
124 Samies N, Jariwala R, Boppana S, Pinninti S. Utility of Surface and Blood Polymerase Chain Reaction Assays in Identifying Infants With Neonatal Herpes Simplex Virus Infection. Pediatr Infect Dis J. 2019;38:1138-40.
125 Yeager Anne S, Arvin Ann M, Urbani Lenore J, Kemp John A. Relationship of Antibody to Outcome in Neonatal Herpes Simplex Virus Infections. Infection and Immunity. 1980;29:532-8.
126 Yeager AS, Arvin AM. Reasons for the Absence of a History of Recurrent Genital Infections in Mothers of Neonates Infected with Herpes Simplex Virus. Pediatrics. 1984;73:188-93.
127 Cruz AT, Freedman SB, Kulik DM, Okada PJ, Fleming AH, Mistry RD, et al. Herpes Simplex Virus Infection in Infants Undergoing Meningitis Evaluation. Pediatrics. 2018;141.
128 Leventon-Kriss S, Rannon L, Smetana Z, Yoffe R. Increase in laboratory-confirmed cases of herpes genitalis and neonatal herpes infections in Israel. Isr J Med Sci. 1983;19:946-9.
129 Lewensohn-Fuchs I, Osterwall P, Forsgren M, Malm G. Detection of herpes simplex virus DNA in dried blood spots making a retrospective diagnosis possible. J Clin Virol. 2003;26:39-48.
130 Malm G, Forsgren M, el Azazi M, Persson A. A follow-up study of children with neonatal herpes simplex virus infections with particular regard to late nervous disturbances. Acta Paediatr Scand. 1991;80:226-34.
131 Malm G, Forsgren M. Neonatal herpes simplex virus infections: HSV DNA in cerebrospinal fluid and serum. Arch Dis Child Fetal Neonatal Ed. 1999;81:F24-9.
132 Tookey P, Peckham C, Lynn R, Brown D. British Paediatric Surveillance Unit, 21st Annual Report 2006-2007. British Paediatric Surveillance Unit, Royal College of Paediatrics and Child Health: 2007.
133 Kashyap A, Farley M, Smith D, Balmer S, Nellissery S, Nayak PP. Renal replacement therapy in critically ILL neonates with systemic herpes infection - A recent single centre experience. Pediatric Critical Care Medicine. 2014;1):4-5.
134 Khandwalla Z, Gupta C, Jhala H. Diagnosis of hemophagocytic lymphohistiocytosis in disseminated neonatal herpes simplex virus infection and its influence on outcome. Pediatric Critical Care Medicine. 2021;22(SUPPL 1):85.
135 Vaughan E, Balboa PL, Martinez A, Ravenscroft J. PA08 Extensive congenital epidermal skin loss with sparing of the palms, soles and head: strikingly similar appearances in four patients with neonatal herpes simplex infection. British Journal of Dermatology. 2024;191:i126-i.
136 Waheed S, Nuttall L, Fidler K, Dudley J, Bamford A, Lyall H. Neonatal Herpes Simplex Virus: Cutaneous Recurrence Is Common on Stopping Prophylactic Suppression Therapy. J Pediatric Infect Dis Soc. 2022;11:518-21.
137 Tiffany KF, Benjamin Jr DK, Palasanthiran P, O'Donnell K, Gutman LT. Improved neurodevelopmental outcomes following long-term high-dose oral acyclovir therapy in infants with central nervous system and disseminated herpes simplex disease. Journal of Perinatology. 2005;25(3):156-61.

138 Jones C, Isaacs D, McIntyre P, Cunningham T, Garland S. Australian Paediatric Surveillance Unit, Annual Report 2004. Australian Paediatric Surveillance Unit: 2005.
139 Jones CA, Raynes-Greenow C, Isaacs D. Population-based surveillance of neonatal herpes simplex virus infection in Australia, 1997-2011. Clin Infect Dis. 2014;59:525-31.
140 Malouf DJ, Oates RK. Herpes simplex virus infections in the neonate. J Paediatr Child Health. 1995;31:332-5.
141 Nunez CA, Morris A, Teutsch SM, McGregor S, Brotherton J, Novakovic D, et al. Australian Paediatric Surveillance Unit Annual Report 2018. Commun Dis Intell (2018). 2019;43.
142 Kawada J, Kimura H, Ito Y, Ando Y, Tanaka-Kitajima N, Hayakawa M, et al. Evaluation of systemic inflammatory responses in neonates with herpes simplex virus infection. J Infect Dis. 2004;190:494-8.
143 Kimura H, Ito Y, Futamura M, Ando Y, Yabuta Y, Hoshino Y, et al. Quantitation of viral load in neonatal herpes simplex virus infection and comparison between type 1 and type 2. J Med Virol. 2002;67:349-53.
144 Okanishi T, Yamamoto H, Hosokawa T, Ando N, Nagayama Y, Hashimoto Y, et al. Diffusion-weighted MRI for early diagnosis of neonatal herpes simplex encephalitis. Brain Dev. 2015;37:423-31.
145 Kim D, Choi JS, Park JY, Park SE, Lee BK, Lee H, et al. A multicenter study on clinical outcome of symptomatic neonatal herpes simplex virus infection in Korea. Open Forum Infectious Diseases. 2018;5(Supplement 1):S696-S7.
